# Terrestrial planet and asteroid belt formation by Jupiter–Saturn chaotic excitation

**DOI:** 10.1038/s41598-023-30382-9

**Published:** 2023-03-27

**Authors:** Patryk Sofia Lykawka, Takashi Ito

**Affiliations:** 1 Kindai University, Shinkamikosaka 228-3, Higashiosaka, Osaka 577-0813 Japan; 2 Center for Computational Astrophysics, National Astronomical Observatory of Japan, Osawa 2-21-1, Mitaka, Tokyo 181-8588 Japan; 3 Planetary Exploration Research Center, Chiba Institute of Technology, 2-17-1 Tsudanuma, Narashino, Chiba 275-0016 Japan

**Keywords:** Inner planets, Asteroids, comets and Kuiper belt, Planetary science, Early solar system

## Abstract

The terrestrial planets formed by accretion of asteroid-like objects within the inner solar system’s protoplanetary disk. Previous works have found that forming a small-mass Mars requires the disk to contain little mass beyond ~ 1.5 au (i.e., the disk mass was concentrated within this boundary). The asteroid belt also holds crucial information about the origin of such a narrow disk. Several scenarios may produce a narrow disk. However, simultaneously replicating the four terrestrial planets and the inner solar system properties remains elusive. Here, we found that chaotic excitation of disk objects generated by a near-resonant configuration of Jupiter–Saturn can create a narrow disk, allowing the formation of the terrestrial planets and the asteroid belt. Our simulations showed that this mechanism could typically deplete a massive disk beyond ~ 1.5 au on a 5–10 Myr timescale. The resulting terrestrial systems reproduced the current orbits and masses of Venus, Earth and Mars. Adding an inner region disk component within ~ 0.8–0.9 au allowed several terrestrial systems to simultaneously form analogues of the four terrestrial planets. Our terrestrial systems also frequently satisfied additional constraints: Moon-forming giant impacts occurring after a median ~ 30–55 Myr, late impactors represented by disk objects formed within 2 au, and effective water delivery during the first 10–20 Myr of Earth’s formation. Finally, our model asteroid belt explained the asteroid belt’s orbital structure, small mass and taxonomy (S-, C- and D/P-types).

## Introduction

Early terrestrial-planet formation models postulated that the terrestrial planets formed in a protoplanetary disk that extended until the disk outer edge at ~ 4–5 au (ref.^1,2^). However, it was later found that these models could not explain the small mass of Mars^1–5^. Since this constraint is essential to explaining the inner solar system, we focus only on models capable of explaining Mars’ mass in this work. A common view in recent studies is that ~ 2 Earth masses (ME) of mass concentrated in a narrow disk at 0.7–1.0 au to form Venus and Earth^6^. In this paradigm, nearly formed Mercury and Mars were scattered out from this region and remained of low mass due to a lack of disk mass within 0.7 au and beyond 1 au (ref.^7–10^). Other models suggest that both planets formed across a similarly narrow disk in tandem with the Venus–Earth pair by the end of disk gas dispersal^11^. A narrow disk could have resulted from disk-gas-driven convergence of small bodies during the solar system’s first Myr, resulting in highly mass concentrated rings near the Venus–Earth region^12–14^. Alternatively, disk-gas-driven inward-then-outward Jupiter migration (Grand Tack) or giant-planet instability (henceforth ‘instability’) could also have created narrow disks by dynamical truncation of the disk beyond ~ 1.5 au (ref.^8,9^). Another key question is how the origin of this narrow disk (or the terrestrial planet system) is related to asteroid belt formation. The asteroid belt consists of asteroids concentrated at semimajor axes *a* = 2–3.25 au possessing a wide range of eccentricities (*e* < 0.4) and inclinations (*i* < 35°). Furthermore, the total mass of the asteroid belt is only 5 × 10^−4^ ME (ref.^15^). It is unclear whether the primordial asteroid belt was dynamically depleted (e.g., by Jupiter)^8,16,17^ or was not massive originally^12,13,18^. The asteroid belt holds essential clues regarding the nature of the protoplanetary disk that formed the terrestrial planets.

In addition to the orbits and masses of the four terrestrial planets, other essential constraints in the inner solar system include the planets’ formation timescales and accretion history (e.g., giant impacts), Moon formation (e.g., timing) and mass accreted by Earth after that, nature of the planets’ late impactors, origin and accretion evolution of water on all the four terrestrial planets, among others. Furthermore, the absence of planets, the orbital architecture (including the peculiar concentration of asteroids with *i* < 20 deg), compositional taxonomy, and the low mass in the asteroid belt represent additional fundamental constraints (Supplementary Information 1). While the Grand Tack^8^ and the early instability^6,9^ models investigated several constraints related to the terrestrial planets and the asteroid belt, other models^7,10,11,13^ addressed these issues in much less depth (e.g., focusing only on a few chosen constraints from the list above). As discussed below, several models in the literature also neglected Mercury formation (e.g., the Grand Tack). Furthermore, forming the asteroid belt in tandem with the terrestrial planets remains poorly understood. Therefore, there has been insufficient discussion about explaining *simultaneously* the orbits, masses and other constraints of the four terrestrial planets and the asteroid belt in the literature^6,16,19–21^.

Noteworthy, only a few inner solar system models have tackled the formation of terrestrial planets and the asteroid belt in a single evolutionary fashion (e.g., the Grand Tack and early instability models). Despite these models’ new insights and successes, we discuss several issues related to them that are absent or possibly less relevant in our model. We also argue that the 0.7–1.0 au narrow disk (henceforth ‘canonical annulus’) model is not an adequate baseline for terrestrial planet formation. Other models face important challenges and lack discussions regarding the inner solar system constraints, so they are not discussed below. See Supplementary Information 2 for details. In this work, we considered the simultaneous formation of terrestrial planets and the asteroid belt. Namely, we addressed all the inner solar system constraints described above with unprecedented detail, thus making our model substantially comprehensive.

We performed N-body simulations of the early solar system consisting of the giant planets and an extended massive protoplanetary disk after gas dispersal. In our 650 main simulations, the pre-instability Jupiter–Saturn pair experienced their mutual near 2:1 mean motion resonance (MMR) on moderately eccentric orbits. The Methods section demonstrates that this orbital configuration was plausible and probably arose naturally before the instability. In particular, the presence of Mars-Earth-mass bodies or additional planets in the outer solar system probably played a role in originating this orbital configuration. Noting that the instability probably occurred in ~ 10 Myr timescales after the solar system’s gas dispersal^22–24^ (see also “Methods”), for simplicity, we assumed that this near-resonant stage operated in similar timescales before the instability in our simulations. In the next stage (post-instability), we took the orbital state of all disk objects at the end of the previous stage and placed Jupiter and Saturn on their near-current orbits. We justify this simplification of the instability in Methods. We then followed the evolution of these terrestrial systems until 400 Myr. In these simulations, the protoplanetary disk consisted of a small number of embryos (Moon–Mars-mass objects) and several planetesimals (small asteroid-like objects) that concentrated at smaller distances and up to 3.5 au in the disk, respectively. Consistent with predictions of embryo/planetesimal formation models, implications from our previous results, and some fundamental constraints about the terrestrial planets, we tested several variations in disk properties. This procedure resulted in our distinct disk models, as illustrated in Fig. S1 and summarised in Table S1. The disks comprised a core region surrounded by a less massive inner region and an extended outer region. In all disks modelled, the embryos and planetesimals started on nearly circular and coplanar orbits. The disk component beyond 2 au consisted of planetesimals and represented the primordial asteroid belt (local asteroids), which was thousands of times more massive than the current. We also investigated the existence of spectral classes inspired by asteroid taxonomy (S, C and D/P types) and distinct water mass fractions for our disk objects (“Methods” and Table S2). Finally, we used a rigorous classification algorithm to properly identify analogues of the terrestrial planets (“Methods” and Supplementary Information 2.1).

**Figure 1:**
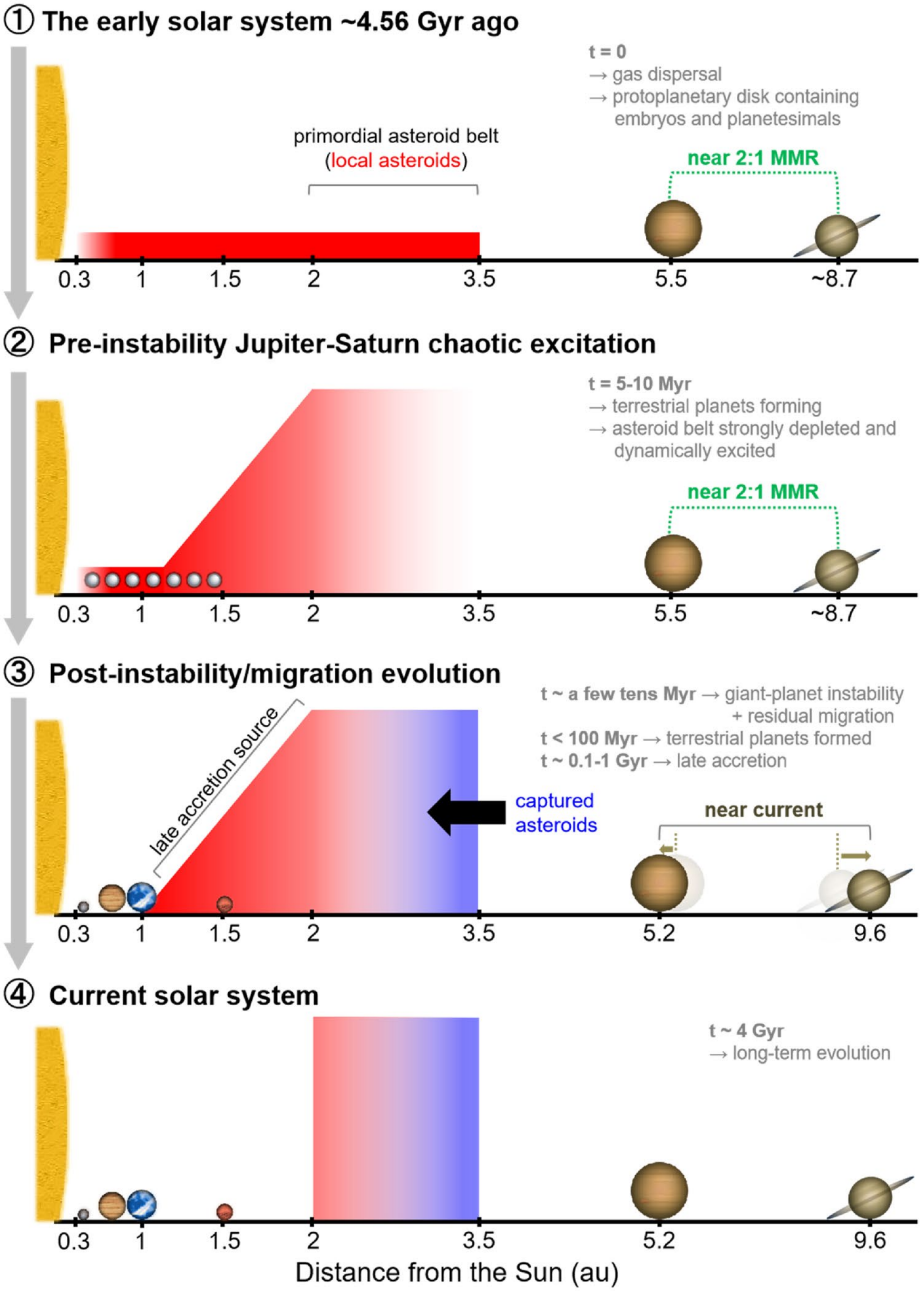
Outline of the Jupiter–Saturn chaotic excitation scenario for the formation of the terrestrial planets and the asteroid belt. Four main stages describe the dynamical history of the solar system. The approximate duration of key events is denominated by *t*. See the main text and Supplementary Information for the details.

Furthermore, we used additional long-term simulations to investigate the formation of the asteroid belt. Specifically, we built a representative asteroid belt consisting of local and captured asteroids. The local asteroids were obtained in systems containing good representatives of the terrestrial planets from the main simulations described above. The captured asteroids were obtained from simulations of trans-Jovian objects captured in the asteroid belt during the instability/migration of the giant planets. Finally, we took the orbital states of local and captured asteroids at *t* ~ 100 Myr and evolved them until 4 Gyr. We also tested the influence of the instability and post-instability residual migration on our results based on auxiliary simulations. Figure 1 summarises the timeline of the main events envisioned in our scenario.

Consult Methods and Supplementary Information 3 for more details about our simulations and their initial conditions.

## Results and discussions

### Truncating the protoplanetary disk before the instability

First, when placing the Jupiter–Saturn pair on a near 2:1 MMR configuration, Saturn’s natural frequencies associated with the longitude of perihelion and the longitude of ascending node wander chaotically over a wide range of values (Fig. S2 and Supplementary Information 4). Thus, several asteroids are affected by the associated nu6 and nu16 secular resonances resulting in widespread orbital excitation in the asteroid belt^25^. Here, we found that this Jupiter–Saturn chaotic excitation (JSCE) mechanism offers a new route to generate a narrow disk and has far-reaching implications for the inner solar system. Considering that the strength of the nu6 resonance is sensitively proportional to planetary eccentricities and because the orbits of our Jupiter–Saturn pairs were more eccentric than that assumed in past work^25^, a stronger nu6 resonance operated in the disk^4^. As a result, JSCE strongly depleted the system’s disk beyond ~ 1–1.5 au (up to ~ 3.5 au), as most asteroids acquired large eccentricities leading to gravitational scattering by the giant planets and collisions with the Sun (Figs. S3, S4). These results demonstrate that moderately eccentric Jupiter and Saturn experiencing JSCE could both excite and deplete the primordial asteroid belt. Consequently, violent instabilities (e.g., the early instability model) or complex Grand-Tack-like scenarios are not required to truncate a massive extended disk. It is also unnecessary to assume that the disk was initially narrow^7,18^ (i.e., with “empty” outer regions beyond ~ 1 au), which assumes that planetesimal formation occurred only under special conditions at a specific desirable and narrow region of the disk^11–14,26,27^. Instead, the only requirement in the JSCE scenario is that moderately eccentric Jupiter and Saturn experience their 2:1 MMR prior to the instability. Thus, our scenario probably requires less fine-tuning to operate. Another implication is that the instability occurred when the disk was already strongly perturbed beyond ~ 1 au.

### Terrestrial planet analogue systems

**Figure 2:**
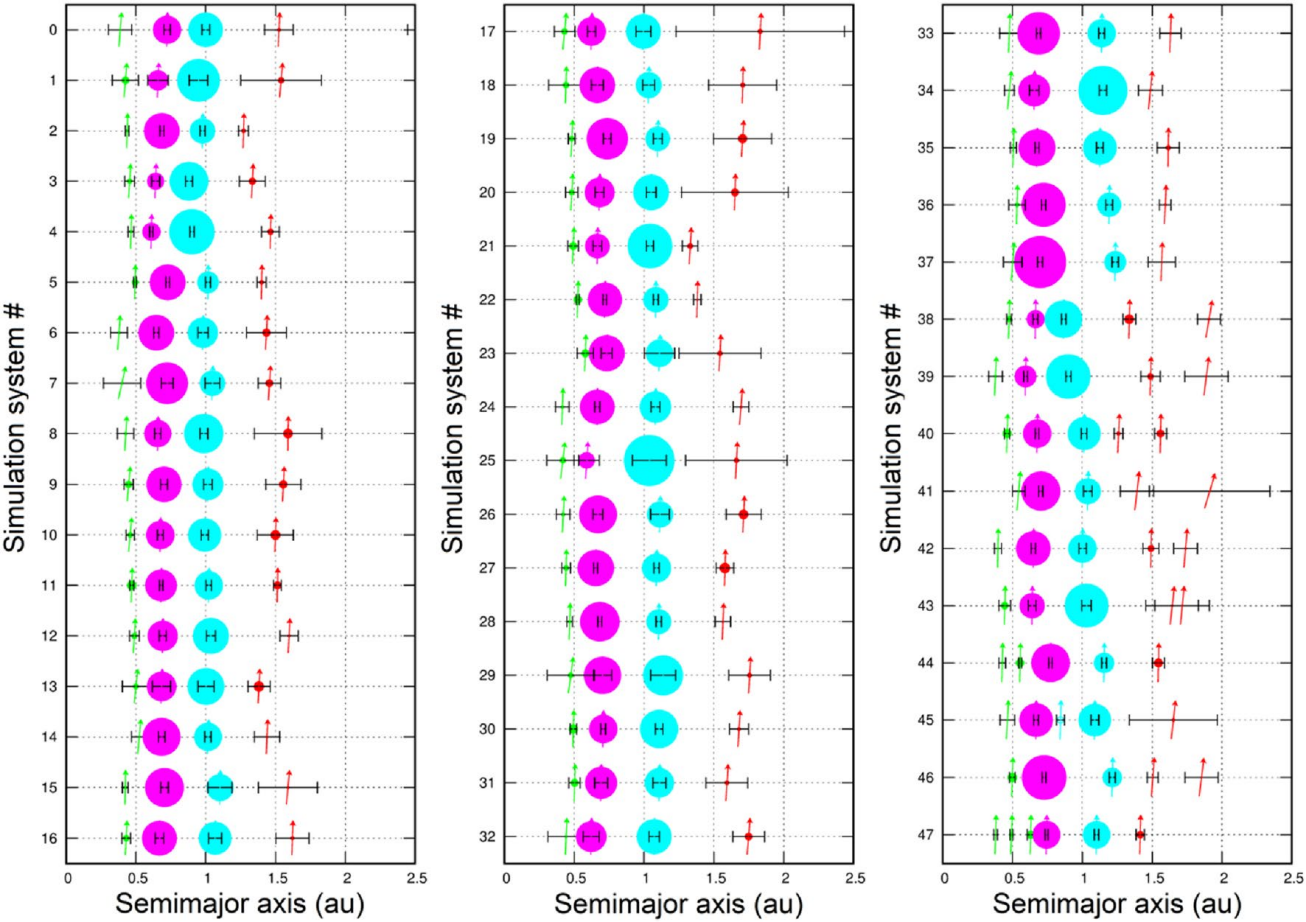
Comparison of 47 individual analogue systems containing representative planet analogues of each terrestrial planet with the solar system planets (system #0). Planet analogues of Mercury, Venus, Earth and Mars are indicated by green-, magenta-, cyan- and red-filled symbols, respectively. The inclination *i* of the planets is represented by the angle between the vector and the perpendicular (e.g., the vector points to the top for *i* = 0°). The error bars indicate the variation in heliocentric distance based on the object’s perihelion and aphelion. The radii of planet symbols scale in proportion to mass.

**Figure 3:**
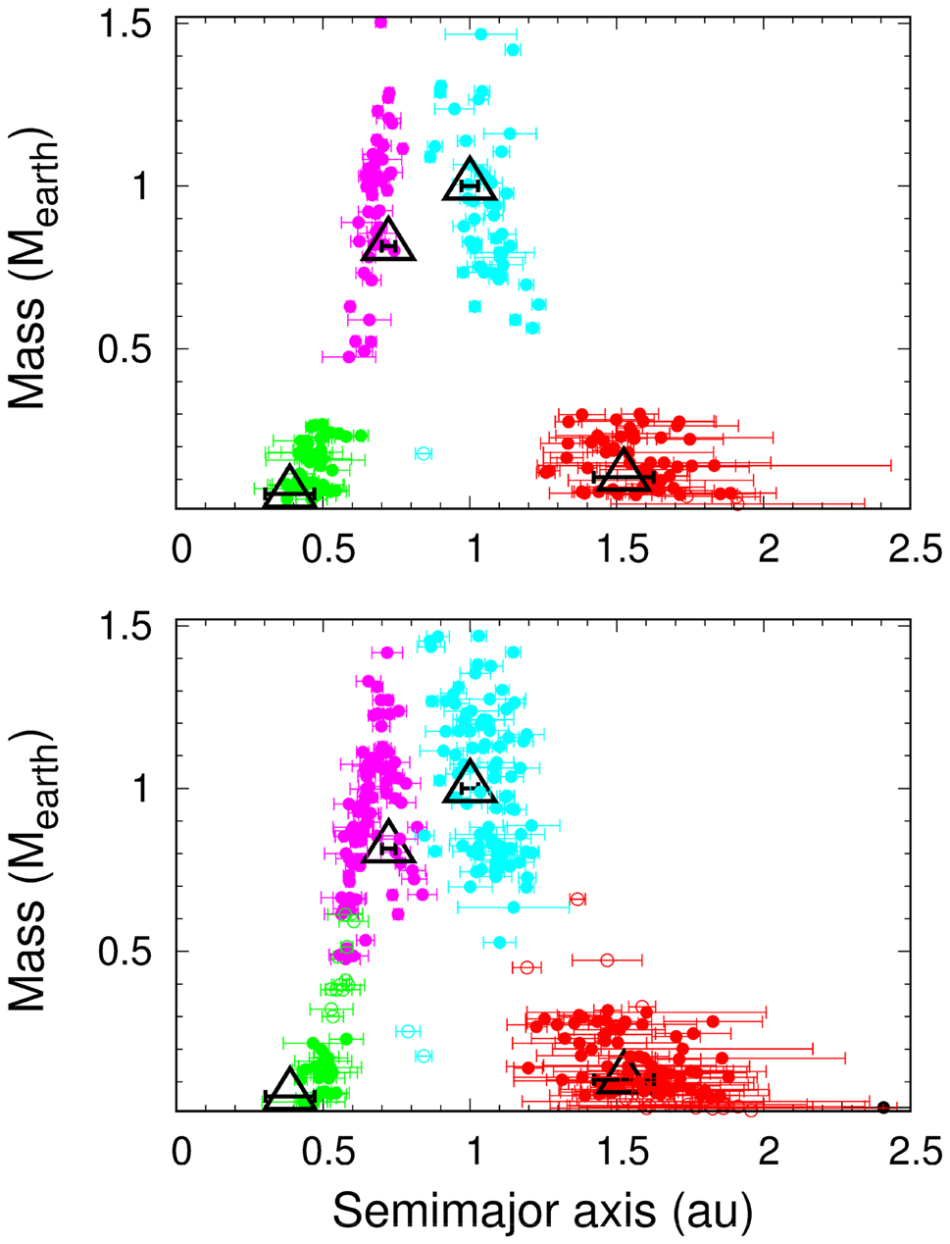
Planets formed in 47 four-planet systems obtained from all simulations (top) and in analogue systems containing three or four representative terrestrial planets from simulations of the standard disk (bottom). Planet analogues of Mercury, Venus, Earth and Mars are indicated by green-, magenta-, cyan- and red-filled symbols, respectively. The error bars indicate the variation in heliocentric distance based on the object’s perihelion and aphelion. The large open triangles represent the terrestrial planets of the solar system.

Our terrestrial planet classification algorithm carefully identified terrestrial planets/systems. In total, our main simulations revealed 221 terrestrial systems containing at least Venus, Earth and one additional planet analogue in each system. Among these, we identified a significant number of systems containing planet analogues of Mercury, Venus, Earth and Mars in the same system (47) (henceforth ‘4-P system’), which closely resembled the four terrestrial planets in terms of orbits and masses (Figs. 2, 3). Furthermore, these systems were excellent representatives of our own because they satisfied many additional vital constraints of the inner solar system (Introduction). Remarkably, some systems could satisfy several of these constraints simultaneously. Overall, our Venus and Earth analogues acquired dynamically cold orbits, while our Mercury and Mars analogues acquired hotter orbits. Indeed, their median *a*-*e*-*i* orbital elements were very similar to those of the real planets. The orbit-mass distribution of these analogues also reproduced the dichotomy of two massive planets (Venus and Earth) surrounded by two much less massive planets (Mercury and Mars) on relatively distant orbits. Other terrestrial system properties are summarised in Supplementary Information 5.1 and Tables S3, S4 and S5. Although our Mercury–Venus pairs formed on average closer to each other than in reality, our Venus–Earth and Earth–Mars pairs matched observations quite well. Regardless, these mutual orbital separations were better than those obtained for narrow disks based on the canonical annulus model (Supplementary Information 2.2 and 5.2). Finally, improving our pioneer research on 4-P systems^19^, we obtained a significant number of 4-P systems analogous to the terrestrial planets and statistically compared them to many inner solar system constraints.

### Formation of Mercury, Venus, Earth and Mars

Although the formation of Mercury is an outstanding problem often neglected in the literature (Supplementary Information 2.3), our model produced a significant number of Mercury analogues (122) (Table S4) belonging to systems analogous to the inner solar system, of which 47 were found in 4-P systems. In addition to increasing the chances of Mercury formation with high efficiency (84/250 = 34%), the inclusion of extended inner regions at 0.3–0.85 au (combined disk Ix) also produced more 4-P systems with 13% efficiency (33/250) compared to 3.5% (14/400) for other disks combined (disk A–E) (Methods). These results significantly improved the probabilities of successfully forming Mercury and 4-P systems compared to past work (Supplementary Information 2.2 and 2.3). Mercury analogues acquired medians *a* = 0.44 (more consistent with *a* = 0.39 au for Mercury) and 0.49 au, *e* = 0.07 and 0.11, *i* = 3.5° and 4.7° and *m* = 0.16 and 0.14 ME for disks Ix and A–E, respectively. In particular, disks containing 0.20–0.25 ME inner regions yielded analogue masses within only a factor of 2 of that of Mercury (disks B, C, Ia, Ic in Table S4). Thus, slightly less massive inner regions could explain Mercury’s current mass. Mercury’s current hot orbit (*e* = 0.21; *i* = 6.8°) can be explained by post-instability residual migration of the giant planets (Supplementary Information 3.3) or Gyr-long-term chaotic dynamics^28^. Overall, disk Ix produced our best Mercury analogues. These analogues started within the inner region with typically 10% of their final masses. Later, they accreted the remaining 35% and 55% from objects in the inner and distant regions (> 0.85 au), respectively. In particular, the contribution of the inner region to their final masses was ~ 3 and ~ 5.5 times higher compared to that for analogues of Venus and Earth/Mars. Also, 10% of the analogues’ final masses originated from objects located beyond 1.5 au (but < 1% beyond 2 au), implying that these objects probably sourced water and other volatile materials to Mercury, in agreement with recent measurements^29,30^. This result supports the hypothesis that the disk was not “empty” beyond 1.5 au. Such a significant contribution of local and mixing of distant objects could also offer new insights into the origin of Mercury’s peculiar physical properties. JSCE did not shape the inner region but affected Mercury’s formation (e.g., delivery of water, accretion history of distant objects, etc.). In short, forming Mercury analogues is an essential *additional result* that makes our scenario more comprehensive (see also Supplementary Information 5.2). We conclude that the formation of Mercury probably required a low-mass inner region and a disk outer region extending beyond 1 au in the disk.

In general, our analogue systems contained Venus–Earth pairs with orbits and masses in agreement with the real planets (Table S4). The Venus and Earth analogues acquired medians of *a* ~ 0.65–0.67 au (Venus), *a* ~ 1.00–1.07 au (Earth) and *e* ~ 0.03–0.04, *i* ~ 2° and *m* ~ 0.82–1.03 ME for both planets. Concerning the mutual distance of Venus and Earth (*a*_E_-*a*_V_ = dVE = 0.28 au), a Venus–Earth pair was considered successful if their mutual distance fell within the range 0.67–1.33 × dVE. Approximately 40–50% and 70% of the Venus–Earth pairs acquired successful mutual distances in disks considering initial mass concentrations within 0.8–1.0 au (disks B and C) and 0.85–0.95 au (disk Ix), respectively. Thus, reproducing the Venus–Earth distance likely requires more than 1 ME of mass concentrated in a thin annulus within the disk’s core region. This peculiar feature might also explain the small Venus and Earth analogues’ eccentricities (Table S4). These results roughly match the Venus–Earth pair’s cold orbits, masses and orbital separation. This accomplishment is significant because it is still challenging to correctly reproduce the Venus–Earth’s orbit-mass distribution (ref.^31^ and Supplementary Information 2). Finally, our results are consistent with the terrestrial planets’ late accretion and planetary bulk compositions of both Earth and Mars. Further details are discussed in Supplementary Information 5.3, 5.4 and 5.5.

Our model was highly successful regarding Mars formation, as 146 Mars analogues acquired medians of *a* ~ 1.55–1.58 au, *e* ~ 0.07–0.09, *i* ~ 4°–6° and *m* ~ 0.13–0.20 ME in our analogue systems (Table S4). JSCE’s mass depletion and orbital stirring beyond 1–1.5 au that arose on ~ 5–10 Myr timescales allowed many Mars analogues to acquire small masses and non-cold orbits in similar timescales. Both the Grand Tack and the early instability models require extreme perturbations on short timescales and precise timings to provide the necessary disk depletion to explain the small mass of Mars. In contrast, no such perturbations are required in our scenario to form Mars. The existence of a massive outer region also caused our Mars analogues to form farther from their Earth counterparts, in agreement with observations. Models in which an outer region beyond ~ 1 au starts mostly “empty” tend to form Mars too close to Earth (Supplementary Information 2). Briefly, our model can reproduce the moderately excited orbit of Mars, its small mass and its mutual distance from Earth. In contrast, other models typically focus on the small-mass problem only^6–11,13,18,32–35^. Our Mars analogues accreted 80% (90%) of their final masses after a median of 15–18 Myr (20–27 Myr). These timescales are a bit long but marginally consistent with the ~ 15–23 Myr bulk formation timescale of Mars. The analogues also experienced giant impacts during their accretion histories, which is in line with evidence suggesting that Mars formed in a protracted manner. See also the discussion in Supplementary Information 5.6.

Concerning Moon formation and late veneer mass delivered to Earth, several of our Earth analogues experienced successful late Moon-forming giant impacts (GIs) (Table S5). For a plausible impactor-to-target-mass ratio (ITR) greater than 0.05 (0.02), 55–60% (55–80%) of these analogues experienced GIs after the minimum 25 Myr. Even for the canonical ITR > 0.10, the successful fraction was relatively high: 35–40%. For ITR > 0.05 (0.02), the last GIs occurred after medians of 30–45 (45–55) Myr. Although the Moon-forming timing has large uncertainties (Supplementary Information 1), these results are consistent with a < 60-Myr Moon-forming interval based on isotopic systematics^36^ and the 45-Myr timing derived from hafnium-tungsten chronometry^37^. After the last GI, overall 30–40% (40–50%) of our Earth analogues accreted less than 1% of their final masses for ITR > 0.05 (0.02), whilst for ITR > 0.10 the results were 20–25%. It is clear from these results that less massive Moon-forming impactors with a mass of 0.02 ME < *m* < 0.1 ME can increase the success rates of satisfying both the Moon-formation timing and Earth’s late veneer mass constraints. Such conditions are consistent with models of Moon formation. These success rates are also higher than those obtained for the canonical annulus and similar models (Supplementary Information 2.2 and 2.6). As JSCE stirred the orbits of objects located beyond ~ 1–1.5 au, collisions with Earth became less likely, and Moon-forming GIs occurred later, resulting in fewer remnant objects to later accrete.

Furthermore, after considering 80 water mass fraction (WMF) models that probed different water contents in specific regions of the disk (Table S2), we found that the often assumed WMF models used in the literature^4,9,19^ (models 1, 2 and 16 in Table S2) failed to satisfy the water contents of Venus, Earth and Mars simultaneously. Instead, objects with higher WMFs beyond ~ 1–1.5 au were required to satisfy this constraint (Supplementary Information 5.7). These results imply that this region was relatively massive and wetter than previously thought. Such conditions provided enough water content to the terrestrial planets. The results are also consistent with recent observations of water on enstatite chondrite meteorites^38^ and S-type asteroids^39^, and models of water-rich objects implanted into the primordial asteroid belt^40^. The amount of water delivered to an Earth analogue was a median of 2–5 times the mass in the oceans. This result lies within the typical ~ 2–10 oceans estimated for Earth’s water mass^38,41^. Therefore, the origin of Earth’s bulk water could be explained by collisions with water-rich objects located beyond ~ 1.5–2 au after being quickly stirred by JSCE. This process allowed Earth’s water to be delivered in less than 10–20 Myr of the planet’s accretion history. These results agree with isotopic evidence that most of Earth’s water was acquired early (before the Moon-forming GI) and not during late accretion^41^. Finally, in agreement with isotopic constraints^42^, we found that the Earth analogues acquired 80% (90%) of their final masses within 20 Myr (35 Myr) and the remaining mass in longer timescales, often experiencing giant impacts. In short, the JSCE model can explain the Earth’s fast water delivery and protracted formation.

### Formation of the asteroid belt

**Figure 4:**
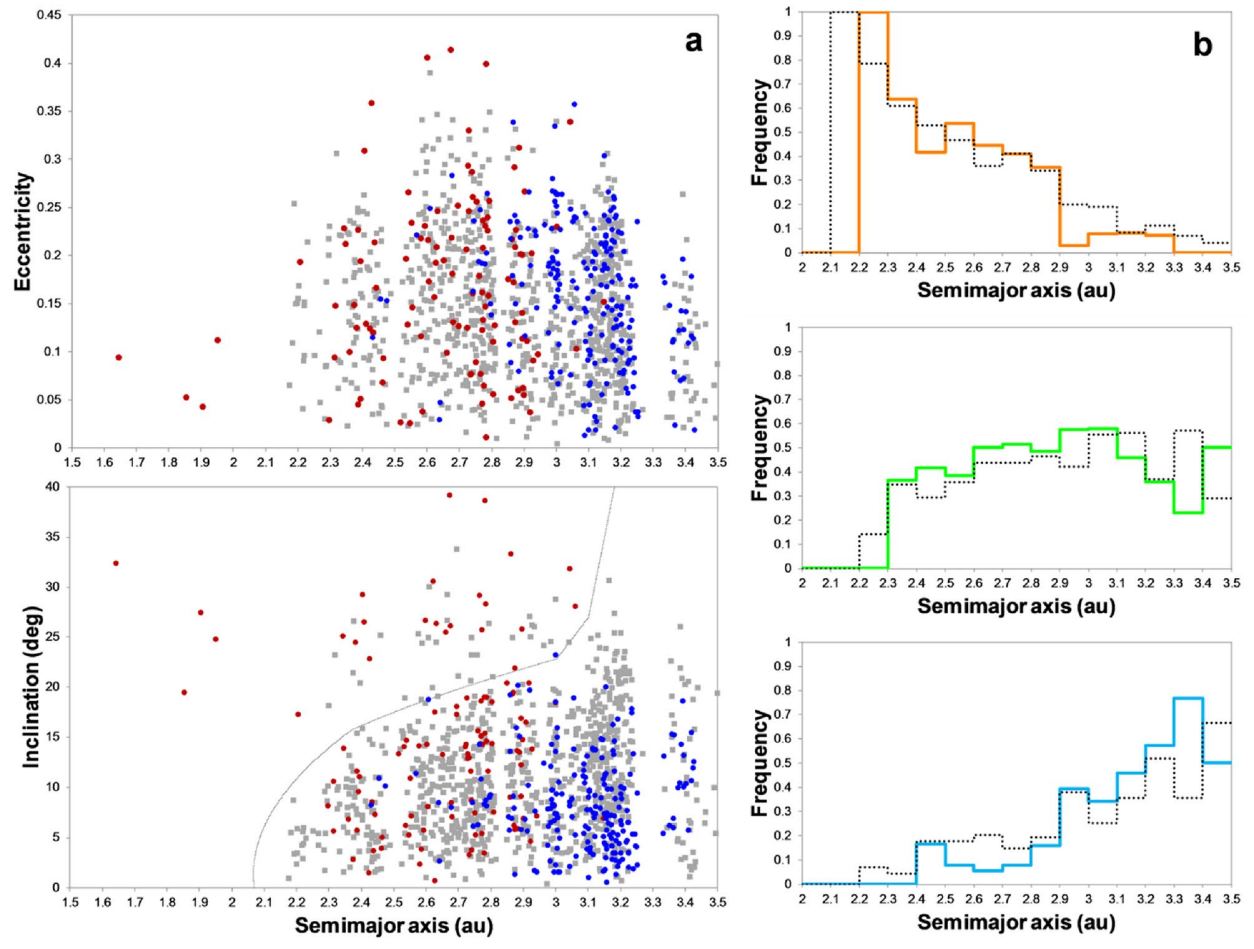
Comparison of our representative asteroid belt constructed based on disk models that yielded terrestrial planet systems analogous to our own (coloured symbols/curves) with 895 large observed asteroids not belonging to asteroid families (greyscale symbols/curves). Model asteroids represent the state after ~ 4.1 Gyr of dynamical evolution. Observed asteroids possess diameters D > 20–30 km (for albedos 0.1–0.2). (**a**) The representative asteroid belt consists of local primordial asteroids in the protoplanetary disk at < 3.5 au (brown symbols) and asteroids captured from trans-Jovian orbits after giant-planet migration (blue symbols). The proportion of local:captured asteroids is 33%:67%. The curve indicates the location of the secular resonance nu6 (bottom). (**b**) Both model and observed asteroids are classified as S- (top), C- (middle) and D/P-type asteroids (bottom). The histograms show the relative fractions of asteroid types per semimajor axis bin. The composition model considered the following proportions of S-, C-, D/P-asteroids: S80%-C20% (< 2 au) and S50%-C50% (> 2 au) for local and S5%-C47.5%-DP47.5% for captured asteroids. See Methods and Supplementary Information for details.

Our scenario successfully satisfied several constraints in the asteroid belt. Before the onset of the instability, a moderately eccentric Jupiter sculpted the primordial asteroid belt beyond ~ 3.2 au, and JSCE dynamically depleted and stirred the local asteroids, so a tiny fraction of them survived at 2–3.25 au. Our local asteroids were obtained in analogue systems containing three or four representative terrestrial planets from simulations of the standard disk (Fig. 3). Later, the instability and migration of the giant planets destabilised many objects in trans-Jovian reservoirs, a small fraction of which contaminated the primordial asteroid belt^24,43^. At the end of giant-planet migration, captured asteroids from these reservoirs survived in the outskirts of the belt^43,44^. After ~ 4.1 Gyr of evolution, our representative asteroid belt consisted of mixed populations of local and captured asteroids (see “Introduction” and “Methods” for details). Next, we compared these results with observations by considering large asteroids not belonging to asteroid families, which represented the asteroid belt orbital structure well. Overall, as evinced by the similarity of the *a*-*e*-*i* distributions, the orbital structure of our representative asteroid belt broadly reproduced observations (Fig. 4). Our asteroid belt model also featured an unprecedented high resolution compared with similar works^16–18,45^. In agreement with ref.^25^, the orbital spectra of Saturn when JSCE was active revealed that nu6 resonance was more prominent than nu16 resonance (Fig. S2). This behaviour explained the wide range of eccentricities and less-excited inclinations acquired by stable local asteroids. Notably, captured asteroids on stable orbits tend to concentrate below 20° (ref.^43^). Overall, these results may explain the fraction of asteroids with inclinations above the nu6 resonance (f6). Here, after considering the effects of instability, the final stages of giant planet migration, and ~ 4 Gyr long-term evolution, we obtained f6 = 0.11–0.18, which was smaller than the values obtained previously in the literature. These results are marginally compatible with the observed value of 0.07. Therefore, the JSCE scenario may solve the longstanding problem of reproducing f6. More details and other advantages of our asteroid belt model are found in Supplementary Information 2.5 and 5.8. Overall, we conclude that the good match of the orbital structure of our asteroid belt with the observed one is robust.

JSCE depleted 99.87% of local asteroids in the primordial asteroid belt. This result is better than most previous models predicting values ~ 90–99% over the age of the solar system^9,17^ and comparable to ~ 99–99.9% found in the instability model for the asteroid belt^16^. However, all depleted systems with > 97% found in the latter work put Jupiter and Saturn on too-excited or mutually distant orbits (orbital period ratio PS/PJ > 2.8). The final masses of our asteroid belt considering both local and captured asteroids varied within 1–2 × 10^−3^ ME, or ~ 2–4 times the mass in the current asteroid belt, assuming 33–67% of local asteroids represent the latter. This result is compatible with observations because these masses are likely upper limits and could be smaller after considering a less massive primordial asteroid belt or additional depletion mechanisms. Concluding, in addition to the bulk orbital structure, our model can also explain the current small mass of the asteroid belt.

Finally, by considering a primordial asteroid belt consisting of local S- and C-asteroids and captured asteroids akin to C- and D/P-asteroids, we found that our asteroid belt reproduced not only the dichotomy of S- and D/P-type asteroids spread in the inner-middle and outer regions of the belt but also the distribution of C-type asteroids beyond ~ 2.3 au (ref.^15^). Furthermore, the orbital concentrations of our obtained S-, C- and D/P-asteroids approximately matched the concentrations (peaks) with the heliocentric distance of observed asteroids (Fig. 4). Overall, the best results were for an asteroid belt represented by local and captured asteroids in similar proportions (not exceeding a factor of two). The asteroid belt model within the JSCE scenario is the first to successfully explain the orbital distribution of three taxonomic populations represented by S-, C- and D/P-type asteroids.

After the giant planets’ instability/migration and Moon formation, the evolution of asteroids in our long-term simulations indicated that roughly 0.0018 ME of mass was added to the nearly formed Earth via asteroid collisions over 4 Gyr. This value is similar to those based on the constraints of highly siderophile elements^46,47^. As JSCE’s disk planetesimal depletion was faster beyond ~ 2 au, long-lived reservoirs remained within this boundary. Indeed, the decaying population of late Earth impactors comprised asteroids concentrated at 1–2 au and *i* = 15°–50° at the end of the terrestrial-planet formation. Considering that this population originally formed at ~ 0.8–1.8 au in the disk, these results are consistent with isotopic evidence indicating that most of Earth’s late impactors had a dry chondrite nature^48^. Thus, the JSCE scenario can potentially explain Earth’s late veneer mass and the nature of late impactors. Finally, our results also imply that these terrestrial-region asteroids (< 2 au) bombarded the entire inner solar system, in agreement with the findings of ref.^46^. Consult Methods and Supplementary Information 5.8 for further details about asteroid belt formation in our scenario.

### Summary

The JSCE model could explain a large number of inner solar system constraints. Tentative implications of our scenario are summarised below. First, at the end of gas dispersal, the protoplanetary disk that formed the terrestrial planets was probably massive and comprised three main components: a low-mass (< 0.20–0.25 ME) and extended inner region that was crucial for the formation of Mercury; a massive (~ 2 ME) core region at ~ 0.8–1.2 au possessing a mass concentration within a thin layer (> 1 ME within dr < 0.2 au) that allowed the formation of Venus and Earth on mutually close orbits; and a massive adjacent outer region (up to 3.5 au) containing the primordial asteroid belt that was strongly excited/depleted by JSCE over ~ 5–10 Myr timescales before the instability, which later allowed the formation of Mars. Finally, the current asteroid belt consists of remnant primordial asteroids and captured asteroids from trans-Jovian regions. See also Supplementary Information 6.

The main highlights of our work based on the JSCE scenario are the following.

- A new mechanism to mass depletes a protoplanetary disk is proposed. It is based on the Jupiter–Saturn chaotic excitation generated when both planets evolve on moderately eccentric orbits near their 2:1 MMR before giant planet instability;
- New scenarios to explain a small-mass Mars and a low-mass asteroid belt are proposed as natural consequences of the disk depletion of planetesimals beyond ~ 1.5 au;
- A new model of terrestrial planet formation is proposed based on the same disk above and the addition of an inner region within ~ 0.8–0.9 au. After ~ 100 Myr of post-instability evolution, this model forms terrestrial systems containing the four terrestrial planets with orbits and masses similar to the observed;
- The Earth analogues obtained in the modelled systems often satisfy important constraints: Moon formation timing, early accretion of water-rich asteroids (Earth’s bulk water), and late accretion of dry asteroids;
- A new model of asteroid belt formation is proposed based on the admixing of local and captured asteroids that survived ~ 4 Gyr of evolution after terrestrial planet formation. Local asteroids are remnant disk objects after JSCE depletion. Captured asteroids contaminated the disk from trans-Jovian reservoirs after the instability. This model broadly explains the asteroid belt’s low mass, orbital distribution (*a*-*e*-*i*), and composition taxonomy of three major populations (S-, C-, and D/P-types);
- From the results above, we infer that the inner solar system’s bombardment was probably caused by remnant dry asteroids from the tail-end of terrestrial planet formation.

## Methods

### Basic assumptions

Our simulated systems started when the disk gas had already decayed, so its dynamical influence was negligible. The solar system nebular gas dissipated in ~ 4 Myr (ref.^49^), so for simplicity, we set 5 Myr as time zero in our scenario. At this time, the protoplanetary disk contained the newly formed giant planets, a small number of embryos, and many planetesimals^1,3,4,32,50^. Thus, following the methods used in several similar studies^1,3–6,9,19,21^, we considered a disk containing embryos (lunar-Mars-mass objects), residual planetesimals and the Jupiter–Saturn pair with their current masses.

### Jupiter–Saturn: pre-instability orbital properties

Hydrodynamic models of the early solar system when the disk gas was present found that Jupiter and Saturn can typically interact with their mutual 3:2 or 2:1 mean motion resonance (MMR)^51–55^. After the gas dispersal in the Jovian region, this orbital configuration was probably short-lived^23,56^ and Jupiter, Saturn, and other giant planets in the outer solar system experienced a brief phase of dynamical instabilities^22,24,51^ (henceforth ‘instability’). Jupiter and Saturn then acquired their current orbits after a final phase of residual migration^24,51^. The post-instability evolutions of the Jupiter–Saturn 3:2 MMR orbital configuration have been explored in detail based on success metrics of fundamental aspects of the outer solar system^51^. However, recent work^57,58^ showed that they might be better reproduced by a 2:1 MMR configuration with Jupiter and Saturn on moderately eccentric orbits (≤ 0.05). During the instability, close encounters with a Uranus- or Neptune-class icy giant may be needed to explain the current eccentricities of Jupiter and Saturn^51^. These results assume that Jupiter and Saturn had nearly circular orbits before the instability. However, at the end of gas dispersal, both planets may have inherited eccentric orbits^51–55,57,58^, or acquired them by interactions with a self-gravitating disk^22^ or massive primordial objects, as shown below.

Following this reasoning, we assumed that Jupiter and Saturn evolved to a 2:1 MMR acquiring non-zero eccentricities prior to the instability. The following evidence supports the hypothesis that both planets acquired a near-MMR configuration characterised by orbital period ratio PS/PJ ~ 2 and large libration amplitudes (~ 300–360°). First, a metastable near-MMR configuration is consistent with the well-supported idea that the instability occurred early in the solar system, as evinced by independent studies (see subsection below). Indeed, an early instability likely occurred if Jupiter, Saturn, and the other icy giants experienced such near-MMR states shortly after their formation. Second, the giant-planet formation was probably not a smooth process, and Mars-Earth-mass bodies or additional planets were present in the outer solar system. Collisions, gravitational encounters or secular effects involving such objects can turn a resonant configuration into a less stable near-MMR state^25,59,60^. Indeed, based on our database of giant planets locked in MMR chains, we confirmed such behaviour after performing auxiliary simulations including the presence of planetary bodies and the resonant Jupiter–Saturn pair, as similarly done in past representative work^25^ (Supplementary Information 3.5). These results agree with ref^25^, which showed that this near-MMR state could arise after an initial 2:1 MMR Jupiter–Saturn pair suffers perturbations from such massive bodies. Furthermore, we found that the interactions of icy giants before the instability can lead to similar behaviour based on additional simulations considering Jupiter, Saturn, Uranus, Neptune and a fifth icy giant on mutually resonant orbits. Based on the auxiliary simulations, as illustrated by representative evolutions in Figures S5 and S6, our Jupiter–Saturn pairs exhibited moderately eccentric orbits, PS/PJ ~ 2 (with osculating variation) and chaotic orbital behaviour during our simulations’ near-2:1 MMR phase, regardless of the initial instantaneous PS/PJ. Thus, any initial PS/PJ value close to 2 would work in our scenario. Overall, Jupiter and Saturn acquired averaged *e* ~ 0.05–0.1 and evolved spontaneously in near-resonance with durations between a few to a few tens of Myr. Last, the tendency of exoplanets to exhibit orbital period ratios close to 3:2 and 2:1 MMRs suggests that near-MMR configurations are expected outcomes in planetary systems^61,62^. Therefore, we conclude that Jupiter and Saturn likely experienced near-resonant interactions in the 2:1 MMR on moderately eccentric orbits after gas dispersal (the starting time of our simulations).

### Jupiter–Saturn: instability timing and post evolution

Evidence shows that the giant planets suffered dynamical instabilities less than 10–100 Myr after their formation^22,23,56,63–65^. However, as instability can excessively affect the terrestrial planets^66–68^, it probably occurred in ~ 10 Myr or shorter timescales allowing Venus and Earth to acquire their current dynamically cold orbits via dynamical friction with remnant disk objects^69,70^. Here, we assumed that the Jupiter–Saturn pair interacted in the near 2:1 MMR configuration for a few Myr, after which the giant planets experienced instability. As the duration of instability is typically short^17,51,57,67^ (< 100-a few hundred kyr timescales) and the post-instability residual planetary migration plays a minor role in sculpting the primordial asteroid belt beyond 2 au (ref.^17^ and Supplementary Information 3.3 and 3.4), we placed Jupiter and Saturn on near-current orbits after the planets spent their initial stage of interactions in the near 2:1 MMR. This procedure has been used in studies of the long-term evolution of the asteroid belt^45,71^. It can avoid considering too many parameters related to the complex orbital evolution during the instability. In addition, from a terrestrial-planet formation standpoint, the details are probably not important when the disk is already mass depleted beyond ~ 1.5–2 au during the instability or the instability occurs early^6,9,33^. These conditions are consistent with our scenario. Nevertheless, the role of the instability (e.g., timing, weak vs strong, etc.) on the formation of the terrestrial planets is still poorly understood^31,72^, so more detailed studies are warranted. Finally, the dynamical effects of the instability on the primordial asteroid belt are discussed in Supplementary Information 2.4 and 5.8.

### Protoplanetary disks: fundamental properties

The terrestrial planets assembled by accretion of embryos and planetesimals within the protoplanetary disk^1–6,8–10,19,32,50,69,70^. In particular, the key factors determining the final terrestrial planets are the properties of the disk within 2 au, the fractions of disk mass represented by embryos or planetesimals and the orbits of the giant planets^3,6,19,20,50^. Here, we considered disks consisting of tens of embryos and several thousand planetesimals. Embryos concentrated from 0.35–0.5 to 1.15–1.5 au, while planetesimals were placed all the way to 3.5 au. At smaller distances, the disk typically contained more mass represented by embryos than by planetesimals. All disks started with a total mass equal to 1.9–2.1 ME within 1.2 au, usually needed to form Venus and Earth with their current masses. Planetesimals initially located at 2–3.5 au represented the primordial asteroid belt, the initial total mass of which varied between 0.5 ME and 2.2 ME to cover possible variations of a massive belt. There was no mass depletion initially in any region of the disk. Overall, it is worth noting that these initial conditions are consistent with the predictions of several models of embryo and planetesimal formation^26,32,34,50,70,73–76^. In this way, we kept the model simple by avoiding the problem of having to consider too many input parameters in embryo/planetesimal formation and gas dynamics (see also discussion in Supplementary Information 2.6 and 5.8a). A disk inner region probably played a fundamental role in forming Mercury^1,19,21,69^, so we included this component in most of our disk models. We varied the disk inner edge (0.3, 0.4, or 0.5 au) and the initial total mass in the inner region to understand Mercury’s formation better. Typically, the mass distribution in our disks resulted in a surface density that followed an increase from the disk inner edge until a distance threshold, then a decrease until the disk outer edge following a decay power law, as similarly modelled in the literature^1,4,6,9,35,77,78^. Our choice of a distance threshold at 0.8–0.9 au is supported by the findings that a narrow 0.7–1.0 au disk and disks with inner edges at 0.5 or 0.7 au generally produce Venus-like planets too close to the Sun^19,20^. We also tested mass concentrations within the disk core region, motivated by the need to explain the small Venus–Earth mutual distance. Such mass concentrations might result from planetesimal/embryo pile-up^11–14^, so it is important to test their influence on terrestrial planet formation. Finally, our disk models’ different surface density slopes/shapes reflect the need to satisfy the abovementioned constraints. All these variations in disk properties resulted in our distinct disk models (Fig. S1, Table S1). Embryos and planetesimals started initially on near circular and coplanar orbits (*e*_0_ < 0.01 and *i*_0_ < 0.3°). Thus, our primordial asteroids were dynamically cold at the start of the simulations.

### Protoplanetary disks: object compositions

Inspired by asteroid taxonomy, we considered three major groups by composition in the disk: S, C and D/P. We assumed that our disk was composed of a mixture of local S-asteroids and C-asteroids. Local asteroids may have originated in situ or elsewhere (see below). In our nominal model, we assigned composition flags to these asteroids according to their initial location in the disk. This procedure resulted in proportions of C-asteroids of 10–20% and 50% within the < 2 au and > 2 au regions of our disk, respectively (conversely, 90–80% and 50% of S-asteroids within the referred regions). We chose a threshold of 2 au to define the terrestrial and asteroid belt regions. We also tested several taxonomic proportions but found that the nominal model yielded the best results (Supplementary Information 5.8). Some scenarios support this mixing of relatively “dry” volatile-poor S- and “wet” volatile-rich C-asteroids in the disk. For example, these primordial C-asteroids could be objects implanted from the trans-Jovian region during giant-planet formation *before* instability^40,79^. This scenario predicts more significant contamination of implanted asteroids with increasing heliocentric distance beyond ~ 1 au. Another possibility is that the evolution of the water ice line at ~ 1–3 au during planetesimal formation contributed to the formation of C-asteroids beyond ~ 1–2 au (ref.^26,80–82^). Also, the high fraction of C types among asteroids at *a* < 2.5 au supports the idea that C-asteroids were present in the inner regions of the primordial asteroid belt^83^. Therefore, these pieces of evidence justify the above choices of an initial contribution of C-asteroids among the local population in the disk. The S-asteroids presumably formed in situ by the end of gas dispersal^16,32,40^. Primordial D/P-asteroids were likely captured from trans-Jovian reservoirs *during* the instability and migration of the giant planets^43^. Ref.^84^ found that primordial Hilda asteroids and Jupiter Trojans were lost after the instability, so the currently known populations should consist of captured objects. As these populations consist of comparable C and D/P types, we considered that the captured asteroids were represented by 50% C- and 50% D/P-asteroids in our nominal model. In this way, the primordial asteroid belt was contaminated by both C- and D/P-asteroids by the time the giant planets acquired their current orbits. This contamination event is supported by dynamical modelling, spectral observations of asteroids and the presence of peculiar asteroids^43,85^.

Concerning the chondritic compositions in the disk, we assumed that objects rich in enstatite and ordinary chondrites (EC and OC) were represented by S-asteroids and that the concentration of OCs increased for more distant asteroids. In addition, carbonaceous chondrite (CC)-rich objects were represented by C- and D/P-asteroids.

### Protoplanetary disks: water mass fractions (WMFs)

We assigned distinct WMFs for the objects according to their initial locations in the disk to determine the amount of bulk water acquired by our terrestrial planets. Our goal was to constrain the water mass distribution in the disk by later identifying successful systems that satisfied the terrestrial planets’ water constraint (Supplementary Information 1). In particular, we considered a wide range of WMFs for each investigated region at < 1.5 au, 1.5–2 au, 2–2.5 au and > 2.5 au: 0.001–0.01%, 0.001–0.5%, 0.1–10% and 5–40%, respectively. A total of 80 WMF models were investigated (Table S2). This exploration also allowed us to understand better whether these specific regions were water-poor or water-rich at the onset of terrestrial planet formation. Finally, these WMF distributions are consistent with models for the origin of water in the inner solar system^3,40,82,86^ and the scenarios discussed in the previous subsection.

### Main simulations

We performed 650 N-body simulations of terrestrial planet formation to investigate the disk models described above. We used an optimised version of the MERCURY integrator^87^ to execute the simulations, including treatment of general relativity and calculation of the bulk density and size of the planets consistent with the terrestrial planets in the solar system^19^. The giant planets and embryos gravitationally perturbed one another. The planetesimals did not mutually perturb each other but gravitationally interacted with the planets and embryos. In these simulations, Jupiter and Saturn started with semimajor axes of *a* = 5.5 au and ~ 8.7 au and eccentricities of *e* = 0.08 and 0.1, respectively. An initial inclination of *i* = 0.5° was assumed for both planets. For each disk model, we placed Jupiter–Saturn in 60 distinct configurations near their mutual 2:1 MMR according to the orbital period ratios PS/PJ = 1.97, 1.98 and 1.99 (20 configurations per PS/PJ). We uniformly varied Saturn’s initial mean anomaly within 30–120°. All other angular elements were initially set to zero for Jupiter and Saturn. The initial resonant angles were 60–240°, but they quickly evolved to ~ 300–360° during the simulations. Here, our systems typically experienced the near 2:1 MMR Jupiter–Saturn for 5–10 Myr (20 Myr in a few cases), after which we took the orbital states of the embryos and planetesimals as the initial conditions for the next stage of the simulations with the giant planets placed on their near-current orbits. As expected after instability, we set the eccentricities slightly above the current values for Jupiter and Saturn. This procedure also allowed the eccentricities to damp slightly to current levels via dynamical friction with remaining embryos/planetesimals. Then, we evolved 50–100 systems per disk model until the total time reached 400 Myr, representing the system’s post-instability evolution. Overall, the PS/PJ ratio remained slightly below the observed 2.49, so the influence of the near 5:2 MMR was negligible. Additional auxiliary simulations are described in Supplementary Information 3.

To investigate water delivery to the terrestrial planets, we considered 87 systems that contained planets with optimal global properties (i.e., the median orbits and masses over all analogues of each planet were within 10% and 25% of the respective actual values) at the end of 400 Myr based on disk models A, B and C, which together represented the ‘standard disk’. As the main results were similar among all disk models tested in this study, the selected systems should be good representatives of the inner solar system (Fig. 3).

We did not include fragmentation in our simulations, but this was an acceptable assumption. First, simulations of the terrestrial-planet formation including fragmentation yielded system outcomes similar to those that did not^10,33,50,88–90^ because the generated fragments can be re-accreted by the forming planets. Also, it is unclear if fragmentation plays an important role or not in explaining the current orbit and mass of Mercury^91^. Furthermore, adding more confusion to the picture, the simulations that implemented fragmentation yielded more massive Mars analogues, thus decreasing the success of Mars formation^33^. The stochastic nature of planet formation, the different techniques/codes used to implement fragmentation in simulations, and the uncertainties regarding the fragmentation of primordial solar system objects (e.g., critical impact energy per unit mass Q_*_) make it difficult to judge and compare the results of past studies regarding fragmentation. Briefly, the role of fragmentation in terrestrial-planet formation remains a matter of debate, and further detailed studies are warranted. Nevertheless, a common observation in studies that included fragmentation is that dynamical friction was enhanced, which could lead to dynamically colder final planets^33,89^. Nevertheless, dynamical friction operated in our simulations because we used a large number of planetesimals that decayed over tens of Myr.

The simulation time step was 4.565625 days for disks A, B, C, D, E and 3.6525 days for disks Ia–e to ensure reliable calculations for planets with orbits similar to Mercury. Objects that evolved to heliocentric distances < 0.15 au or > 20 au were discarded from the simulations. This procedure did not influence our main results because these extreme objects can collide with the Sun or be gravitationally ejected by Jupiter on very short timescales.

### Asteroid belt formation: model, simulations and observations

We investigated the origin and evolution of the asteroid belt by considering a representative asteroid belt obtained in additional long-term simulations. Residual migration was not considered in this investigation, as justified in subsection “Jupiter–Saturn: instability timing and post evolution” above and in Supplementary Information 3.3. Due to computational constraints, we limited this investigation to remnant asteroid belts obtained in the systems of the standard disk, which initially contained a primordial asteroid belt, as described above. First, we selected 202 systems containing three or four terrestrial planet analogues after 100 Myr of post-instability evolution from the main simulations described previously (100 Myr was the closure time of planet formation in this investigation; henceforth ‘t100’). Thus, only systems that formed good representatives of the inner solar system were considered in this investigation. We further selected 46 systems with asteroid belts depleted at levels < 5% in which > 50% of the local asteroids acquired *i* > 10°. This procedure identified asteroid belts that experienced substantial depletion and *e*-*i* excitation simultaneously. Then, we obtained the orbital states of local asteroids remaining in those systems at t100. Next, we considered asteroids captured in the asteroid belt from trans-Jovian orbits based on data obtained in giant-planet migration simulations^44^. These captured asteroids acquired their final orbits within ~ 4 au after interacting with Jovian mean-motion and secular resonances^43,44^. Notably, the orbital structure of the captured asteroids and their capture efficiency in distinct regions of the asteroid belt were quite similar to those found by a distinct model that included the instability^43^, so the results of that study^44^ provide an acceptable representation of such a captured population at the end of giant planet instability/migration. We then obtained the orbital states of our captured asteroids after evolving them to t100. In the last stage, we combined the local and captured asteroids obtained at t100 and evolved them for a further 4 Gyr under the gravitational influence of the eight solar-system planets and the four most massive asteroids (Ceres, Vesta, Pallas and Hygiea). In this investigation, assuming that the planets were fully formed and placed on their current orbits allowed us to investigate the long-term evolution of the asteroid belt accurately because the associated mean motion and secular resonances in the inner solar system were correct. We found that 106 local and 658 captured asteroids survived within 2–3.5 au at the end of this stage (out of ~ 78 k local and 2118 captured asteroids, respectively, where the latter objects were captured out of 3 million trans-Jovian objects^44^). Finally, by random sampling of the captured population, we built representative asteroid belts based on relative contributions of 33%:67%, 50%:50%, and 67%:33% for local and captured asteroids, respectively (Figs. 4, S7 and S8). We also tested 80%:20% and 20%:80% proportions and found that in both cases, the resulting representative asteroid belts showed strong peaks in the semimajor axis that were inconsistent with actual observations. Therefore, the contribution of local and captured asteroids in our adopted representative asteroid was limited to a factor of 2 (i.e., comparable populations).

To compare our asteroids with observations, we selected 895 large asteroids with absolute magnitudes of H < 11 (> 20–30 km for assumed albedos 0.1–0.2) and not belonging to any asteroid family on 7 April 2021 from the AstDyS database. These asteroids are large enough that long-term collisional and non-gravitational effects would not affect their orbits^6^. In addition, the discovery of asteroids with H < 11 is essentially complete^92^. The compositional taxonomy of S-, C-, and D/P-type asteroids is based on the Bus-DeMeo classification^15,93^.

### Terrestrial planet system classification

It is crucial to identify the analogues of Mercury, Venus, Earth, and Mars in systems obtained in terrestrial-planet-formation studies^19,20^. This procedure also allows us to properly identify analogue systems that contain three or more planet analogues. As discussed in detail in ref.^19^, using a proper classification scheme can also mitigate the problem of incomplete and misclassification, thus reducing the chances of reaching misleading conclusions. This study used ref.^19^’s rigorous classification algorithm to identify all planet analogues formed in a given system. The algorithm obeys the following main steps: typically, the two most massive planets are identified as the Venus and Earth analogues of the system; next, the Mercury and Mars analogues are identified as the planets formed adjacent interior and exterior to the Venus and Earth analogues, respectively; finally, systems not analogous to the inner solar system are discarded (e.g., a system with a massive planet [> 0.32 ME] exterior to the Mars analogue). The planetary mass ranges considered for analogue candidates were 0.025–0.27 (Mercury), 0.4–1.5 (Venus), 0.5–1.5 (Earth) and 0.05–0.32 ME (Mars). Several past studies used similar mass ranges^9,13,21,94^. The upper limit for Mercury is slightly larger than usual to allow the possibility of mass-loss via erosive cratering^95^ or hit-and-run collisions^91^, which are not modelled here (see also Supplementary Information 5.2). Nevertheless, the influence of minor changes in mass ranges is unimportant compared to other model parameters, such as disk properties and the giant planet orbits^20^. A planet was defined as any object with a mass m ≥ 0.025 ME (a minimum of 50% of the mass of Mercury). Our classification algorithm required our terrestrial planet systems to contain planets analogous to the Venus–Earth pair and Mercury or/and Mars in the same system. In total, we identified 221 terrestrial systems that satisfied the above conditions. Then, we examined the properties of these analogue systems and the planets formed therein against fundamental constraints in the inner solar system (Supplementary Information 1). The numerical results discussed in this article are medians or ranges of medians obtained from our combined disk models (unless expressly stated otherwise): standard disk (ABC), representative disk with small inner regions (A–E) and representative disk with extended inner regions (Ix) (Tables S1, S4, S5). Finally, we note that the influence of our system/planet classification details on our results was unimportant. In general, the choice of Earth analogue mass classification (e.g., using a stricter 0.8–1.2 ME), system type (3-P or 4-P) and disk sample (individual or combined group) had little influence on the main properties of our terrestrial systems and their planets. Furthermore, the main results related to our planet were also insensitive to the classification details of our Earth analogues.

## Supplementary Information

Supplementary Information.

## Data Availability

The main findings of this study are supported by the data presented and the Supplementary Information. Additional data can be obtained from the corresponding author on reasonable request. The asteroid orbital data are available from the AstDyS database at https://newton.spacedys.com/astdys/. The asteroid taxonomy data based on the Bus-DeMeo classification system are available at http://www.mit.edu/~fdemeo/publications/alluniq_adr4.dat, https://sbn.psi.edu/pds/resource/taxonomy.html and https://sbn.psi.edu/pds/resource/busdemeotax.html.

## Code availability

MERCURY is publicly available at http://ascl.net/1201.008. The optimised MERCURY used in this study is based on the tweaked version of the code available at https://gemelli.spacescience.org/~hahnjm/software.html.
