# Supplementary material for: Terrestrial planet and asteroid belt formation by Jupiter–Saturn chaotic excitation
Source: Sci Rep. 2023 Mar 27;13:4708. doi: 10.1038/s41598-023-30382-9 (PMC10042868; doi:10.1038/s41598-023-30382-9)
Supplement: Supplementary file 1 — Supplementary Information. [file 41598_2023_30382_MOESM1_ESM.docx]

# Supplementary Information

1. INNER SOLAR SYSTEM CONSTRAINTS AND SUCCESS CRITERIA

A successful model for the inner solar system should satisfy all the constraints described below.

*Formation of Mercury, Venus, Earth and Mars analogues.* Reproducing the orbits and masses of the four terrestrial planets simultaneously remains elusive^1,2,4,5,6,13,19,20,21,32,33,69,70,94^. Given this difficulty, an analogue system has to form a minimum of three planet analogues, including the Venus–Earth pair. In addition, while the orbits of Venus and Earth are dynamically cold (*e* ~ 0.03 and *i* ~ 2°), Mars has a mild orbit (*e* ~ 0.07; *i* ~ 4°). Finally, Mercury’s excited orbit at *a* ~ 0.4 au (*e* ~ 0.2; *i* ~ 7°) and its low mass are difficult constraints to satisfy among the terrestrial planets^19,21,96^.

*Formation timescales of the terrestrial planets.* Earth’s formation timescale is often assumed to be that of the formation of the Moon, but the latter is not well constrained (see below). Isotopic constraints indicate that 80% of Earth’s mass most likely accreted within a timescale of 10–35 Myr, while the remaining mass accreted over the next tens of Myr^42^. Venus probably experienced a similar accretion history. The bulk of Mars’ mass (>80–90% of the final mass) could have accreted in less than 10 Myr according to Hf-W-Th chronometry^97^. However, the same isotopic data is also consistent with longer accretion timescales if Mars formed protractedly involving giant impacts^98^. Indeed, some studies supporting the latter idea found Mars’ formation timescales of ~15–20 Myr (ref.^99,100,101^) or even ~60 Myr (ref.^102^). Here, consistent with these estimates, a timescale of ≤ 15 Myr was assumed for Mars formation. Timescales up to 50% longer (~23 Myr) were acceptable, provided that ≥ 80% of the mass of Mars was accreted within 5 Myr. Lastly, to our knowledge, Mercury’s formation timescale is unconstrained.

*Formation of the Moon.* Estimates of when the Earth experienced the giant impact that formed the Moon varied within *t* = 25–245 Myr in the time framework of our model^36,37,103,104,105,106,107^. This wide range of timescales highlights that Moon formation is still a matter of debate, which includes a variety of plausible impact geometries, thermal states, compositions, and masses of the impactor and the target^106,107,108,109,110,111,112,113,114,115,116^. While it is typically assumed that a ~Mars-mass object caused this impact^108^, qualified Moon-to-Mars mass impactors are as capable of forming the Moon^117^, which is supported by Moon-formation models^111,112^. Geochemical constraints limit the Moon-forming impactor to at most 0.1–0.15 ME and favour less massive impactors^118^. An independent analysis concluded that one or a few impactors with masses within 0.01–0.1 ME formed the Moon^119^. Here, we considered these uncertainties and assumed that impactors with > 0.02, > 0.03, > 0.05 or > 0.10 fractions of the target’s mass were representative of Moon-forming giant impacts.

*Late veneer mass delivered to Earth.* The fraction of mass delivered to the Earth’s mantle via impacts of remnant objects during late terrestrial-planet formation was within 1% of Earth’s mass^47,48,117^. In addition, most of the objects that contributed to the late veneer were probably rich in dry enstatite chondrites^41,120,121^. Finally, more massive impactors (≥ 0.001 ME) likely deposited only a fraction of their masses into Earth’s mantle. In particular, for 0.01- and 0.03-ME impactors, the contributions were only 10–30% and 10–20%, respectively^122^. This effect was considered in our simulations by considering the contribution upper limits conservatively.

*Origin of water on Earth and the other terrestrial planets.* Earth’s bulk water was likely delivered to our planet by impacts of various water-bearing objects present in the protoplanetary disk during its formation^3,4,19,38,39,40,41,123^. Estimates of the mass of water on Earth vary significantly^41,103,124,125^. Here, Earth analogues were required to acquire water mass fractions (WMFs) in the range 5–25 × 10^−4^, or 2–10 times Earth’s current water in the oceans. The WMFs of Venus and Mars are much less constrained. Here, we assumed a minimum WMF of 1 × 10^−5^ and a maximum WMF of 5 × 10^−4^ (‘dry Venus’) or 5 × 10^−3^ (‘wet Venus’) for Venus. For Mars, we assumed that a range of WMFs within 0.5–20 × 10^−4^ was required^41,126,127,128,129^. We considered a WMF model successful if the three median WMFs obtained for the analogues of Venus, Earth, and Mars simultaneously satisfied the estimated WMF intervals for each of the planets (Table S2). Finally, 5–30% of Earth’s water was likely delivered after the Moon-forming giant impact, so the bulk of the Earth’s water was accreted before this event^41^.

*Terrestrial planet system properties.* The angular momentum deficit (AMD) measures the dynamical excitation of a system via the eccentricities and inclinations of planets formed therein. At the same time, the radial mass concentration (RMC) evaluates the mass distribution of the planets in terms of their orbits (see ref.^1^ for more details). Here, consistent with representative studies^4,6,9^, the final systems should possess a maximum of 200% of the AMD and 50–200% of the RMC of the terrestrial planets.

*Absence of planets in the asteroid belt.* Whether planets can form locally or move in from other regions, they must not acquire stable orbits in the asteroid belt after terrestrial-planet formation; otherwise, peculiar orbital features that do not match observations arise in the belt^130^.

*Orbital architecture, compositional taxonomy and the low mass of the asteroid belt.* A successful asteroid belt model should simultaneously explain the orbital/compositional structure and the low mass of the asteroid belt at 2–3.5 au (Figure 4). Our sample of observed asteroids is a good representative of the asteroid belt (Methods) and exhibit *a-e-i* distributions consistent with independent studies of the belt^131,132^. In particular, an essential constraint regarding inclinations is the ratio of asteroids with inclinations above the location of the nu6 secular resonance in *a*-*i* space, which is currently f6 = 0.07 at a < 2.82 au. About taxonomy, asteroids are often classified as S- and C-type asteroids, which are assumed to be poor and rich in water (or volatiles), respectively^123^. However, studies of surface/geochemical processes and spectral taxonomy indicate that while C- and D/P-type asteroids would be water-rich, they likely belong to distinct groups^15,93,133^. Finally, while S- and D/P-type asteroids are concentrated in the inner-middle and outer regions of the belt, respectively, C-asteroids seem to be broadly present throughout the entire belt. Another fundamental constraint is the total mass of the asteroid belt, estimated at 5 × 10^−4^ ME (ref.^15^).

2. CHALLENGES IN MODELS OF TERRESTRIAL-PLANET FORMATION: A CRITICAL OVERVIEW

*2.1 Classification of terrestrial planets and planetary systems*. The lack of proper identification of planet/system analogues and oversimplistic classification schemes are overlooked in terrestrial-planet-formation studies^19^. For instance, many representative models discussed below show the planets mixed without discriminating planet analogues or individual planets without clarifying if they belong to analogue systems. We believe that analysing individual planets (e.g., constraints related to Earth) and terrestrial planet systems should be based on adequately classified systems containing three or four analogues of the terrestrial planets. However, only a few recent models have considered the importance of classifying analogue systems in their analysis^6,9^.

*2.2 Is the narrow disk 0.7–1.0 au an adequate baseline?* The outcomes of narrow protoplanetary disks with mass concentrated at 0.7–1.0 au (ref.^7,10^) (henceforth ‘canonical annulus’) have served as the basis for several Mars formation models: Grand Tack^8,117,124,134^, early giant-planet instability^6,9^, empty asteroid belt^18^, pebble accretion^32,135^ and sweeping secular resonance^136^. In particular, the Grand Tack and early instability models have explored the Mars formation problem in detail. Although narrow disks obtained by these models appear to solve the Mars small-mass problem, there has been insufficient discussion about other outstanding problems. To cite a few, simultaneously forming Mercury (in addition to Venus/Earth or Mars in a given system), explaining the cold and close-in orbits of the Venus–Earth pair, reproducing the moderately excited orbit of Mars and its mutual distance with Earth, and confronting other fundamental constraints (Section 1) in systems that contain three or more planet analogues with the observations in those models. More importantly, detailed investigations of three- or four-planet analogue systems properly identified in such disks indicate that they have difficulties reproducing the terrestrial planets and satisfying other constraints in the inner solar system^19,20^.

First, after applying the same classification system used here to the best representative canonical annulus model (50 systems of *D1* disk modelled in ref.^20^), we found that the formation of Mercury analogues and systems simultaneously containing analogues of the four terrestrial planets (henceforth ‘4-P systems’) were inefficient at 6% and 4%, respectively. In contrast, here (disk Ix), these efficiencies reached 34% and 13%, respectively. The problem of forming Mercury in tandem with the other terrestrial planets is a well-known issue (Section 2.3). Furthermore, for several types of similar narrow disks (e.g., akin to Grand Tack) tested in our previous work^19,20^ (e.g., *T7–10* and *T7–12* narrow disks with core mass at 0.7–1.0(1.2) au + low-mass components beyond 1.0(1.2) au and *D1* narrow disks) the analogues of Mercury formed too far from the Sun typically at semimajor axis *a* > 0.52 au and were located too close to the Venus analogues. Indeed, using the same success criterion 0.67–1.33 × dMV (Mercury–Venus mutual distance) as used here, we found 0% success for *D1* (ref.^20^), *T7–10* and *T7–12* disks^19^. In contrast, we obtained 30% in our scenario (Section 5.2). Similarly, the analogues of Mars formed too close to the Earth analogues in *T7–10* and *T7–12* disks (0.32 and 0.39 au, respectively). This problem was not observed in our results. Furthermore, analysis of planet analogues in orbital-mass space revealed the failure of the canonical annulus model to reproduce simultaneously the orbits and masses of the Venus–Earth pair^20^ (that work’s figure 2 supports this conclusion). Another issue is that concentrating disk mass within a 0.7–1.0 au annulus yielded a low probability of forming Venus and Earth within the same boundaries. Because the disk expands due to self-stirring^6^, Venus and Earth tend to form too far from each other. Despite differences in model details or classification schemes, representative studies^6,9,31,33,134^ of the above scenarios reveal similar trends regarding Venus/Earth. In addition, the Earth analogues generally acquired 90% of their final masses too fast: ~7-16 Myr (medians), consistent with the Grand Tack’s results. Consequently, these planet analogues experienced Moon-forming giant impacts too early, with success fractions of 0%, 13% and 33% for *T7–10*, *T7–12* and *D1* disks, respectively (For an ITR > 0.05), and accreted too much late veneer mass after these impacts in agreement with the trends found in ref.^117,124,134,137^.

Finally, the summaries of formed planets as illustrated in plots of orbit vs mass reveal potential systematic issues in the studies mentioned above: 1. Overly noisy or peculiar distributions of planetary orbits/masses (e.g., Venus-like planets concentrated at ~0.5 au); 2. Recurrent presence of undermassed Venus-/Earth-like planets or overmassed Mercury-/Mars-like planets; 3. Lack of identification of individual planet analogues. These points contrast with our improved results regarding orbit-mass distributions of the planet analogues (Figure 3).

*2.3 Forming Mercury and 4-P systems.* The formation of Mercury is an outstanding problem that has been largely neglected in terrestrial-planet-formation studies^2,3,4,5,8,10,12,13,27,32,33,35,50,69,70,77,78,88,90,94,136,138,139,140,141,142^ (see also discussion in ref.^19,20^). Other studies have not obtained satisfactory results^1,6,7,9,11,18,31,72,89,134,137,143^. Although recent dedicated models have explored different scenarios for Mercury’s formation in terms of its orbit, mass and internal structure^21,91,96,143,144^, there is still no consensus on the best model regarding the reproduction of Mercury’s main properties. More detailed studies are warranted to investigate whether any of these scenarios could form 4-P systems and satisfy other constraints in the inner solar system.

Because distinct classification schemes were employed and other details were lacking in past work, a direct comparison with our results is problematic. Nevertheless, we briefly discuss a few results below for completeness. We limit the discussion to the terrestrial planet formation models that considered Mercury formation in their terrestrial systems. First, Mercury-like planets were obtained in ≤1% of the systems in the Grand Tack^134,137^ and the early instability models^9,33^. More recent investigations of the early instability model yielded ~4-6% and <1% probability of producing Mercury-like planets in Mercury–Venus systems^31,91^ and 4-P systems^91^, respectively. Despite the above caveats, these values are lower than those obtained in our best disk models: 34% and 13% for Mercury analogues (in Mercury–Venus–Earth and 4-P analogue systems combined) and 4-P systems, respectively.

*2.4 The influence of Jupiter–Saturn evolution during the instability.* The early instability model considered instabilities valid for orbital period ratio PS/PJ as large as 2.8 in their first series of studies^9,16,33^. However, because secular resonances associated with Jupiter and Saturn can strongly perturb the asteroid belt, it is unclear if instability evolutions characterized by PS/PJ ~ 2.5 (interacting with the 5:2 MMR) or crossing of the 5:2 MMR (for PS/PJ = 2.5-2.8) perturbed the belt unrealistically. Ref.^6^ considered three individual instability evolutions characterized by PS/PJ ~ 2.3, ~2.45, and ~2.45-2.55. The latter case was also used as the basis for a representative asteroid belt formation model^17^. However, this particular instability evolution raises concerns about the influence of the 5:2 MMR. Our results suggest the intriguing possibility that a similar Jupiter–Saturn chaotic excitation (JSCE) mechanism based on the near-5:2 MMR played a role in perturbing the asteroid belt in that study. On the other hand, even if this were the case, one would need to explain how Jupiter and Saturn experienced PS/PJ ~ 2.5 and then later acquired their current smaller PS/PJ (~2.49) that is not experiencing JSCE. Furthermore, the terrestrial systems obtained under this specific instability evolution are also problematic: the planets seem dynamically too excited, and Mars-like planets are scarce^6^. Our scenario does not suffer from the shortcomings mentioned above. We also confirmed that the influence of the instability was modest in our investigations, so our main results stand (Methods and Section 3).

*2.5 Explaining the orbital distributions and compositional taxonomy of asteroids.* Asteroids obtained in Grand Tack^8,134^, early instability^6,9,16,33^ and asteroid belt formation models^17,45,145^ are typically overexcited in inclinations compared to observations. This issue is closely related to the longstanding problem of reproducing f6 (Section 1), which remains unexplained by these models. In fact, models that consider the Grand Tack’s results at gas dispersal as their initial conditions recognise the overexcitation problem, so they assume instead that the asteroids concentrated at *i* < 20° before the instability^6,45,145^. Although the final phase of Jupiter–Saturn residual migration could reduce f6 by 60% on average, the resulting f6 values were eight times the observed one in the early instability scenario^146^. Other issues reported in some of those studies are the deficit of low-*e*/low-*i* asteroids^17,45^ and overexcitation of eccentricities^84^, which are also seen in asteroid belts^6^ obtained for the instability evolution used in ref.^17^. The most promising asteroid belts obtained in ref.^6^ are based on the instability evolution characterised by PS/PJ ~2.45. However, in this particular case, the terrestrial systems appear to possess planets too dynamically excited, overly massive Mars-like planets, and Venus-like planets concentrated at ~0.5-0.6 au. In contrast, in the framework of the JSCE scenario, our asteroid belt acquired a less excited orbital structure more compatible with observations. Another representative scenario is the empty primordial asteroid belt model^18^. However, the lack of details in that study precludes a more in-depth comparison of model results with the various properties of the asteroid belt (Section 1) or discussion about the influence of the giant planets’ orbital evolution. Contrary to the long-term investigations in our study, the obtained asteroid belts in the early instability and empty asteroid belt were not evolved to the age of the solar system (e.g., ~4 Gyr), making a comparison with observations less straightforward. Finally, the compositional structure of the asteroids is seldom addressed with detailed statistics. The Grand Tack model considered S- and “C-type” populations (this population mixed C- and D/P-type objects), but their distributions differ from the current observed one (Figure 4b). Similarly, the early instability model found their S- and C-type populations concentrated in the inner and outer regions of the asteroid belt. However, these results were contaminated by systems that experienced too violent giant planet instabilities. Here, we argue that analysing three main populations (S-, C-, and D/P-types) is more comprehensive and can lead to a better understanding of asteroid belt origin and evolution (Methods and Section 5.8).

*2.6 Early formation of terrestrial planet systems.* Recent planetesimal/embryo-formation models have reported forming terrestrial planets^11,13,27^. Although these models have yet to demonstrate that their results can satisfy the various constraints of the inner solar system, we discuss them here for completeness. First, these models deal with a large number of parameters related to gas dynamics, pebble accretion or planetesimal/embryo formation. For this reason, the parameters are explored preferentially to produce disk mass concentration near Venus–Earth’s current orbits or “empty” asteroid belts to prevent overmassed Mars-like planets from forming. Second, the formation and dynamical evolution of Jupiter–Saturn played an essential role during the disk gas phase, but the giant planets were not included in representative models (1) and (2) discussed below. We now discuss three representative models.

(1) The strength of the embryo-convergence model^11^ is the tendency of Venus- and Earth-like planets to form mutually close within the convergence region. However, this orbital convergence is also a problem because Mercury- and Mars-like planets tend to form too close to their Venus and Earth counterparts. Regarding Moon formation, most of the obtained Moon-forming giant impacts occurred too early, with a median probably falling within 10-20 Myr after gas dispersal. Despite the low likelihood of late impacts, it is assumed that a 5-planet system experienced one to match the Moon-formation constraint, thus resulting in a 4-planet system. This hypothesis requires that the 5-planet system was dynamically unstable at some point after its formation. However, the orbital excitation experienced by the planets during this late terrestrial planet instability was not discussed. The eccentricities of the Venus/Earth-like planets were generally ~0.015-0.02 before this event, so both the terrestrial and the giant planet instabilities are likely to overexcite the orbits of Venus and Earth^66,67,147,148^. It is also difficult to evaluate the level of success of the individual planets or systems formed because the planets are shown mixed, and the classification of analogue systems is unclear. Finally, the information about the asteroid belt is insufficient to allow an adequate comparison with observations.

(2) The pebble accretion model^27^ relies on two key assumptions: i) formation of the building blocks (planetesimals) of Venus, a 0.6 ME-Earth, Theia (the Moon-forming impactor), and Mars at the ice line location fixed at 1.6 au; ii) a 0.4 ME-Theia located at ~1.2-1.5 au after gas dispersal. However, the ice line likely wandered around 1-3 au during planetesimal formation^26,80,81,149^. Thus, this would lead to various possible growth tracks decreasing the likelihood of the conditions described in i-ii) above. Also, the likelihood of Theia colliding with Earth late enough and the level of orbital excitation acquired by Venus, Earth, and Mars after terrestrial *and* giant planet instabilities are unclear. Furthermore, Mercury’s formation is difficult to evaluate, as the planet was not explicitly modelled in this scenario. Finally, a model of asteroid belt formation was not presented.

(3) The planetesimal-ring model^13^ claims that the terrestrial planets formed from a disk containing ~2.5 ME of mass concentrated at ~0.7-1.5 au. Such disks are wider than the 0.7-1.0 au narrow disks discussed above, resulting in worse final terrestrial-planet systems in orbit-mass space. In particular, this is evinced by the formation of Venus-/Earth-like planets covering a similarly wide range at *a* ~ 0.5-1.5 au and the existence of individual systems containing three or more such massive planets. Another piece of evidence is the presence of overmassed Mars-like planets. Thus, the chances of obtaining low-mass Mars-like planets are more diminutive in spatially wider disks. Mercury formation was also neglected. Furthermore, there are some unclear points about the classification scheme. Which planets were explicitly classified as the individual Venus, Earth and Mars analogues? How additional Venus-/Earth- or Mars-like planets were classified in specific systems? Can a system with three or more Venus-/Earth-like planets be considered a good representative of the real one? Finally, there is an insufficient discussion about the formation of the asteroid belt, so it remains to be shown if the belt’s orbital structure, compositional distributions, and low mass can be explained consistently in that scenario.

Concluding, any successful model for the inner solar system should reproduce the four terrestrial planets and the main properties of the asteroid belt in a single scenario. It is also clear that the initial conditions of the disk from which the inner solar system originated must be better constrained. Despite the limitations of our model, we believe that it promotes advancement in our understanding of terrestrial and asteroid belt formation.

3. AUXILIARY SIMULATIONS AND RESULTS

*3.1 Control systems.* We simulated 70 control systems that considered Jupiter and Saturn on their current orbits from the start using disks A, B, C, D and E (Table S1). A median of 35% of asteroids survived in these systems after 400 Myr of evolution. Furthermore, as there was no effective mechanism to excite the inclinations, all the asteroids still had low inclinations (< 5°) at the end of the simulations. In addition, even the best control systems formed Mars analogues systematically more massive than Mars, with orbits too close to Earth analogues and on timescales longer than 25 Myr. Therefore, a system starting with the current giant planets cannot explain the formation of Mars and the asteroid belt for a massive disk extending beyond 2 au. This result strengthens the case that either the disk was dynamically truncated early or the primordial region beyond ~1 au was never high-mass.

*3.2 Jupiter–Saturn 2:1 MMR systems.* We also simulated 120 systems containing Jupiter and Saturn deeply locked in a 2:1 MMR (i.e., with small resonant angle amplitudes) using disks A, B, D and E. We found no dynamical excitation beyond ~1–1.5 au in such systems over timescales of 30–40 Myr. Consequently, the terrestrial-planet-forming region at < 2 au and the asteroid belt (> 2 au) often remained massive in these systems. As such disk conditions cannot form Mars and the asteroid belt, and late instabilities are currently disfavoured (Methods), these results imply that Jupiter and Saturn did not experience long-term deep locking in the 2:1 MMR during terrestrial-planet formation if an extended massive disk existed beyond ~1 au after gas dispersal. Instead, the success of our scenario based on JSCE, the evidence favouring an early instability and the existence of ~Mars-Earth-mass bodies during giant-planet formation indicate that Jupiter and Saturn experienced a near-resonance interaction in the 2:1 MMR.

*3.3 Jupiter–Saturn residual migration.* To test the effects of residual migration, we considered systems in which Jupiter and Saturn started on orbits such that their orbital period ratio PS/PJ was initially in the 2.3–2.45 range. These orbital configurations were set to mimic the orbital states of the giant planets acquired immediately after their instability. We also placed the four terrestrial planets on dynamically cold orbits (*e_0_* = 0.001 and *i_0_* = 0.1°) at their current semimajor axes. Then, following fictitious forces as implemented in the MERCURY code, Jupiter and Saturn smoothly migrated to their current orbits following an exponential behaviour with exponent (-t/tau); two values of tau were tested: 1 and 10 Myr. A total integration time of five times tau was used, resulting in 5- and 50-Myr timescales for the residual migration simulations described here. Overall, the results indicate that the orbit of Mercury could have been excited to eccentricities of *e* ~ 0.1–0.3 and inclinations of *i* ~ 7–10° during this residual migration. Therefore, in our scenario framework, after Mercury’s formation in the inner regions of the disk, Mercury’s current orbit could be explained by further excitation caused by the residual migration of Jupiter and Saturn. We also tested the effects of residual migration on hypothetical asteroids concentrated at 2.2-3.2 au and possessing a wide range of eccentricities (0-0.2) and inclinations (0-38°). The final orbital structures were similar to the initial one, suggesting that the effects of residual migration were subtle for such an asteroid belt. A more detailed investigation of residual migration in the inner solar system is left to future work.

*3.4 Giant planet instability*. First, we considered 5-giant planet systems containing fully formed Jupiter, Saturn, and three icy giants. By employing fictitious forces to mimic the gas-driven migration of giant planets during the early solar system (a common technique used in past studies^51,55,57^), the five giant planets acquired mutual resonant orbits described by 2:1, 4:3, 3:2, 3:2 MMRs. This multi-resonant configuration is one of the preferred in past studies^45,57^. After slightly varying the initial conditions for these simulations, we obtained 357 unique resonant systems. In the first stage of our instability simulations (inst-S1), consistent with our current understanding of the outer solar system^24^, we added a 20 ME dynamically cold trans-planetary disk at 20-30 au to each system (the disk contained 4000 massive objects). Then, by randomizing the mean anomaly of the icy giants to force early instabilities, we allowed the systems to evolve until the onset of the instability. Next, to increase the likelihood of obtaining “good” instability outcomes, we selected 49 post-instability systems that resulted in Jupiter, Saturn, and two icy giants and PS/PJ close to 2.5 within 20 Myr. Then, we inspected the orbital output of the selected systems and identified the approximate timing of imminent instability (*t*_inst_). In the next stage, we used the orbital state of the giant planets and disk objects of each system at *t*_inst_ as the initial conditions of new instability simulations (inst-S2). In addition, we also added 5271 asteroids beyond 2 au to each of these 49 seed systems. These asteroids were obtained at the end of JSCE based on the standard disk (Methods). To increase number statistics, we created additional 231 systems based on the seed systems by varying the mean anomaly of the asteroids, thus totalling 280 systems in inst-S2 simulations. These simulations evolved for 10 Myr. As expected, most of these systems experienced instabilities within a few Myr. Next, we selected post-instability systems of inst-S2 that experienced PS/PJ = 2.3-2.5 and Jupiter’s mean eccentricity larger than ~0.03 (sample A: 41 systems) or ~0.04 (sample B: 29 systems) during the instability (note: sample A contains sample B plus 12 systems). These optimal instability systems are roughly consistent with the best outcomes found in similar works^51,57^. The instability was deemed finished when PS/PJ was the closest to 2.49 for each system (*t*_end_). Conversely, when a system reached a time equals *t*_end_, we assumed that the giant planets acquired their final orbits. At this point, we replaced the giant planets with the real ones on their current orbits and considered the orbital state of the asteroids found at *t*_end_ as the initial conditions of long-term simulations for each system (4.1 Gyr, for consistency with our main simulations). A control system without instability consisting of the four giant planets and the 5271 asteroids was also evolved for 4.1 Gyr. Finally, we analyzed the orbital structure and dynamical depletion levels of the obtained asteroid belts in the control and the 41 optimal instability systems. Compared to the control system, we found that the depletion of asteroids increased by 2-78% (median 27%) for sample A, whilst we obtained 12-78% (median 34%) for sample B.

*3.5 Generating a near-2:1 MMR Jupiter–Saturn*. Similarly to the methods of ref.^25^, we added Mars-Earth mass bodies to our multiple giant-planet systems described above (Section 3.4). We assumed various initial orbits for these bodies according to the location of internal/external MMRs with Jupiter (*a*_0,J_ ~ 5.6 au) or Saturn (*a*_0,S_ ~ 8.9 au). This procedure typically resulted in *a*_0_ = 7.3 au, 10.3 au, and 10.8 au as the initial orbits of the planetary bodies. The masses tested for the planetary bodies were 0.1 ME, 0.2 ME, 0.5 ME, 1 ME, and 5 ME. No planetesimals were present in the disk. In the first batch of simulations, we considered solely the Jupiter–Saturn pair locked in 2:1 MMR and added a single planetary body. In the second batch of simulations, we considered our 5-giant planet multi-resonant system with and without an additional planetary body. Finally, our resonant Jupiter–Saturn pairs exhibited slightly different initial eccentricities across the various systems explored (~0.005 to 0.05). Considering that these simulations were highly stochastic (most experienced instabilities), we performed hundreds of simulations to probe some possible outcomes. Nevertheless, this investigation was not exhaustive, so dedicated studies are warranted to better understand the chaotic evolution of Jupiter and Saturn before the instability.

4. JUPITER–SATURN IN THE NEAR-2:1 MEAN MOTION RESONANCE

In a chaotic system, minimal differences in the initial conditions can yield distinct outcomes often described by exponential diverging behaviour^150^. For example, the orbital evolution of the planets in the solar system is chaotic, despite its long-term stability over 4.5 Gyr (ref.^151,152,153^). Here, although the initial conditions of Jupiter and Saturn were similar and both planets could remain stable during their near-resonant interactions in the 2:1 MMR over Myr, various frequencies associated with their orbital elements, such as the secular frequency associated with the longitude of the perihelion, wandered over wide ranges of values, clearly indicating chaotic behaviour (Figure S2). The orbital evolution experienced by our near-2:1 MMR Jupiter–Saturn pairs was similar to the findings of ref.^25^. Indeed, both planets exhibited similar libration/circulation of the two associated resonant arguments as defined in that work^25^. We think this chaotic behaviour arises when Jupiter and Saturn evolve with motion near the boundary of resonant motion and slow circulation in the 2:1 MMR (ref.^60^). Such an orbital configuration has also allowed the chaotic capture of Jovian Trojans during planetary migration^44,154^. Finally, although Jupiter and Saturn behave chaotically before the instability, the effects generated in the disk beyond ~1 au and the post-instability system outcomes were statistically similar among the various simulation runs.

An additional reason for our initial conditions is that the Jupiter–Saturn chaotic excitation (JSCE) mechanism seems much stronger when Jupiter and Saturn evolve near their 2:1 MMR rather than the 3:2 or other MMRs^25^. Therefore, as disk gas dynamics usually result in both planets experiencing the 3:2 or 2:1 MMR, our results suggest that Jupiter and Saturn evolved towards the latter. Perhaps the gas disk was of low mass to allow the planets to park near the 2:1 MMR instead of passing through it toward other internal MMRs, such as 3:2 (ref.^52,53,54^).

5. ADDITIONAL RESULTS AND IMPLICATIONS

*5.1 Global properties of terrestrial planet systems.* When analysing system properties, we found that the majority (60–80%) of our analogue systems satisfied the < 2AMD constraint depending on the disk model (Table S5). In particular, representative disk Ix yielded the highest successful fractions. Briefly, most of our systems remained dynamically cold by the end of the terrestrial-planet formation. This result is encouraging because including fragmentation or considering a higher number of planetesimals in the model would improve the system AMDs via enhanced dynamical friction^33,89^. Furthermore, approximately 85–95% of all systems satisfied the 0.5–2RMC constraint. Similar to previous models, our system RMCs were concentrated below the solar system value with medians RMC = 52–64. Nevertheless, these systems produced better RMC distributions than those found for systems containing the Venus–Earth pair in the early instability scenario^6^. However, because the RMC is highly sensitive to both the orbits and masses of Mercury and Mars, further improving RMCs will require detailed investigations of disk properties to increase the odds of producing optimal 4-P systems closely resembling the inner solar system. In fact, the AMD and RMC alone should not be used as primary success criteria for terrestrial system classification^72^. Instead, these metrics offer only complementary insights about the system’s properties. Finally, most of the mass in embryos likely concentrated within 1.5 au in the disk, which facilitated the formation of compact analogue systems. Indeed, no individual planets were found within the asteroid belt in our 221 analogue systems.

*5.2 Mercury formation.* Out of 122 Mercury analogues obtained in our model (Table S4), 47 were found in 4-P systems and 75 formed in 3-P systems, which simultaneously contain analogues of the Venus–Earth pair and Mercury or Mars. Approximately 75% of these systems contained either a Mars-like planet (4–5 times Mars mass on average) or one with too small a mass (< 0.5 times Mars mass). Therefore, most of these 3-P systems can be considered almost as good as 4-P systems. Overall, including extended inner regions in the disk (disk Ix) yielded systems with better Mercury–Venus orbital separations (real one *a*_V_-*a*_M_ = dMV = 0.34 au). Specifically, 30% of these Mercury–Venus pairs fell within the success range 0.67–1.33 × dMV (vs ~10% for the other disks combined). Similar results were obtained when considering the criterion 0.67–1.33 × orbital period ratio of both planets, PV/PM: 45% (vs 15%). However, these relatively low fractions indicate that model improvements are warranted. Despite the difficulty in satisfying this constraint^19,21,96,144^, these results suggest that an inner region component with slightly distinct boundaries and total masses could explain the observed separation of both planets.

Although our simulations produced individual analogue systems containing Mercury analogues with masses comparable to Mercury (~0.025–0.10 ME), our planet analogues’ median masses were generally about two to three times the mass of Mercury. First, we think further exploring the initial model conditions may reveal inner-region properties capable of producing more Mercury analogues with masses comparable to the real planet. In particular, confirming investigations reported in the literature^19,21,143^, we found that the mass distribution and total mass in the inner region seemed to play essential roles in determining the final mass of Mercury analogues. Second, including fragmentation in the simulations may also help produce less massive Mercury analogues. Combining these two factors could lead to successful results concerning Mercury’s small mass (i.e., median masses close to 0.055 ME).

Disk Ix successfully formed several Mercury analogues because the innermost embryo (protomercury) was typically located inside 0.5 au at the start of the simulations. Later, the analogues experienced modest variations in the semimajor axis due to collisions or gravitational scattering with other embryos within the inner region. Our Mercury analogues acquired their final masses by continuously accreting local and distant objects in the disk. Planetesimals dominated this accretion, so a substantial flux impacted the Mercury analogues during their formation. Among these planetesimals, a fraction possessed large eccentricities and inclinations that led to highly energetic collisions (e.g., Figure S4a,b). Because JSCE excited the disk beyond ~1-1.5 au in only a few Myr, presumably objects in the Mercury-forming region also experienced a similar bombardment of planetesimals. Therefore, these results support the hypothesis that Mercury’s local building blocks and the forming Mercury were enriched in iron by cratering erosion^95,155^ (i.e., resulting in Fe core fractions greater than the canonical 30%). It is also possible that other iron enrichment processes operated in the Mercury-forming region^21^. In conclusion, Mercury’s iron core could result from the accretion of iron-rich building blocks and surface/mantle erosion experienced by the planet during its formation. Alternatively, hydrodynamical simulations suggest mantle-stripping collisions with one or a few embryos could explain Mercury’s iron core^110,156^. Nevertheless, terrestrial-planet formation models testing this hypothesis could not explain Mercury’s core fraction (~75%) and orbit/mass consistently^21,91,96^. On the other hand, these models explored only a limited range of initial conditions.

In summary, a dedicated study considering our favoured protoplanetary disk, giant-planet conditions, and the inclusion of erosion processes or fragmentation in N-body simulations would be an exciting avenue to discriminate the best Mercury-formation scenario. In addition, such studies would also allow us to understand better why Mercury seems peculiar compared to the other terrestrial planets. However, as simulations involving Mercury formation are very computationally intensive, we leave these more detailed investigations for future work.

*5.3 Venus and Earth formation.* We obtained a large number of Venus–Earth pairs (221) belonging to 3-P/4-P systems obtained from all simulations in this study (Table S4). As discussed in the main text, disks with initial mass concentrations within the core region (disks B, C and Ix) more often matched the mutual distance between Venus and Earth with up to 70% success (disk Ix). However, even for disks without such concentrations, the success rates were approximately 20% (disk D), 30% (disk A) and 40% (disk E) (Table S1). Intriguingly, these findings suggest that the small mutual distance of the Venus–Earth pair resulted from a peculiar initial mass distribution around ~0.8-1 au rather than the effects of the JSCE mechanism.

Both Venus and Earth analogues typically accreted 99% (70%) of their final masses from objects that were initially within ~2 au (0.8–1.2 au) in the disk. Clearly, the core region and adjacent subregions were the primary sources of building blocks for these planets. Nevertheless, the feeding zones of the planets were slightly shifted inward and outward for Venus and Earth, respectively, in agreement with past work^19,139,157^.

*5.4 Late accretion of Earth and the other terrestrial planets.* Because JSCE quickly stirred asteroids beyond 2 au, there was a preference for remnant objects within 2 au to accrete late (> 100 Myr) after Earth’s formation. As these objects originally formed at ~0.8–1.8 au in the disk, Earth accreted mostly dry enstatite and ordinary chondrite (EC and OC) objects during its late accretion. Also, as our disks consisted of 10–20% C-asteroids at < 2 au, this would explain the small contribution of carbon chondrite (CC) materials during the late stages of Earth accretion^38,158^. Conversely, Mercury, Venus, and Mars probably experienced similar late EC/OC accretion histories. Recent models of Mercury formation and Venus’ coupled atmosphere–internal evolution are consistent with this picture^120,144^. In addition, the captured trans-Jovian asteroids summed to a total mass comparable to the current asteroid belt and concentrated beyond 2.5 au (ref.^43^), so their contribution to late accretion was negligible.

*5.5* *Bulk compositions of Earth and Mars*. The composition of the terrestrial planets can be inferred from mixing models of elemental and isotopic compositions^105,140,158,159,160,161,162,163,164^. However, the estimates of the bulk compositions of Earth and Mars have considerable uncertainties. Another question is whether Earth accreted some of its building blocks from a disk component for which no representative meteorites have been identified in the current collections^160,161,165^. In short, there is still no consensus on the best model regarding the bulk compositions of terrestrial planets. Nevertheless, we consider the chondritic models below as proof of concept that our planet analogues could reproduce the compositions of Earth and Mars. More detailed modelling is beyond the scope of this paper.

Despite the uncertainties, the mixing models agree that Earth is made mostly of enstatite chondritic materials. For instance, ref.^159^ suggested that Earth comprises 59-85%, 12-34% and 2-9% enstatite, ordinary and carbonaceous chondritic materials, respectively. For Mars, the intervals are roughly <68%, >32% and <3%, respectively. Here, to estimate the bulk composition of our Earth and Mars analogues, consistent with our primordial asteroid belt taxonomy (Methods), we considered three simple chondritic models with the following fractions: i) EC100% (0–1 au), OC80%-CC20% (1–2 au); ii) EC100% (0–1 au), EC20%-OC70%-CC10% (1–1.5 au), OC90%-CC10% (1.5–2 au) and; iii) EC90%-OC10% (0–1.2 au), OC90%-CC10% (1.2–2 au). Only the planet analogues formed in 4-P systems were considered in this investigation. We found that Earth analogues acquired on average 61/67/70% EC, 30/28/27% OC and 8/4/3% CC materials, whilst for Mars analogues, these results were 55/62/65% EC, 35/33/32% OC and 9/5/3% CC materials for models i/ii/iii, respectively. Although these chondritic models are not unique and similar models could lead to successful results, these results are nevertheless compatible with the literature cited above and within the involved uncertainties. Finally, our asteroid belt model suggests that the disk component at <0.8 au (roughly the inner region) did not provide objects that could survive as asteroids today. Thus, this might be the region of the protoplanetary disk that provided the “missing materials” to Earth and the other planets^140,165^.

*5.6 Mars formation.* 146 Mars analogues formed in our analogue systems with orbits and masses closely akin to the actual planet. In particular, 99 and 47 Mars analogues formed in 3-P and 4-P systems, respectively (Table S4). Overall, the little dependence of the results on model details indicates that the JSCE scenario is robust in reproducing Mars. In addition, because 40–50% of the final masses of our Mars analogues were fed by objects located beyond 1 au, JSCE’s perturbations helped to prevent our disks from producing overmassed Mars analogues.

As discussed in Section 1, Mars could have formed in timescales slightly longer than the canonical 10 Myr if its formation was protracted^98,99,100,101,102^. Here, the success rates for the formation timescales of our Mars analogues were ~10-40% in the main disk models (Table S5). Similarly, the median formation timescales were marginally compatible with the upper limits of our success criteria. However, Mars analogues typically experienced 1-3 giant impacts during formation. In particular, a formation timescale longer than ~15 Myr was often determined by the time of the last giant impact in unsuccessful cases. Further studies are warranted to understand better the role of giant impacts in the accretion history of Mars. Also, aiming at obtaining shorter Mars’ formation timescales, future N-body simulations could reveal more favourable initial conditions, especially focusing on embryo individual mass, orbital range of embryos in the disk, the total mass in the disk beyond ~1 au (and the ratio of that mass in embryos and planetesimals), and dynamical evolution of the Jupiter–Saturn pair.

Finally, our Mars analogues typically experienced the last collision by impactors of ≥ 0.005 and ≥ 0.001 ME masses after medians of 63–109 and 134–142 Myr, respectively. Such collisions are consistent with those proposed for the origin of the dichotomy and the Borealis basin on Mars^64,166^.

*5.7 Water delivery to the four terrestrial planets.* By analysing the water mass acquired by our planet analogues formed in the standard disk (Methods), we found that typically 0.01%, 0.01–0.5%, 0.5–10% and 10–40% WMFs for regions at < 1.5 au, 1.5–2 au, 2–2.5 au and 2.5–3.5 au were required to satisfy the terrestrial planets’ water constraint (Section 1). Alternatively, if the disk were relatively dry at < 2 au (< 0.01%), the > 2 au region would need to be more water-rich with WMFs > 20%. Overall, our results imply that: 1. The inner regions of the disk at < 2 au contained more water than previously thought; 2. The disk was several times more water-rich at > 2 au than at < 2 au. Recent observations and analyses of S-type asteroids, meteorites, and primitive organic matter support point 1 (ref.^38,39,167,168^), whilst measurements of WMFs of 10–20% in C-asteroids^123,169^, the expectation of high WMFs in D/P-asteroids^170,171^ and the contribution of trans-Jovian objects captured as asteroids beyond ~2–2.5 au (ref.^40,43,85,172^) support point 2. The water ice line probably contributed similarly by enhancing the formation of water-rich asteroids in that region^26,80,81,82^. Finally, the contamination of C- and D/P-asteroids in our composition model (Methods and Section 5.8) is consistent with the above results regarding higher WMFs in the protoplanetary disk.

Based solely on the successful WMF models (Table S2), the median WMFs acquired by Mercury analogues in the standard disk varied within the interval 7.5 × 10^−5^–9.4 × 10^−4^ over these models. Similar values are expected in representative disk Ix because our Mercury analogues generally acquired their water from objects beyond ~1.5 au regardless of the disk considered. This result strengthens the case that Mercury accreted non-negligible amounts of water during its formation. By performing a similar analysis for our Venus, Earth and Mars analogues, we obtained the following median WMF intervals acquired by these planets: 4.2 × 10^−4^–1.1 × 10^−3^, 5.0 × 10^−4^–1.2 × 10^−3^ (~2–5 Earth’s oceans) and 2.8 × 10^−4^–1.5 × 10^−3^, respectively. Therefore, Venus, Earth and Mars probably acquired significant water masses during their formation. Similar to Earth, JSCE allowed a quick delivery of water-rich objects initially located beyond ~1.5 au to the other terrestrial planets.

The 80 WMF models considered in this study are not unique, and other combinations of individual water fractions in each disk region could lead to successful results. In particular, as suggested by our results, more water-rich disks not modelled here could potentially explain the WMFs of Venus, Earth and Mars, thus yielding Earth with higher median water contents and higher contribution from disk objects located within ~1.5 au. Finally, a caveat is that the water acquired by our obtained planets represents upper limits for the models considered because water retention was not 100% efficient as assumed in our calculations^173^.

*5.8 Origin and evolution of the asteroid belt.*

*a.1) Formation: general picture*. Giant-planet migration/instability and terrestrial-planet formation played essential roles in dynamically shaping the asteroid belt^6,16,17,18,32,35,84^. The orbital architecture of the current giant planets predicts an asteroid belt stable up to ~4 au. However, asteroids concentrate highly within 3.25 au, without a substantial population of asteroids located beyond this region. This fact implies that the giant planets may have played essential roles in dynamically sculpting this distant region (~3.25–4 au) or that the primordial asteroid belt did not extend beyond 3.25 au during the infancy of the solar system. Indeed, there is evidence that distant asteroids located beyond 3.25 au originated as captured trans-Jovian objects^84,172^.

Our scenario reproduced all of these features. An eccentric Jupiter gravitationally destabilised the primordial asteroid belt beyond ~3.2 au, and JSCE strongly shaped and dynamically depleted the primordial asteroid belt. Later, captured asteroids populated the outer regions of the belt at the end of planetary migration (ref.^43^, this study). As discussed in the main text, we concluded that the admixing of different types of asteroids in the current asteroid belt is evidence of local asteroids that survived the various perturbations by the giant planets (mainly JSCE) and captured asteroids from trans-Jovian objects that acquired stable orbits after planetary migration.

*a.2) Formation: additional discussion and caveats*. The asteroid belt probably experienced four main stages of evolution during solar system history: 1. disk gas dispersal, 2. JSCE, 3. instability, and 4. long-term. When investigating asteroid belt formation, our main simulations took into account stages 2 and 4, whilst our auxiliary simulations considered the effects of stage 3, as discussed below.

Concerning stage 1, if Jupiter and Saturn acquired their current (*a*_J_ = 5.20 au, *a*_S_ = 9.55 au, *e*_JS_ ~ 0.05) or more eccentric orbits (*a*_J_, *a*_S_ idem, *e*_JS_ ~ 0.1) during the solar system’s gas dispersal stage, the sweeping of the nu5 secular resonance could have depleted the primordial asteroid belt beyond ~2 au (ref.^94,141,142,174,175^). Nonetheless, given the various parameters associated with disk gas dynamics and disk-giant planet interactions, past studies have explored only a limited set of parameters and giant planet configurations regarding this mechanism. For instance, as predicted by hydrodynamic models (Methods), the cases of Jupiter and Saturn locked in a MMR (3:2 or 2:1) with low-moderate eccentricities (~0.01-0.l) remain unexplored. Other effects, such as gap-opening, also remain unexplored in recent studies^94,141,142^. In addition, if the gas dispersal were fast (< several hundred kyr) as considered in some models^40^, then the effects of the sweeping nu5 secular resonance would probably be much less significant. Similarly, for rapid gas dispersal *after* Jupiter formation (~0.1 Myr timescales or shorter), these effects would be negligible^174^. Another critical point is that hydrodynamic modelling predicts many possible eccentricity evolutions for Jupiter and Saturn interacting in the 2:1 MMR, so it is possible that the giant planets experienced *e* ≤ 0.05 during that interaction^56,57,174^. Finally, residual embryos/planetesimals may have reduced the eccentricities of the giant planets via dynamical friction during stage 1. Considering the reasoning above, we conclude that our assumption that the primordial asteroid belt was massive and extended to 3.5 au after disk gas dispersal is conceivable.

Regarding stage 3, the role of the instability in perturbing the primordial asteroid belt depends on the instability’s details and the belt’s initial dynamical state. For instance, Jupiter’s orbital evolution behaviour (i.e., how “jumpy” the evolution is), duration of the instability, and scattering events with icy giants are important factors^6,16,17,84^. While the instability can stir an initially cold primordial asteroid belt, it can shuffle the eccentricities of an initially hot belt. Consistent with the latter scenario, for our excited primordial asteroid belt (by JSCE) (Figure S4a), there is evidence that the instability *does not* significantly alter the final orbital structure acquired by the asteroid belt after Gyr timescales^84,145^. Nevertheless, ref.^145^ found that the instability increased long-term depletion in the asteroid belt by 60%. Based on our optimal instability systems, we found a median long-term depletion increase of 27–34% (Section 3.4). These simulations also confirmed that the asteroids that survived 4 Gyr after the instability have similar *a-e-i* structure compared to those in which the instability was simplified by instantaneously transporting the giant planets to their current orbits, as done in our baseline model and previous studies^45,71^. Despite that, we found that the instability increased the f6 ratio in our optimal systems. Compared to our control simulation in this investigation (f6 = 0.12), the optimal systems yielded f6 = 0.21–0.28, or an increase of 75-133%. Based on our main simulations, the representative asteroid belt yielded f6 = 0.16–0.19 after assuming reasonable proportions of local and captured asteroids (Methods). In this way, by considering the effects of instability and the final phase of residual migration^146^ (Section 2.5), we obtained f6 = 1.75 × 0.4 × (0.16–0.19) = 0.11–0.13 or 2.33 × 0.4 × (0.16–0.19) = 0.15–0.18. Therefore, we estimate a final f6 is lying within 0.11–0.18 in this work.

Of course, our representative asteroid belt is not a perfect match for the real belt. For example, there is a deficit of asteroids at 2.1-2.3 au. This specific region contains 23/895 = 2.6% and 2/(159 or 318) = 0.6-1.3% of real and model asteroids, respectively. The latter fraction varies according to the proportion of local:captured asteroids adopted. Potential solutions to this discrepancy include asteroids’ self-gravity^16^ (not modelled here) and the instability^176^. Under the effect of these mechanisms, asteroids can experience radial displacements that could help populate the inner region within 2.3 au. Based on our auxiliary simulations of stage 3 described above, we noticed a small increase of asteroids in the inner region. Another potential solution is that slightly different initial conditions for the Jupiter–Saturn pair might reduce the depletion levels of primordial asteroids in the 2-2.5 au region.

Overall, as discussed in the main text, our asteroid belt model broadly explains the orbital structure of the asteroid belt.

*b) Small mass.* The current asteroid belt is up to several thousand times less massive than predicted at 2–4 au if the disk that formed the terrestrial planets followed the same mass distribution until 4 au (ref.^177^). As collisional grinding cannot explain this mass discrepancy, either the giant planets played significant roles in the mass depletion of the primordial asteroid belt or the latter was not massive^8,9,10,12,18,14,32,130^.

Our model can potentially solve the puzzling small mass of the asteroid belt. At the end of terrestrial-planet formation (after ~100 Myr in our simulation framework; henceforth ‘t100’), the median masses and survival fractions for asteroid belts found in the standard disk were ~5.0 × 10^−3^ ME and 0.70% (46 systems). After evolving these systems over 4 Gyr, 81% (445/551) of the asteroids were removed. These results were consistent with 4-Gyr depletion levels of 50–60% in belts based on the observed one^131,132^, ~70% in post-Grand-Tack systems^45^, and (50) 80% for systems that experienced (no) dynamical instabilities in ref.^145^. The initial orbital states of the asteroid belt that yielded 50–60% depletion in past work were similar to the observed belt, while our asteroid belts exhibited a wider range of eccentricities and inclinations as obtained directly from the simulations. Thus, our asteroid belts covered more unstable areas of orbital space resulting in higher removal fractions. Therefore, by combining the fractions above, we found a primordial asteroid belt depletion level of 99.87%.

In conclusion, the final mass of our representative asteroid belt resulted in ~1 × 10^−3^ ME in local asteroids. Considering the additional depletion caused by the instability of 27–34%, this estimate reduces to ~7 × 10^−4^ ME, which after combining with the complement mass in captured asteroids resulted in an asteroid belt with mass (1.5±0.5) × 10^−3^ ME depending on the fractions of local/captured asteroids adopted in the current asteroid belt. This result translates into a factor of 2-4 times the mass in the observed belt. We suggest the following hypotheses to reconcile this result with the observed mass of the asteroid belt: 1. The primordial asteroid belt was initially less massive (e.g., ≤0.5-1 ME); 2. The presence of small embryos in the primordial asteroid belt provided additional depletion^130,178^; 3. Given the spectrum of possible instability evolutions^9,22,51,57,58,67^, the instability experienced by the giant planets yielded smaller fractions of local and captured asteroids.

We conclude that our representative asteroid belt acquired a small mass in reasonable agreement with observations.

*c) Composition taxonomy.* By applying our nominal model of primordial asteroid compositions to local and captured asteroids, we found that our representative asteroid belt could explain the currently observed orbital concentrations of S-, C- and D/P-type asteroids^15,123^ (Figures 4, S7 and S8). We also tested several distinct fractions of S-, C- and D/P assigned to the local and captured asteroids to verify dependence on model parameters. Compared to the nominal model (S90~80%-C10~20% within < 2 au and S50%-C50% beyond > 2 au for local asteroids and C50%-DP50% for captured asteroids), a higher or lower fraction of C-asteroids beyond 2 au among local asteroids resulted in too many or too few final C-type asteroids within the same region. Conversely, increasing the proportion of S-asteroids (fewer C-asteroids) also led to too many final S-type asteroids in this region. Therefore, while a S50%-C50% contribution beyond > 2 au was favoured, a contribution of C-asteroids at < 2 au (i.e., fewer S-asteroids) was needed to match the final S-type asteroid distribution better. Although we could not strongly constrain the proportion of C-asteroids at < 2 au, matching the observed C- and S-type asteroids in the inner asteroid belt required C-asteroids with ~10–20% abundances within this region. About captured asteroids, a higher fraction of C-asteroids (smaller fraction of D/P-asteroids) resulted in too many final C-type and a lack of D/P-type asteroids beyond ~2.9 au. Conversely, a higher fraction of D/P-asteroids resulted in too many D/P-type and a paucity of C-type asteroids within the same region. Furthermore, the reproduction of distant S-type asteroids beyond 2.9 au may require a small contribution of S-asteroids (~5%) among the captured population. All these trends are valid for an asteroid belt consisting of comparable local and captured asteroids with proportions within a factor of 2.

For completeness, we created two additional WMF models based on the nominal composition model by assuming that the WMFs of C- and D/P-asteroids were 5% or 10% and 20% or 30%, respectively (models 27 and 28 in Table S2). The wetter WMF model of C-asteroids (10%) was successful for our Venus, Earth and Mars analogues, independent of the choice of WMF for D/P-asteroids. Therefore, we confirmed that our results were self-consistent regarding asteroidal compositions and water delivery.

*d) Late accretion and the inner solar system’s bombardment.* After t100 and the Moon-forming giant impact, our results imply that Earth experienced non-negligible accretion by impactors over 4 Gyr. In the *a*-*e*-*i* element region (0.7–2 au, 0–0.65, 5–50°) that sourced 137 of the 142 impactors to Earth over 4 Gyr, there were initially 1985 particles and a total of 0.026 ME. Thus, 0.026 × (137/1985) = 0.0018 ME of mass accreted to Earth during this period. We also identified several late collisions with Earth well after t100 over timescales of hundreds of Myr, thus supporting the hypothesis that terrestrial-planet formation tail-end population caused a bombardment in the inner solar system^179,180^. These results were also consistent with the findings of ref.^46,64^.

*e) The residual EE-belt.* In agreement with previous studies, our results also support the existence of a primordial disk component located beyond the orbit of Mars akin to the hypothesised ‘E-belt’ (ref.^71^). However, while the latter was assumed to be a relatively stable component of asteroids at *a* = 1.6–2.1 au, perihelia *q* < 1.6 au and *i* < 20°, our results instead predict an excited E-belt (EE-belt) with a more comprehensive range of eccentricities and inclinations persisting during and after terrestrial-planet formation. For *a* = 1.6–2.1 au and without restricting *q* or *i*, we obtained a median of 0.015 ME for the EE-belt or ~25 times the mass of the current asteroid belt at t100. Approximately one order of magnitude less mass is obtained if *q* < 1.6 au and *i* < 20° are applied. Briefly, our study revealed that an EE-belt could have arisen naturally during/after the formation of the terrestrial planets.

*f) Emptied primordial asteroid belt*. Notably, a long-term JSCE may have depleted the primordial asteroid belt entirely. In such a scenario, the asteroid belt might have formed due to the implantation of S-type objects scattered out from the terrestrial region during terrestrial-planet formation and implantation of trans-Jovian C-type objects during Jupiter–Saturn interactions and post-instability evolution^18,40,43^. However, it is currently unclear whether this alternative scenario could consistently explain the formation of the four terrestrial planets and the orbital structure, the f6 constraint, the small total mass and the taxonomy of the asteroid belt. See also Section 2 for complementary discussions.

6. MAIN FINDINGS AND IMPLICATIONS

Overall, after considering several plausible initial conditions for the early solar system in the framework of the JSCE scenario, our results identified those with good prospects for explaining the terrestrial planets and the asteroid belt. We scrutinised our terrestrial planet systems with unprecedented detail and identified several that successfully satisfied fundamental constraints in the inner solar system. These results imply that the terrestrial planets probably formed from a protoplanetary disk containing mass concentrated within a core region surrounded by a low-mass inner region and a mass-depleted and dynamically perturbed outer region. Furthermore, JSCE played a crucial role in shaping the disk. Finally, the asteroid belt formed by local asteroid remnants and captured trans-Jovian objects that survived until this period.

If correct, our main findings can be summarised as follow.

Our disks can produce 4-P systems and reasonably reproduce the orbits, masses, accretion histories and other aspects of the four terrestrial planets. In particular:

● Mercury formed within the low-mass extended inner region;

● The Venus–Earth pair formed from a mass-concentrated thin annulus within the core region;

● Mars formed within the mass-depleted outer region;

● The existence of the inner and outer regions allowed the formation of Mercury and Mars at the correct distances from Venus and Earth, respectively.

Earth experienced the Moon-forming giant impact(s) within 60 Myr after solar-system formation. The impactor(s) was(were) less massive than Mars, with a preference of 0.02 ≤ *m* < 0.1 ME. After the last giant impact, late accretion of dry EC/OC-like objects (originally formed at 0.8–1.8 au) contributed to < 0.01 ME of Earth’s final mass.

Disks containing a drier region within 1.5 au and a wetter region beyond ~1.5-2 au yielded good prospects for delivering water to Earth and the other terrestrial planets. Earth’s bulk water was acquired during the first 10–20 Myr of its accretion, before the formation of the Moon. The other terrestrial planets also acquired significant amounts of water.

Our representative asteroid belt can reproduce the *a*-*e*-*i* orbital structure, the mixed populations of S-, C- and D/P-type asteroids, the small total mass and other features of the asteroid belt. Furthermore, at the end of the terrestrial-planet formation, a reservoir of primordial asteroids with *a* = 1–2 au and *i* = 15°–50° sourced late impacts on Earth over several hundred Myr. Thus, terrestrial-planet formation tail-end asteroids can explain the inner solar system’s bombardment.

Jupiter and Saturn interacted near their mutual 2:1 MMR for ~5–10 Myr timescales before experiencing their dynamical instability/migration and acquiring their current orbits. Under this orbital configuration, secular resonances associated with both the giant planets behaved chaotically, resulting in strong dynamical excitation and depletion of the disk beyond ~1–1.5 au, including >99% depletion of a massive primordial asteroid belt. This event allowed the formation of a small-mass Mars on a moderately excited orbit and a very low-mass and stirred asteroid belt. Then, after planetary migration was complete, captured asteroids from the trans-Jovian region populated the asteroid belt. Nowadays, the asteroid belt consists of comparable populations of local and captured asteroids.

**References**

1. Clement, M. S., Chambers, J. E., Jackson, A. P. Dynamical Avenues for Mercury’s Origin. I. The Lone Survivor of a Primordial Generation of Short-period Protoplanets. Astron. J. 161, id.240, 15 pp (2021).
2. Dauphas, N., Pourmand, A. Hf-W-Th evidence for rapid growth of Mars and its status as a planetary embryo. Nature 473, 489-492 (2011).
3. Mezger, K., Debaille, V., Kleine, T. Core Formation and Mantle Differentiation on Mars. Space Science Reviews 174, 27-48 (2013).
4. Marchi, S., Walker, R. J., Canup, R. M. A compositionally heterogeneous martian mantle due to late accretion. Sci. Adv. 6, eaay2338 (2020).
5. Zhang, Z., Bercovici, D., Jordan, J. S. A Two Phase Model for the Evolution of Planetary Embryos With Implications for the Formation of Mars. Journal of Geophysical Research: Planets 126, id. e06754 (2021).
6. [Kruijer, T. S.,](https://www.sciencedirect.com/science/article/pii/S0012821X17303710#!) et al.The early differentiation of Mars inferred from Hf–W chronometry. Earth and Planetary Science Letters 474, 345-354 (2017).
7. Borg, L. E., Brennecka, G. A., Symes, S. J. K. Accretion timescale and impact history of Mars deduced from the isotopic systematics of martian meteorites. Geochimica et Cosmochimica Acta 175, 150-167. (2016).
8. McCubbin, F. M., Barnes, J. J. Origin and abundances of H2O in the terrestrial planets, Moon, and asteroids. Earth and Planetary Science Letters 526, id. 115771 (2019).
9. Albarede, F. Volatile accretion history of the terrestrial planets and dynamic implications. Nature 461, 1227-1233 (2009).
10. Marty, B. The origins and concentrations of water, carbon, nitrogen and noble gases on Earth. Earth and Planetary Science Letters 313, 56-66 (2012).
11. Lock, S. J., Bermingham, K. R., Parai, R., Boyet, M. Geochemical Constraints on the Origin of the Moon and Preservation of Ancient Terrestrial Heterogeneities. Space Sci Rev 216, 109 (2020).
12. Kleine, T., et al. Hf–W chronology of the accretion and early evolution of asteroids and terrestrial planets. Geochimica et Cosmochimica Acta 73, 5150-5188 (2009).
13. Canup, R. M., Asphaug, E. Origin of the Moon in a giant impact near the end of the Earth’s formation. Nature 412, 708-712 (2001).
14. Canup, R. M. Forming a Moon with an Earth-like Composition via a Giant Impact. Science 338, 1052- (2012).
15. Jackson, A. P., Gabriel, T. S. J., Asphaug, E. I. Constraints on the pre-impact orbits of Solar system giant impactors. Mon. Not. R. Astron. Soc. 474, 2924-2936 (2018).
16. Cuk, M., Stewart, S. T. Making the Moon from a Fast-Spinning Earth: A Giant Impact Followed by Resonant Despinning. Science 338, 1047-1052 (2012).
17. Rufu, R., Aharonson, O., Perets, H. B. A multiple-impact origin for the Moon. Nature Geoscience 10, 89-94 (2017).
18. Lock, S. J., et al. The origin of the moon within a terrestrial synestia. J. Geophys. Res. Planets 123, 910–951 (2018).
19. Reufer, A., Meier, M. M. M., Benz, W., Wieler, R. A hit-and-run giant impact scenario. Icarus 221, 296–299 (2012).
20. Asphaug, E., Emsenhuber, A., Cambioni, S., Gabriel, T. S. J., Schwartz, S. R. Collision Chains among the Terrestrial Planets. III. Formation of the Moon. The Planetary Science Journal, 2:200 (2021).
21. Canup, R. M., et al. Origin of the Moon. Preprint at http://arxiv.org/abs/2103.02045 (2021).
22. Jacobson, S. A., et al. Highly siderophile elements in Earth’s mantle as a clock for the Moon-forming impact. Nature 508, 84-87 (2014).
23. Piet, H., Badro, J., Gillet, P. Geochemical Constraints on the Size of the Moon-Forming Giant Impact. Geophysical Research Letters 44, 11,770-11,777 (2017).
24. Citron, R. I., Perets, H. B., Aharonson, O. The Role of Multiple Giant Impacts in the Formation of the Earth-Moon System. Astrophys. J. 862, id. 5, 11 pp (2018).
25. Gillmann, C., et al. Dry Late Accretion inferred from Venus’ coupled atmosphere and internal evolution. Nature Geoscience 13, 265-269 (2020).
26. Fischer-Godde, M., Kleine, T. Ruthenium isotopic evidence for an inner Solar System origin of the late veneer. Nature 541, 525-527 (2017).
27. Marchi, S., Canup, R. M., Walker, R. J. Heterogeneous delivery of silicate and metal to the Earth by large planetesimals. Nature Geoscience 11, 77-81 (2018).
28. O’Brien, D. P., Izidoro, A., Jacobson, S. A., Raymond, S. N., Rubie, D. C. The Delivery of Water During Terrestrial Planet Formation. Space Science Reviews 214, id. 47, 24 pp (2018).
29. O’Brien, D. P., Walsh, K. J., Morbidelli, A., Raymond, S. N., Mandell, A. M. Water delivery and giant impacts in the ‘Grand Tack’ scenario. Icarus 239, 74-84 (2014).
30. Venturini, J., Ronco, M. P., Guilera, O. M. Setting the Stage: Planet Formation and Volatile Delivery. Space Sci. Rev. 216, 86 (2020).
31. Lunine, J. I., Chambers, J., Morbidelli, A., Leshin, L. A. The origin of water on Mars. Icarus 165, 1-8 (2003).
32. Taylor, G. J. The bulk composition of Mars. Chemie der Erde 73, 401-420 (2013).
33. Kurokawa, H., et al. Evolution of water reservoirs on Mars: Constraints from hydrogen isotopes in martian meteorites. Earth and Planetary Science Letters 394, 179-185 (2014).
34. Rickman, H., et al. Water in the history of Mars: An assessment. Planetary and Space Science 166, 70-89 (2019).
35. O’Brien, D. P., Morbidelli, A., Bottke, W. F. The primordial excitation and clearing of the asteroid belt—Revisited. Icarus 191, 434-452 (2007).
36. Minton, D. A., Malhotra, R. A record of planet migration in the main asteroid belt. Nature 457, 1109-1111 (2009).
37. Minton, D. A., Malhotra, R. Dynamical erosion of the asteroid belt and implications for large impacts in the inner Solar System. Icarus 207, 744-757 (2010).
38. Emery, J. P., Burr, D. M., Cruikshank, D. P. Near-infrared spectroscopy of trojan asteroids: evidence for two compositional groups. Astron. J. 141:25 (2011).
39. Brasser, R., Matsumura, S., Ida, S., Mojzsis, S. J., Werner, S. C. Analysis of terrestrial planet formation by the grand tack model: system architecture and tack location. Astrophys. J. 821:75 (2016).
40. Chambers, J. E. Pebble Accretion and the Diversity of Planetary Systems. Astrophys. J. 825, id. 63, 18 pp (2016).
41. Bromley, B. C., Kenyon, S. J. Terrestrial Planet Formation: Dynamical Shake-up and the Low Mass of Mars. Astronom. J. 153, id. 216, 17 pp (2017).
42. Jacobson, S. A., Morbidelli, A. Lunar and terrestrial planet formation in the Grand Tack scenario. Phil. Trans. R. Soc. A 372, id. 0174 (2014).
43. Lykawka, P. S., Ito, T. Terrestrial planet formation during the migration and resonance crossings of the giant planets. Astrophys. J. 773:65 (2013).
44. Mah, J., Brasser, R. Isotopically distinct terrestrial planets via local accretion. Icarus 354, 114052 (2021).
45. Burkhardt, C. et al. Terrestrial planet formation from lost inner solar system material. Sci. Adv. 7, eabj7601 (2021).
46. Morishima, R., Stadel, J., Moore, B. From planetesimals to terrestrial planets: N-body simulations including the effects of nebular gas and giant planets. Icarus 207, 517-535 (2010).
47. Thommes, E., Nagasawa, M., Lin, D. N. C. Dynamical shake-up of planetary systems. Ii. N-body simulations of solar system terrestrial planet formation induced by secular resonance sweeping. Astrophys. J. 676, 728–739 (2008).
48. Lykawka, P. S., Ito, T. Terrestrial Planet Formation: Constraining the Formation of Mercury. Astrophys. J. 838, id. 106, 10 pp (2017).
49. Clement, M. S., Raymond, S. N., Chambers, J. E. Mercury as the Relic of Earth and Venus Outward Migration. Astrophys. J. Letters 923, id.L16, 8 pp (2021).
50. Nesvorny, D., Roig, F., Bottke, W. F. Modeling the Historical Flux of Planetary Impactors. Astron. J. 153:103, 22pp (2017).
51. Clement, M. S., Morbidelli, A., Raymond, S. N., Kaib, N. A. A record of the ﬁnal phase of giant planet migration fossilized in the asteroid belt’s orbital structure. Mon. Not. R. Astron. Soc. 492, L56–L60 (2020).
52. Chambers, J. E. On the stability of a planet between Mars and the asteroid belt: Implications for the Planet V hypothesis. Icarus 189, 386–400 (2007).
53. Brasser, R., Morbidelli, A. The terrestrial Planet V hypothesis as the mechanism for the origin of the late heavy bombardment. Astron. Astrophys. 535, A41 (2011).
54. Dodson-Robinsona, S. E., Willacy, K., Bodenheimer, P., Turner, N. J., Beichman, C. A. Ice lines, planetesimal composition and solid surface density in the solar nebula. Icarus 200, 672–693 (2009).
55. Lichtenberg, A., Lieberman, M. Regular and Chaotic Dynamics. Regular and Chaotic Dynamics, Second Edition, by A. Lichtenberg and M. Lieberman. Springer-Verlag, New York, ISBN 0-387-97745-7 (1992).
56. Sussman, G. J., Wisdom, J. Chaotic Evolution of the Solar System. Science 257, 56-62 (1992).
57. Laskar, J. Chaotic diffusion in the Solar System. Icarus 196, 1-15 (2008).
58. Laskar, J., Gastineau, M. Existence of collisional trajectories of Mercury, Mars and Venus with the Earth. Nature 459, 817-819 (2009).
59. Morbidelli, A., Levison, H. F., Tsiganis, K., Gomes, R. Chaotic capture of Jupiter’s Trojan asteroids in the early Solar System. Nature 435, 462-465 (2005).
60. Hyodo, R., Genda, H., Brasser, R. Modification of the composition and density of Mercury from late accretion. Icarus 354, 114064 (2021).
61. Asphaug, E., Reufer, A. Mercury and other iron-rich planetary bodies as relics of inefficient accretion. Nature Geoscience 7, 564-568 (2014).
62. Kaib, N. A, Cowan, N. B. The feeding zones of terrestrial planets and insights into Moon formation. Icarus 252, 161–174 (2015).
63. Dauphas, N. The isotopic nature of the Earth’s accreting material through time. Nature 541, 521-524 (2017).
64. Brasser, R., Dauphas, N., Mojzsis, S. J. Jupiter’s Influence on the Building Blocks of Mars and Earth. Geophysical Research Letters 45, 5908-5917 (2018).
65. Drake, M. J., Righter, K. Determining the composition of the Earth. Nature 416, 39-44 (2002).
66. Carlson, R. W., Brasser, R., Yin, Q.-Z., Fischer-Godde, M., Qin, L. Feedstocks of the Terrestrial Planets. Space Sci. Rev. 214:121 (2018).
67. Liebske, C., Khan, A. On the principal building blocks of Mars and Earth. Icarus 322, 121–134 (2019).
68. Savage, P. S., Moynier, F., Boyet, M. Zinc isotope anomalies in primitive meteorites identify the outer solar system as an important source of Earth’s volatile inventory. Icarus 386, 115172 (2022).
69. Steller, T., Burkhardt, C., Yang, C., Kleine, T. Nucleosynthetic zinc isotope anomalies reveal a dual origin of terrestrial volatiles. Icarus 386, 115171 (2022).
70. Mezger, K., Schonbachler, M., Bouvier, A. Accretion of the Earth—Missing Components?. Space Sci. Rev. 216:27 (2020).
71. Marinova, M. M., Aharonson, O., Asphaug, E. Mega-impact formation of the Mars hemispheric dichotomy. Nature 453, 1216-1219 (2008).
72. Nakano, H., et al. Precometary organic matter: A hidden reservoir of water inside the snow line. Scientific Reports 10, 7755 (2020).
73. Jin, Z., Bose, M., Lichtenberg, T., Mulders, G. D. New Evidence for Wet Accretion of Inner Solar System Planetesimals from Meteorites Chelyabinsk and Benenitra. The Planetary Science Journal 2, id.244, 14 pp (2021).
74. Bermingham, K. R., Furi, E., Lodders, K., Marty, B. The NC-CC Isotope Dichotomy: Implications for the Chemical and Isotopic Evolution of the Early Solar System Space Sci. Rev. 216, 133 (2020).
75. Hiroi, T., Zolensky, M. E., Pieters, C. M. The Tagish Lake Meteorite: A Possible Sample from a D-Type Asteroid. Science 293, 2234-2236 (2001).
76. Marchis, F., et al. A low density of 0.8gcm-3 for the Trojan binary asteroid 617Patroclus. Nature 439, 565-567 (2006).
77. Levison, H. F., et al. Contamination of the asteroid belt by primordial trans-Neptunian objects. Nature 460, 364-366 (2009).
78. Burger, C., Bazso, A., Schafer, C. M. Realistic collisional water transport during terrestrial planet formation. A&A 634, A76 (2020).
79. Chametla, R. O., D’Angelo, G., Reyes-Ruiz, M., Sanchez-Salcedo, F. J. Capture and migration of Jupiter and Saturn in mean motion resonance in a gaseous protoplanetary disc. MNRAS 492, 6007–6018 (2020).
80. Nagasawa, M., Tanaka, H., Ida, S. Orbital evolution of asteroids during depletion of the solar nebula. Astron. J. 119:1480-1497 (2000).
81. Brasil, P. I. O., et al. Dynamical dispersal of primordial asteroid families. Icarus 266, 142–151 (2016).
82. Hayashi, C. Structure of the Solar Nebula, Growth and Decay of Magnetic Fields and Effects of Magnetic and Turbulent Viscosities on the Nebula. Progress of Theoretical Physics Supplement 70, 35-53 (1981).
83. Petit, J., Morbidelli, A., Chambers, J. The primordial excitation and clearing of the asteroid belt. Icarus 153, 338–347 (2001).
84. Bottke, W. F., Norman, M. D. The Late Heavy Bombardment. Annual Review of Earth and Planetary Sciences 45, 619-647 (2017).
85. Strom, R. G., Malhotra, R., Ito, T., Yoshida, F., Kring, D. A. The Origin of Planetary Impactors in the Inner Solar System. Science 309, 1847-1850 (2005).

# Supplementary Figures

#
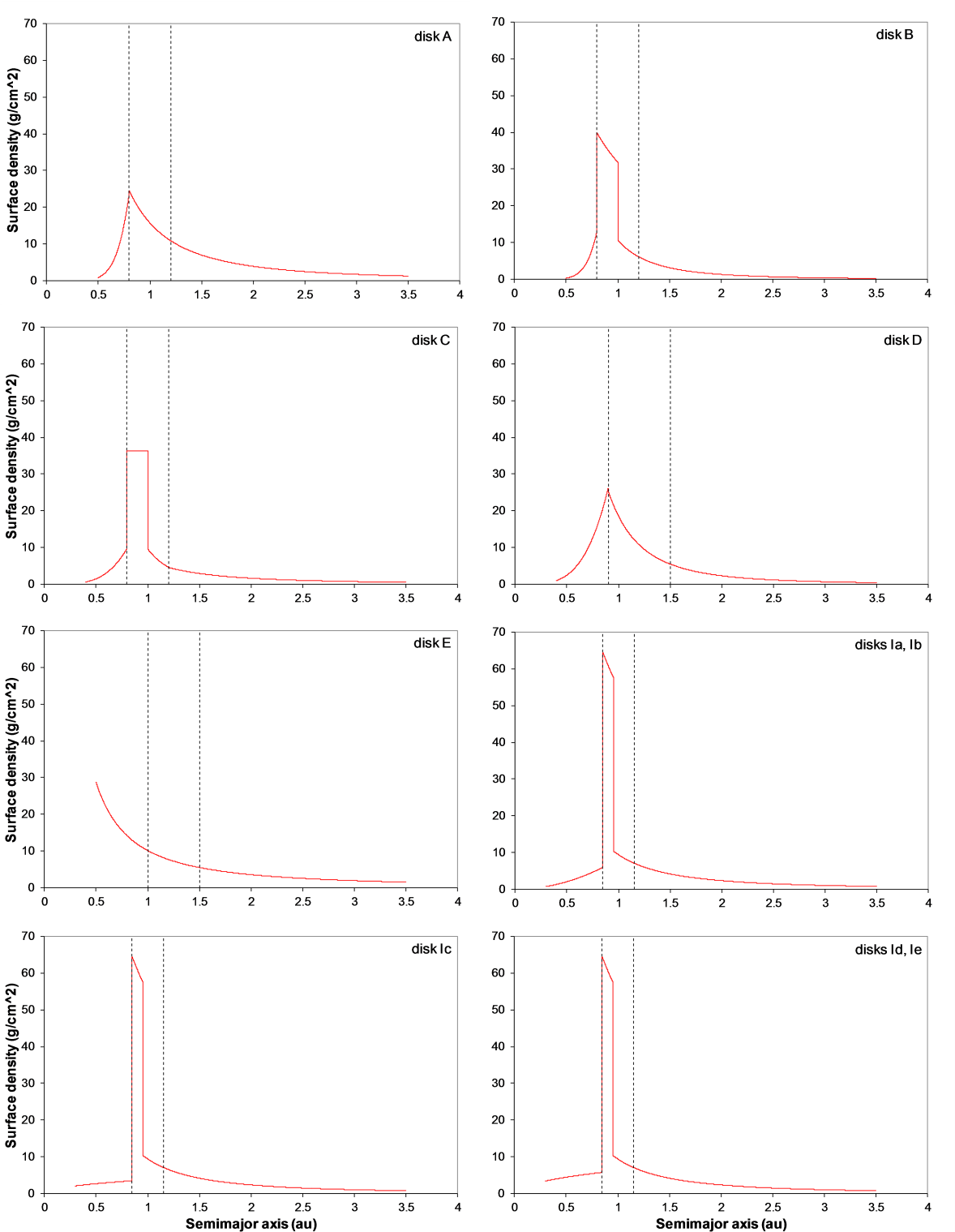


**Figure S1***.* Initial mass distributions of the disk models used in this work. The dashed lines indicate the boundaries of the core region. See Methods and Table S1 for more details.


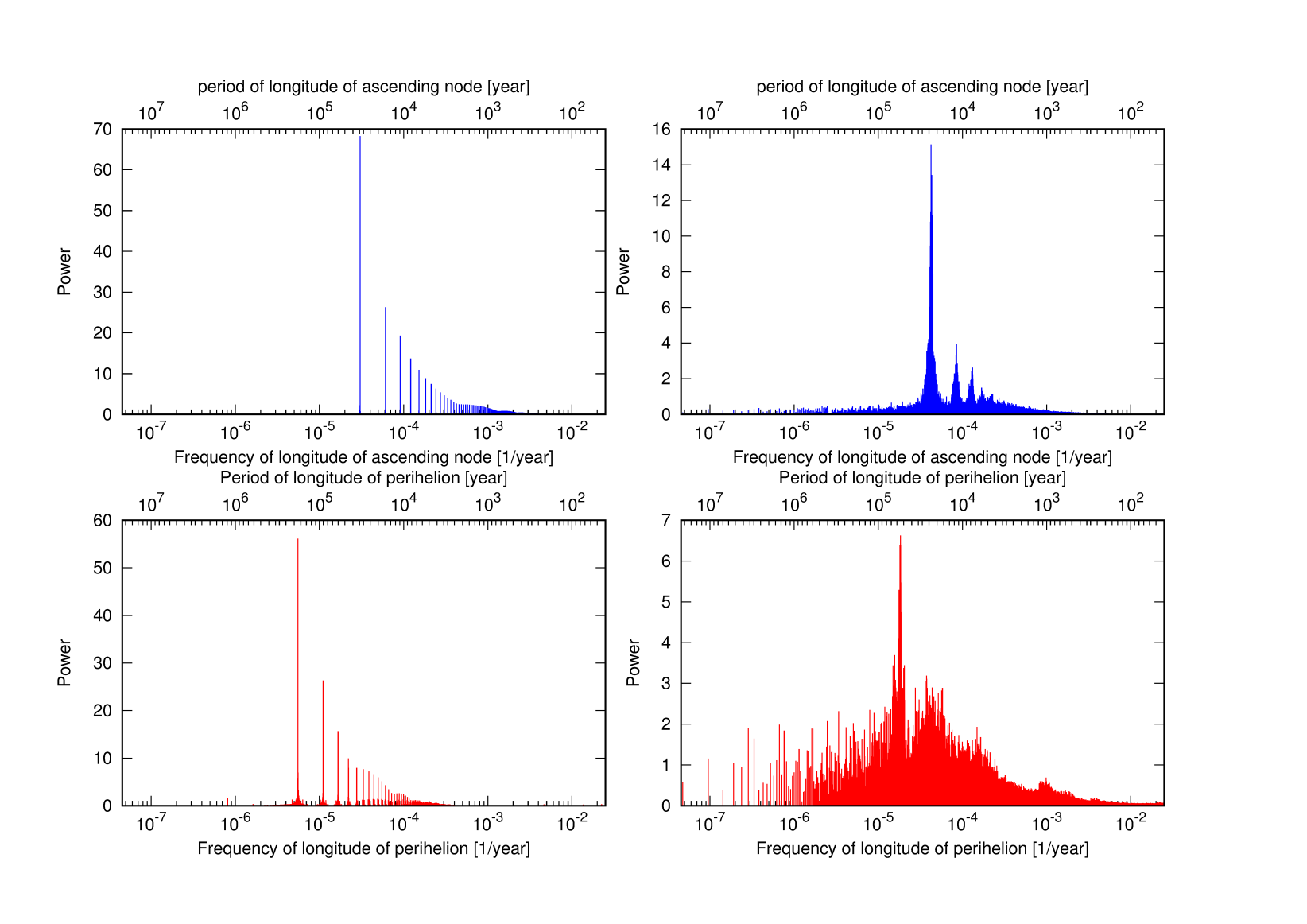


**Figure S2.** Power spectra obtained by Fourier analysis of the longitude of the ascending node (top panels) and longitude of the perihelion (bottom panels) of Saturn. The left and right panels illustrate Saturn’s non-chaotic and chaotic behaviour from two simulations where the Jupiter–Saturn pair and a disk of primordial asteroids evolved to 10 Myr, respectively. In the chaotic case, Jupiter and Saturn were placed near their mutual 2:1 MMR.


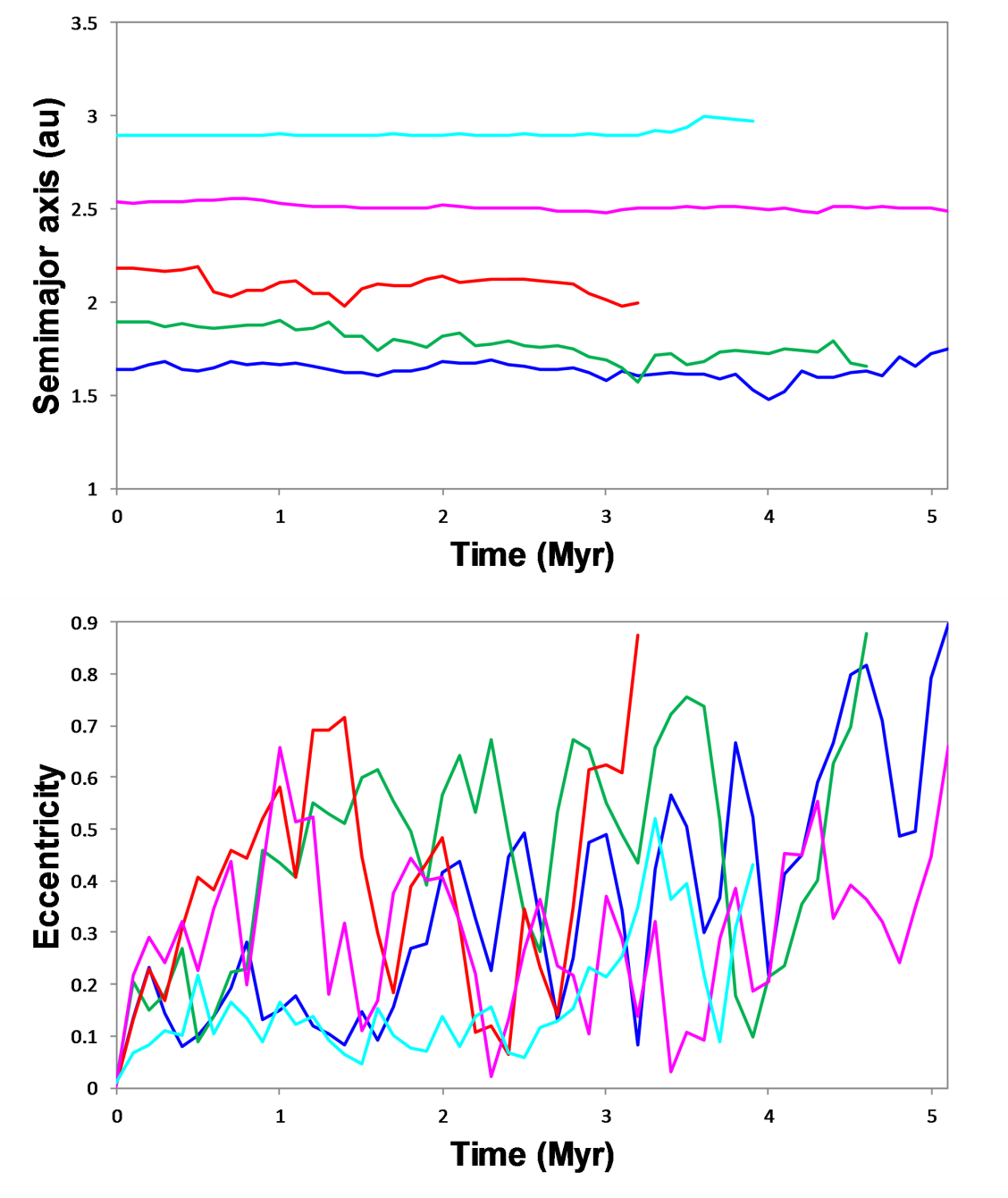


**Figure S3.** Orbital evolution of five selected asteroids of system #1 (Figure 2) that were lost from the solar system due to chaotic excitation induced by a near 2:1 MMR Jupiter–Saturn before the giant planet instability/migration. The asteroids also gravitationally interacted with the embryos and planets in formation, as indicated by changes in the semimajor axis.


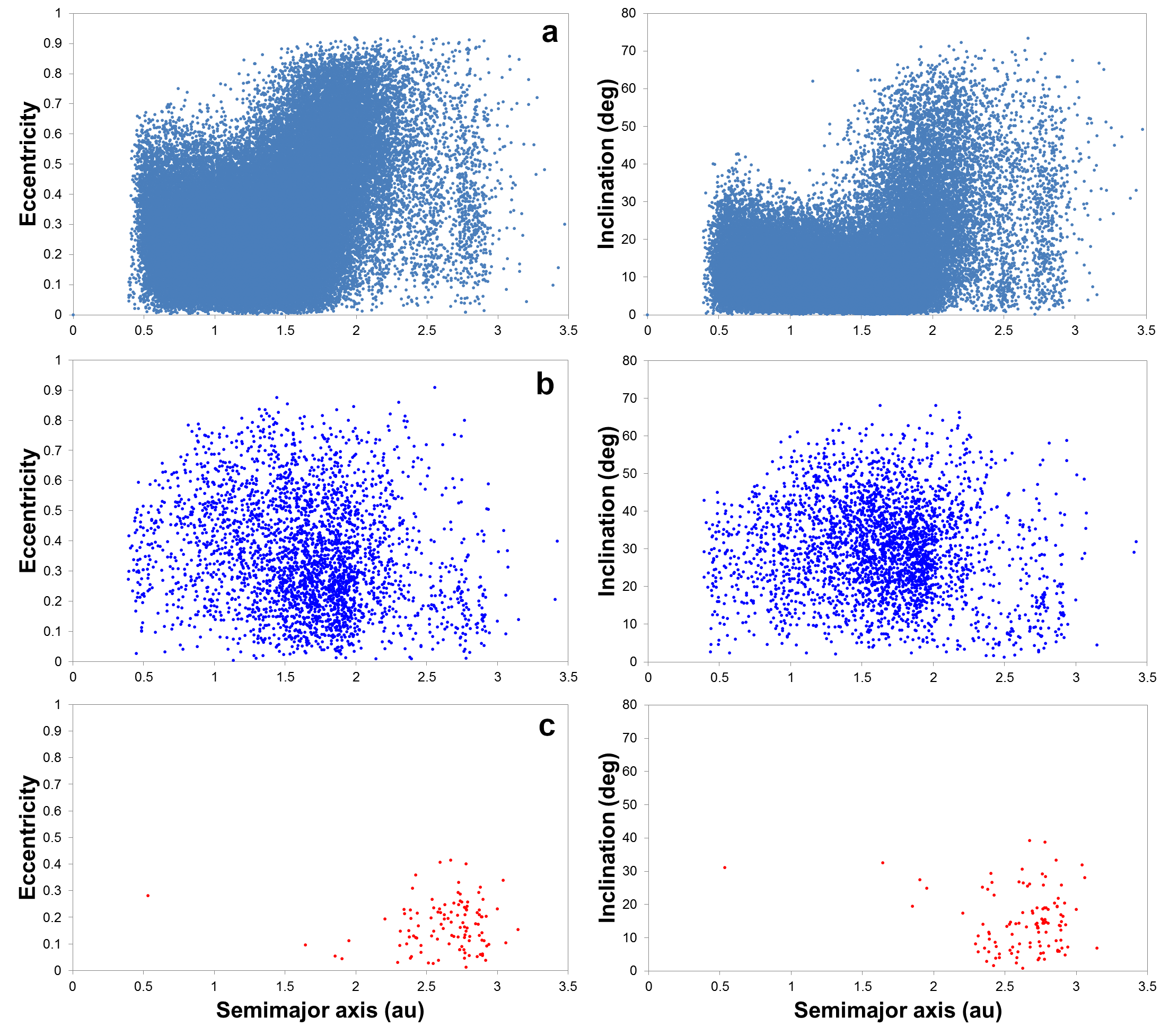


**Figure S4.** Composite of the orbital evolution of embryos and planetesimals initially placed on dynamically cold orbits at ~0.5–3.5 au in our standard protoplanetary disk (46 systems combined from disks A, B and C). **a)** After 5–20 Myr of chaotic excitation generated by near-2:1 MMR Jupiter–Saturn, the disk was strongly perturbed beyond ~1–1.5 au. **b)** After a further 100 Myr of post-instability evolution with the giant planets on their current orbits. **c)** After a further 4 Gyr of dynamical evolution with all the planets on their current orbits. Only local asteroids are shown. See Methods and Supplementary Information for more details.


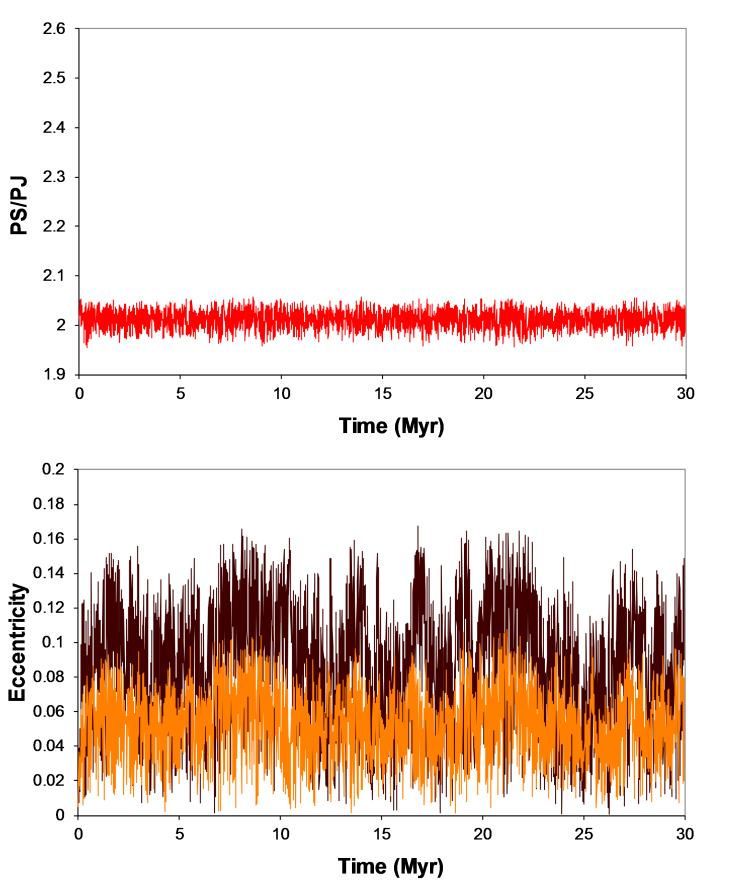


**Figure S5***.* An example of how low-*e* Jupiter (*e*_0,J_ = 0.005; orange curves) and Saturn (*e*_0,S_ = 0.009; brown curves) locked in their mutual 2:1 MMR can evolve to a near-resonant configuration that generates chaotic behaviour for both planets. PS and PJ represent the orbital period of Saturn and Jupiter, respectively. Here, the simulation considered Jupiter, Saturn, and one 5 ME large body placed at 10.3 au outside Saturn’s initial orbit (*a*_0,S_ = 8.92 au). The object suffered gravitational scattering by Saturn at ~0.09 Myr and later diffused outwards until ejection from the system at 2.77 Myr. As a result, Jupiter and Saturn experienced chaotic orbital behaviour near the 2:1 MMR from 0.1 Myr until the end of the simulation at 50 Myr.


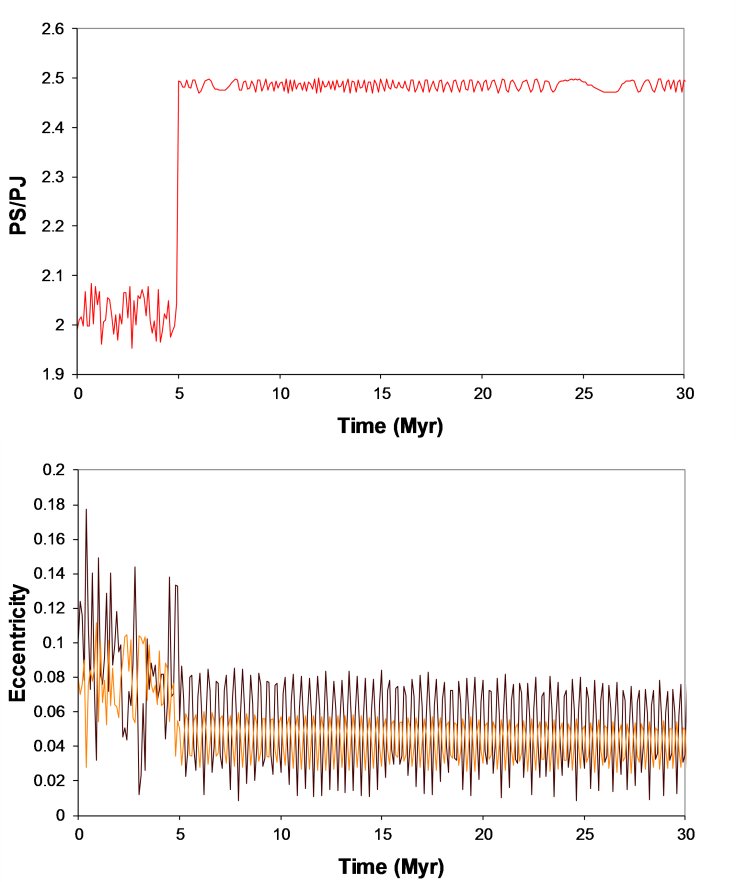

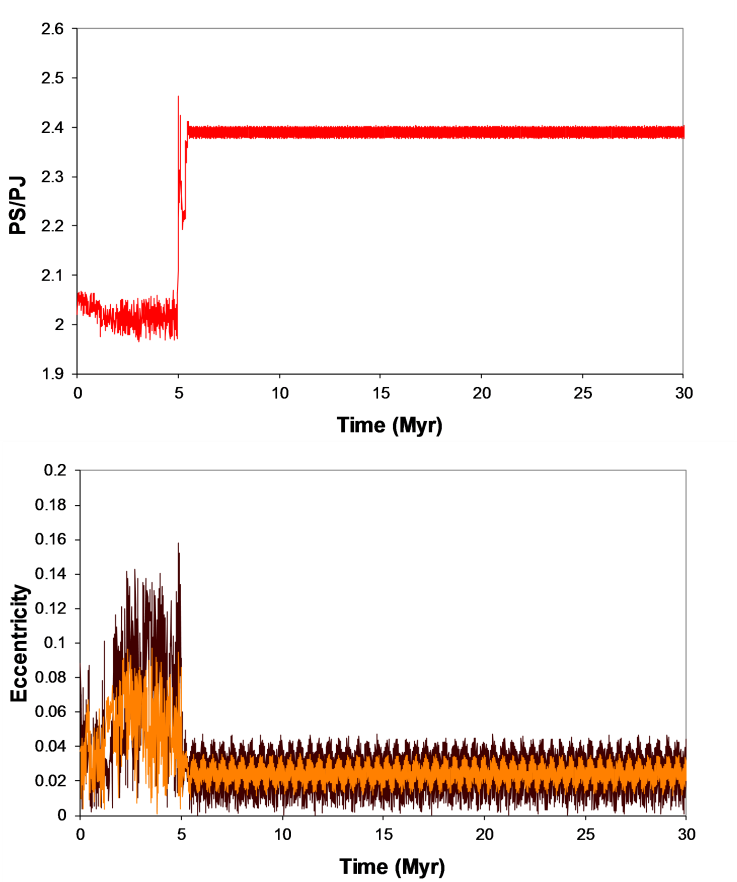


**Figure S6***.* The typical orbital evolution of Jupiter (orange curves) and Saturn (brown curves) in our terrestrial planet formation model for an assumed instability timing of 5 Myr (left panels) and a self-consistent simulation containing Jupiter, Saturn (*e*_0,J_ = *e*_0,S_ ~ 0.04), and three icy giant planets initially locked in mutual MMRs (right panels). PS and PJ represent the orbital period of Saturn and Jupiter, respectively. In the latter simulation, the five giant planets were locked in a 2:1, 4:3, 3:2, 3:2 resonant chain. In both cases, the Jupiter–Saturn pair experiences a chaotic orbital behaviour near the 2:1 MMR during the first ~5 Myr of evolution, after which the giant planet instability occurs (spontaneously in the latter simulation). Also, the PS/PJ evolution is consistent regardless of the assumed initial instantaneous PS/PJ in both simulations.


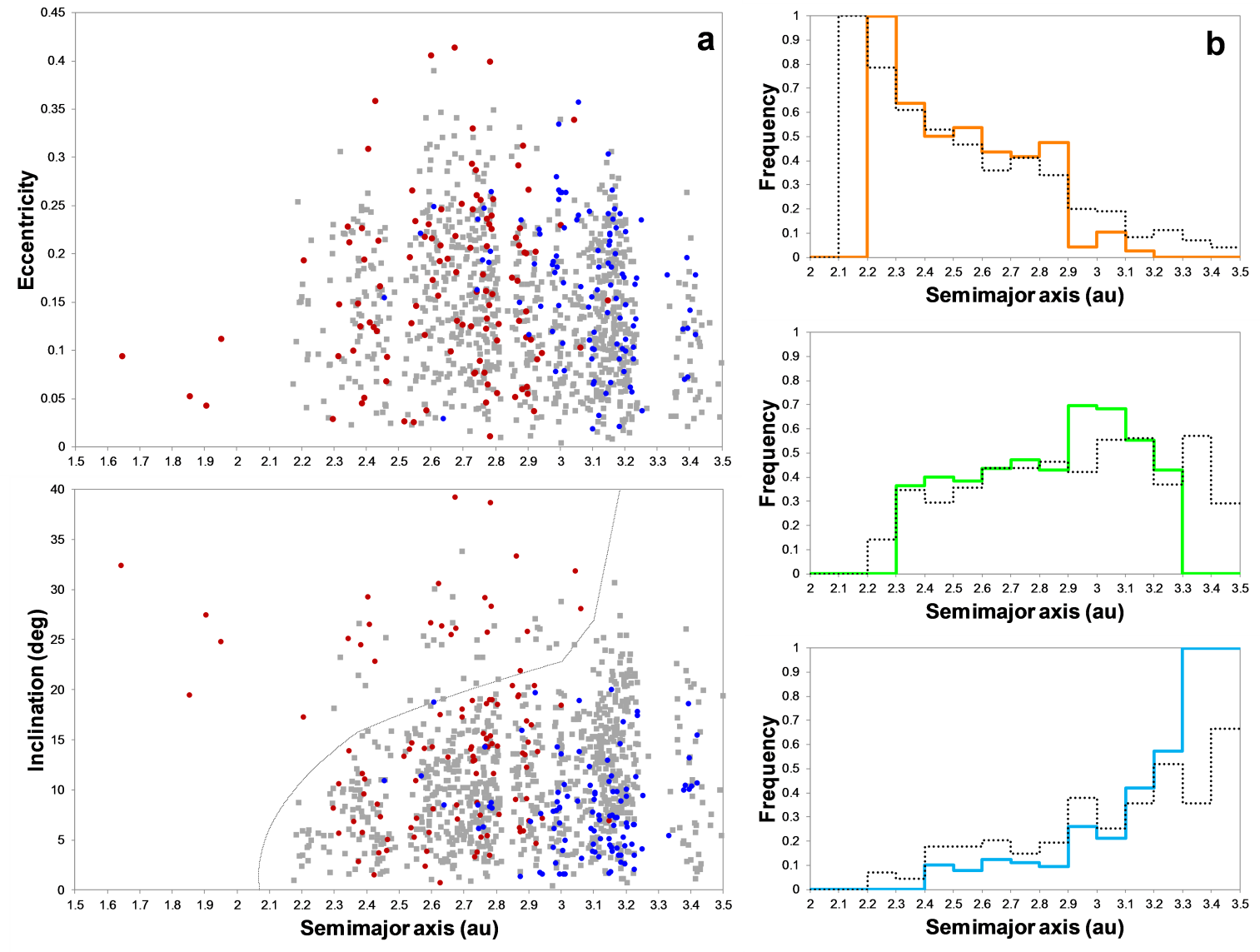


**Figure S7**. The same as Fig. 4, except that the proportion of local:captured asteroids is 50%:50%, and the composition gradient of captured asteroids is represented by C50%-DP50%.

**
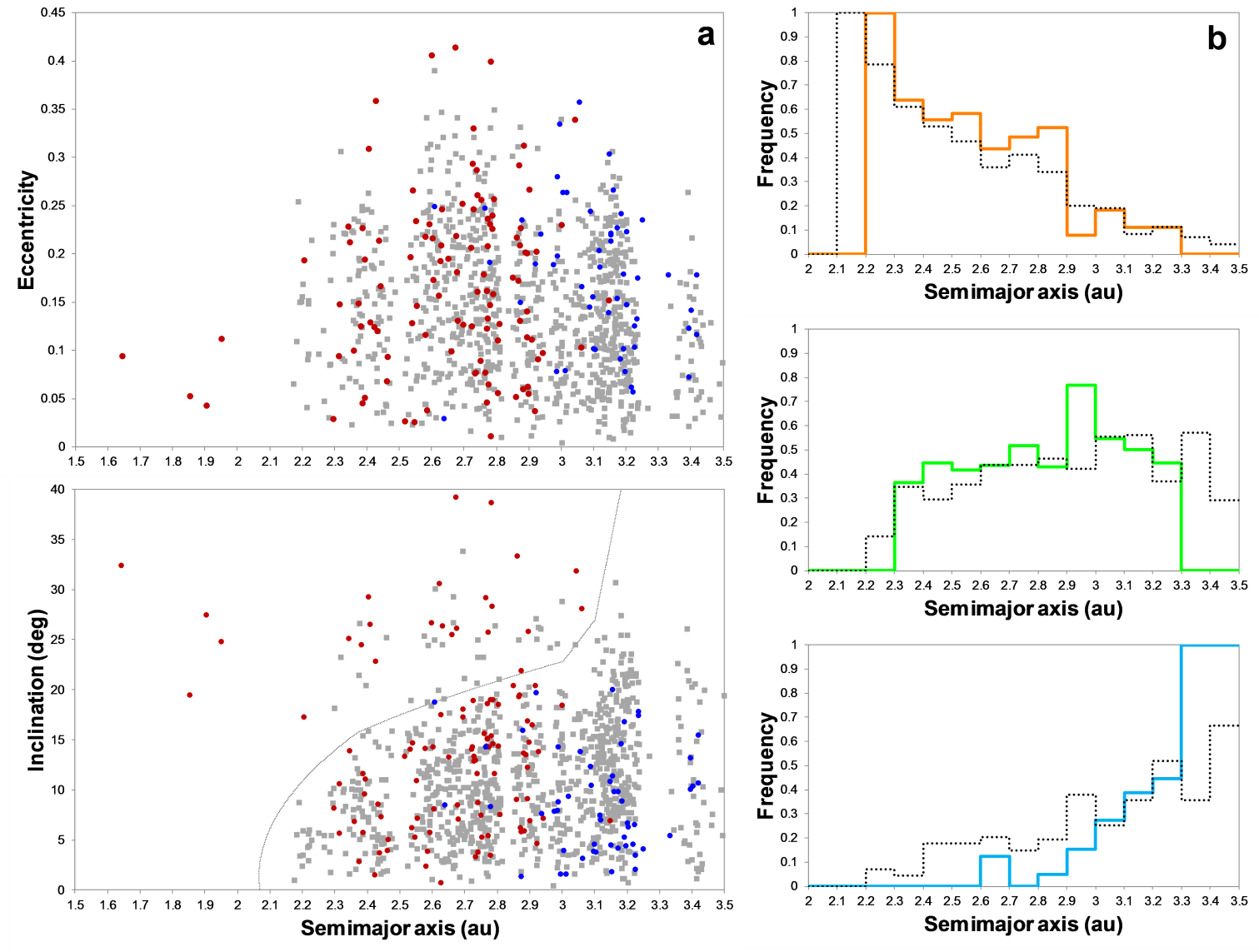
**

**Figure S8***.* The same as Fig. 4, except that the proportion of local:captured asteroids is 67%:33%.

# Supplementary Tables

**Table S1.** Initial conditions in our simulations

| Disk | n. embryos  [m_emb (M_⊕_)] | n. planetesimals (including asteroids) | n. of asteroids (2-3.5 au) | Total mass AB (M_⊕_) | Embryos  a range (au) | *r* in the inner and core regions | surface density slopes in the inner, core and outer regions | inner region (au)  [total mass (M_⊕_)] | core region (au) [total mass (M_⊕_)] | outer region (au) [total mass (M_⊕_)] | instability time (Myr) | post-instability runs |
| --- | --- | --- | --- | --- | --- | --- | --- | --- | --- | --- | --- | --- |
| A | 153 [0.005] | 6220 | 2068 | 2.07 | 0.5-1.2 | 2, 0.5 | 7, -2, -2 | 0.5-0.8 [0.40] | 0.8-1.2 [1.50] | 1.2-3.5 [3.96] | 10, 20 | 50, 50 |
| B | 155 [0.005] | 5441 | 1075 | 0.54 | 0.5-1.2 | 2, 0.5 | 8, -3(-1*), -3 | 0.5-0.8 [0.20] | 0.8-1.2 [1.92]* | 1.2-3.5 [1.37] | 10, 20 | 50, 50 |
| C | 105 [0.01] | 4380 | 1150 | 0.87 | 0.45-1.2 | 2, 1 | 4, -4(0*), -2 | 0.4-0.8 [0.24] | 0.8-1.2 [1.90]* | 1.2-3.5 [1.67] | 5-10 | 100 |
| D | 241 [0.005] | 5312 | 1232 | 0.94 | 0.4-1.5 | 2, 0.5 | 4, -3, -3 | 0.4-0.9 [0.85] | 0.9-1.5 [1.94] | 1.5-3.5 [1.67] | 10 | 50 |
| E | 21 [0.078] | 4738 | 2191 | 2.18 | 0.5-1.5 | 4, 1 | -1.5, -1.5, -1.5 | 0.5-1.0 [1.40] | 1.0-1.5 [1.07] | 1.5-3.5 [3.09] | 5-10 | 50 |
| Ia | 36 [0.02] | 4404 | 994 | 1.24 | 0.4-1.15 | 2, 0.5 | 2, -2(-1*), -2 | 0.3-0.85 [0.24] | 0.85-1.15 [1.70]* | 1.15-3.5 [2.47] | 5 | 50 |
| Ib | 35 [0.03] | 3767 | 994 | 1.24 | 0.4-1.15 | 4, 1 | 2, -2(-1*), -2 | 0.3-0.85 [0.26] | 0.85-1.15 [1.70]* | 1.15-3.5 [2.47] | 5 | 50 |
| Ic | 68 [0.01] | 4476 | 994 | 1.24 | 0.35-1.15 | 1, 0.5 | 0.5, -2(-1*), -2 | 0.3-0.85 [0.22] | 0.85-1.15 [1.70]* | 1.15-3.5 [2.47] | 5 | 50 |
| Id | 40 [0.02] | 4483 | 994 | 1.24 | 0.35-1.15 | 2, 0.5 | 0.5, -2(-1*), -2 | 0.3-0.85 [0.36] | 0.85-1.15 [1.70]* | 1.15-3.5 [2.47] | 5 | 50 |
| Ie | 38 [0.03] | 3811 | 994 | 1.24 | 0.35-1.15 | 4, 1 | 0.5, -2(-1*), -2 | 0.3-0.85 [0.37] | 0.85-1.15 [1.70]* | 1.15-3.5 [2.47] | 5 | 50 |

**Notes.** AB, asteroid belt; *r*, the ratio of total mass in embryos to that in planetesimals. We placed the embryos in the inner and core regions and planetesimals within all disk regions. Planetesimals located at 2–3.5 au represented the primordial asteroid belt. The embryos started with the same mass (m_emb) in a given disk model. The mass of each planetesimal was 5 × 10^−4^ ME in the inner and core regions and within 5–10 × 10^−4^ ME in the outer region. See Methods for other details. A summary of the initial disk conditions is illustrated in Figure S1.

* The following disks possessed mass concentration within the core region. Of the total mass in the core region, 1.50 ME was concentrated within 0.8–1.0 au for disk B (slope −1), 1.54 ME was concentrated within 0.8–1.0 au for disk C (slope 0), and ~1.28 ME was concentrated within 0.85–0.95 au for disks Ia–e (slope −1).

**Table S2.** Initial water mass fractions (WMFs) for embryos and planetesimals located initially in distinct regions of the protoplanetary disk and final WMFs acquired by planet analogues in the 3- and 4-terrestrial planet analogue systems

| # | <1.5 au | 1.5-2 au | 2-2.5 au | >2.5 au | >3 au | Mercury | Venus | Earth | Mars | Result |
| --- | --- | --- | --- | --- | --- | --- | --- | --- | --- | --- |
| 1 | 1.0×10^−5^ | 1.0×10^−5^ | 1.0×10^−3^ | 5.0×10^−2^ |  | 1.2×10^−5^ | 5.5×10^−5^ | 6.9×10^−5^ | 2.0×10^−5^ |  |
| 2 | 1.0×10^−5^ | 1.0×10^−5^ | 1.0×10^−3^ | 1.0×10^−1^ |  | 1.2×10^−5^ | 9.4×10^−5^ | 1.2×10^−4^ | 2.0×10^−5^ |  |
| 3 | 1.0×10^−5^ | 1.0×10^−5^ | 1.0×10^−3^ | 3.0×10^−1^ |  | 1.2×10^−5^ | 2.5×10^−4^ | 3.1×10^−4^ | 2.0×10^−5^ |  |
| 4 | 1.0×10^−5^ | 1.0×10^−5^ | 1.0×10^−2^ | 5.0×10^−2^ |  | 3.2×10^−5^ | 1.1×10^−4^ | 1.2×10^−4^ | 1.2×10^−4^ |  |
| 5 | 1.0×10^−5^ | 1.0×10^−5^ | 1.0×10^−2^ | 1.0×10^−1^ |  | 3.2×10^−5^ | 1.4×10^−4^ | 1.7×10^−4^ | 1.2×10^−4^ |  |
| 6 | 1.0×10^−5^ | 1.0×10^−5^ | 1.0×10^−2^ | 3.0×10^−1^ |  | 3.2×10^−5^ | 3.0×10^−4^ | 3.9×10^−4^ | 1.2×10^−4^ |  |
| 7 | 1.0×10^−5^ | 1.0×10^−5^ | 3.0×10^−2^ | 1.0×10^−1^ |  | 7.5×10^−5^ | 2.4×10^−4^ | 2.7×10^−4^ | 3.1×10^−4^ |  |
| 8 | 1.0×10^−5^ | 1.0×10^−5^ | 3.0×10^−2^ | 2.0×10^−1^ |  | 7.5×10^−5^ | 3.4×10^−4^ | 3.9×10^−4^ | 3.3×10^−4^ |  |
| **9** | **1.0×10^−5^** | **1.0×10^−5^** | **3.0×10^−2^** | **3.0×10^−1^** |  | **7.5×10^−5^** | **4.0×10^−4^** | **5.0×10^−4^** | **3.3×10^−4^** | **OK** |
| **10** | **1.0×10^−5^** | **1.0×10^−5^** | **3.0×10^−2^** | **4.0×10^−1^** |  | **7.5×10^−5^** | **4.7×10^−4^** | **6.0×10^−4^** | **3.3×10^−4^** | **OK** |
| 11 | 1.0×10^−5^ | 1.0×10^−5^ | 5.0×10^−2^ | 5.0×10^−2^ |  | 1.2×10^−4^ | 2.9×10^−4^ | 3.0×10^−4^ | 4.0×10^−4^ |  |
| 12 | 1.0×10^−5^ | 1.0×10^−5^ | 5.0×10^−2^ | 1.0×10^−1^ |  | 1.2×10^−4^ | 3.3×10^−4^ | 3.7×10^−4^ | 4.5×10^−4^ |  |
| **13** | **1.0×10^−5^** | **1.0×10^−5^** | **5.0×10^−2^** | **3.0×10^−1^** |  | **1.2×10^−4^** | **5.4×10^−4^** | **6.0×10^−4^** | **5.4×10^−4^** | **OK (wet Venus)** |
| **14** | **1.0×10^−5^** | **1.0×10^−5^** | **1.0×10^−1^** | **1.0×10^−1^** |  | **2.3×10^−4^** | **5.7×10^−4^** | **5.8×10^−4^** | **7.9×10^−4^** | **OK (wet Venus)** |
| **15** | **1.0×10^−5^** | **1.0×10^−5^** | **1.0×10^−1^** | **3.0×10^−1^** |  | **2.3×10^−4^** | **7.4×10^−4^** | **8.3×10^−4^** | **1.0×10^−3^** | **OK (wet Venus)** |
| 16 | 1.0×10^−5^ | 1.0×10^−4^ | 1.0×10^−3^ | 5.0×10^−2^ | 1.0×10^−1^ | 2.4×10^−5^ | 5.9×10^−5^ | 7.9×10^−5^ | 2.8×10^−5^ |  |
| 17 | 1.0×10^−5^ | 1.0×10^−4^ | 1.0×10^−3^ | 1.0×10^−1^ |  | 2.4×10^−5^ | 9.8×10^−5^ | 1.2×10^−4^ | 2.8×10^−5^ |  |
| 18 | 1.0×10^−5^ | 1.0×10^−4^ | 1.0×10^−3^ | 3.0×10^−1^ |  | 2.4×10^−5^ | 2.6×10^−4^ | 3.2×10^−4^ | 2.8×10^−5^ |  |
| 19 | 1.0×10^−5^ | 1.0×10^−4^ | 1.0×10^−2^ | 5.0×10^−2^ |  | 3.9×10^−5^ | 1.1×10^−4^ | 1.3×10^−4^ | 1.2×10^−4^ |  |
| 20 | 1.0×10^−5^ | 1.0×10^−4^ | 1.0×10^−2^ | 1.0×10^−1^ |  | 3.9×10^−5^ | 1.5×10^−4^ | 1.8×10^−4^ | 1.2×10^−4^ |  |
| 21 | 1.0×10^−5^ | 1.0×10^−4^ | 1.0×10^−2^ | 3.0×10^−1^ |  | 3.9×10^−5^ | 3.0×10^−4^ | 3.9×10^−4^ | 1.2×10^−4^ |  |
| 22 | 1.0×10^−5^ | 1.0×10^−4^ | 5.0×10^−2^ | 5.0×10^−2^ |  | 1.3×10^−4^ | 2.9×10^−4^ | 3.0×10^−4^ | 4.1×10^−4^ |  |
| 23 | 1.0×10^−5^ | 1.0×10^−4^ | 5.0×10^−2^ | 1.0×10^−1^ |  | 1.3×10^−4^ | 3.3×10^−4^ | 3.7×10^−4^ | 4.7×10^−4^ |  |
| **24** | **1.0×10^−5^** | **1.0×10^−4^** | **5.0×10^−2^** | **3.0×10^−1^** |  | **1.3×10^−4^** | **5.4×10^−4^** | **6.1×10^−4^** | **5.5×10^−4^** | **OK (wet Venus)** |
| **25** | **1.0×10^−5^** | **1.0×10^−4^** | **1.0×10^−1^** | **1.0×10^−1^** |  | **2.3×10^−4^** | **5.7×10^−4^** | **5.9×10^−4^** | **8.0×10^−4^** | **OK (wet Venus)** |
| **26** | **1.0×10^−5^** | **1.0×10^−4^** | **1.0×10^−1^** | **3.0×10^−1^** |  | **2.3×10^−4^** | **7.5×10^−4^** | **8.3×10^−4^** | **1.0×10^−3^** | **OK (wet Venus)** |
| **27** | **1.0×10^−5^** | **5.0×10^−3^** | **4.3×10^−2^** | **6.5×10^−2^** | **1.2×10^−1^** | **6.4×10^−4^** | **5.2×10^−4^** | **5.9×10^−4^** | **8.0×10^−4^** | **OK (wet Venus)** |
| **28** | **1.0×10^−5^** | **1.0×10^−2^** | **7.5×10^−2^** | **1.1×10^−1^** | **1.9×10^−1^** | **1.2×10^−3^** | **9.6×10^−4^** | **1.1×10^−3^** | **1.5×10^−3^** | **OK (wet Venus)** |
| 29 | 1.0×10^−4^ | 1.0×10^−4^ | 1.0×10^−3^ | 5.0×10^−2^ |  | 1.0×10^−4^ | 1.4×10^−4^ | 1.6×10^−4^ | 1.1×10^−4^ |  |
| 30 | 1.0×10^−4^ | 1.0×10^−4^ | 1.0×10^−3^ | 1.0×10^−1^ |  | 1.0×10^−4^ | 1.8×10^−4^ | 2.0×10^−4^ | 1.1×10^−4^ |  |
| 31 | 1.0×10^−4^ | 1.0×10^−4^ | 1.0×10^−3^ | 3.0×10^−1^ |  | 1.0×10^−4^ | 3.4×10^−4^ | 4.0×10^−4^ | 1.1×10^−4^ |  |
| 32 | 1.0×10^−4^ | 1.0×10^−4^ | 1.0×10^−2^ | 5.0×10^−2^ |  | 1.2×10^−4^ | 2.0×10^−4^ | 2.1×10^−4^ | 2.0×10^−4^ |  |
| 33 | 1.0×10^−4^ | 1.0×10^−4^ | 1.0×10^−2^ | 1.0×10^−1^ |  | 1.2×10^−4^ | 2.3×10^−4^ | 2.6×10^−4^ | 2.0×10^−4^ |  |
| 34 | 1.0×10^−4^ | 1.0×10^−4^ | 1.0×10^−2^ | 3.0×10^−1^ |  | 1.2×10^−4^ | 3.8×10^−4^ | 4.8×10^−4^ | 2.0×10^−4^ |  |
| 35 | 1.0×10^−4^ | 1.0×10^−4^ | 3.0×10^−2^ | 1.0×10^−1^ |  | 1.6×10^−4^ | 3.3×10^−4^ | 3.6×10^−4^ | 4.0×10^−4^ |  |
| 36 | 1.0×10^−4^ | 1.0×10^−4^ | 3.0×10^−2^ | 2.0×10^−1^ |  | 1.6×10^−4^ | 4.3×10^−4^ | 4.8×10^−4^ | 4.2×10^−4^ |  |
| **37** | **1.0×10^−4^** | **1.0×10^−4^** | **3.0×10^−2^** | **3.0×10^−1^** |  | **1.6×10^−4^** | **4.9×10^−4^** | **5.9×10^−4^** | **4.2×10^−4^** | **OK** |
| **38** | **1.0×10^−4^** | **1.0×10^−4^** | **3.0×10^−2^** | **4.0×10^−1^** |  | **1.6×10^−4^** | **5.6×10^−4^** | **6.9×10^−4^** | **4.2×10^−4^** | **OK (wet Venus)** |
| 39 | 1.0×10^−4^ | 1.0×10^−4^ | 5.0×10^−2^ | 5.0×10^−2^ |  | 2.1×10^−4^ | 3.8×10^−4^ | 3.9×10^−4^ | 4.9×10^−4^ |  |
| 40 | 1.0×10^−4^ | 1.0×10^−4^ | 5.0×10^−2^ | 1.0×10^−1^ |  | 2.1×10^−4^ | 4.2×10^−4^ | 4.6×10^−4^ | 5.4×10^−4^ |  |
| **41** | **1.0×10^−4^** | **1.0×10^−4^** | **5.0×10^−2^** | **3.0×10^−1^** |  | **2.1×10^−4^** | **6.2×10^−4^** | **6.9×10^−4^** | **6.3×10^−4^** | **OK (wet Venus)** |
| **42** | **1.0×10^−4^** | **1.0×10^−4^** | **1.0×10^−1^** | **1.0×10^−1^** |  | **3.2×10^−4^** | **6.6×10^−4^** | **6.7×10^−4^** | **8.8×10^−4^** | **OK (wet Venus)** |
| **43** | **1.0×10^−4^** | **1.0×10^−4^** | **1.0×10^−1^** | **3.0×10^−1^** |  | **3.2×10^−4^** | **8.3×10^−4^** | **9.2×10^−4^** | **1.1×10^−3^** | **OK (wet Venus)** |
| 44 | 1.0×10^−4^ | 1.0×10^−3^ | 1.0×10^−3^ | 5.0×10^−2^ |  | 1.9×10^−4^ | 1.9×10^−4^ | 2.2×10^−4^ | 2.1×10^−4^ |  |
| 45 | 1.0×10^−4^ | 1.0×10^−3^ | 1.0×10^−3^ | 1.0×10^−1^ |  | 1.9×10^−4^ | 2.3×10^−4^ | 2.7×10^−4^ | 2.1×10^−4^ |  |
| 46 | 1.0×10^−4^ | 1.0×10^−3^ | 1.0×10^−3^ | 3.0×10^−1^ |  | 1.9×10^−4^ | 3.9×10^−4^ | 4.9×10^−4^ | 2.1×10^−4^ |  |
| 47 | 1.0×10^−4^ | 1.0×10^−3^ | 1.0×10^−2^ | 5.0×10^−2^ |  | 2.4×10^−4^ | 2.4×10^−4^ | 2.7×10^−4^ | 2.8×10^−4^ |  |
| 48 | 1.0×10^−4^ | 1.0×10^−3^ | 1.0×10^−2^ | 1.0×10^−1^ |  | 2.4×10^−4^ | 2.7×10^−4^ | 3.3×10^−4^ | 2.8×10^−4^ |  |
| **49** | **1.0×10^−4^** | **1.0×10^−3^** | **1.0×10^−2^** | **3.0×10^−1^** |  | **2.4×10^−4^** | **4.2×10^−4^** | **5.4×10^−4^** | **2.8×10^−4^** | **OK** |
| 50 | 1.0×10^−4^ | 1.0×10^−3^ | 3.0×10^−2^ | 1.0×10^−1^ |  | 2.7×10^−4^ | 3.8×10^−4^ | 4.2×10^−4^ | 4.7×10^−4^ |  |
| **51** | **1.0×10^−4^** | **1.0×10^−3^** | **3.0×10^−2^** | **2.0×10^−1^** |  | **2.7×10^−4^** | **4.8×10^−4^** | **5.5×10^−4^** | **5.0×10^−4^** | **OK** |
| **52** | **1.0×10^−4^** | **1.0×10^−3^** | **3.0×10^−2^** | **3.0×10^−1^** |  | **2.7×10^−4^** | **5.3×10^−4^** | **6.6×10^−4^** | **5.0×10^−4^** | **OK (wet Venus)** |
| **53** | **1.0×10^−4^** | **1.0×10^−3^** | **3.0×10^−2^** | **4.0×10^−1^** |  | **2.7×10^−4^** | **6.1×10^−4^** | **7.7×10^−4^** | **5.0×10^−4^** | **OK (wet Venus)** |
| 54 | 1.0×10^−4^ | 1.0×10^−3^ | 5.0×10^−2^ | 5.0×10^−2^ |  | 2.8×10^−4^ | 4.3×10^−4^ | 4.3×10^−4^ | 5.5×10^−4^ |  |
| **55** | **1.0×10^−4^** | **1.0×10^−3^** | **5.0×10^−2^** | **1.0×10^−1^** |  | **2.8×10^−4^** | **4.6×10^−4^** | **5.0×10^−4^** | **6.6×10^−4^** | **OK** |
| **56** | **1.0×10^−4^** | **1.0×10^−3^** | **5.0×10^−2^** | **3.0×10^−1^** |  | **2.8×10^−4^** | **6.7×10^−4^** | **7.7×10^−4^** | **7.0×10^−4^** | **OK (wet Venus)** |
| **57** | **1.0×10^−4^** | **1.0×10^−3^** | **1.0×10^−1^** | **1.0×10^−1^** |  | **3.9×10^−4^** | **7.1×10^−4^** | **7.1×10^−4^** | **9.4×10^−4^** | **OK (wet Venus)** |
| **58** | **1.0×10^−4^** | **1.0×10^−3^** | **1.0×10^−1^** | **3.0×10^−1^** |  | **3.9×10^−4^** | **8.8×10^−4^** | **9.7×10^−4^** | **1.2×10^−3^** | **OK (wet Venus)** |
| **59** | **1.0×10^−4^** | **5.0×10^−3^** | **5.0×10^−3^** | **1.0×10^−1^** |  | **5.8×10^−4^** | **4.5×10^−4^** | **5.7×10^−4^** | **6.4×10^−4^** | **OK** |
| **60** | **1.0×10^−4^** | **5.0×10^−3^** | **5.0×10^−3^** | **2.0×10^−1^** |  | **5.8×10^−4^** | **5.4×10^−4^** | **6.8×10^−4^** | **6.9×10^−4^** | **OK (wet Venus)** |
| **61** | **1.0×10^−4^** | **5.0×10^−3^** | **5.0×10^−3^** | **3.0×10^−1^** |  | **5.8×10^−4^** | **6.2×10^−4^** | **8.1×10^−4^** | **6.9×10^−4^** | **OK (wet Venus)** |
| **62** | **1.0×10^−4^** | **5.0×10^−3^** | **5.0×10^−3^** | **4.0×10^−1^** |  | **5.8×10^−4^** | **7.0×10^−4^** | **9.1×10^−4^** | **6.9×10^−4^** | **OK (wet Venus)** |
| **63** | **1.0×10^−4^** | **5.0×10^−3^** | **1.0×10^−2^** | **1.0×10^−1^** |  | **5.9×10^−4^** | **4.7×10^−4^** | **5.9×10^−4^** | **7.0×10^−4^** | **OK** |
| **64** | **1.0×10^−4^** | **5.0×10^−3^** | **1.0×10^−2^** | **2.0×10^−1^** |  | **5.9×10^−4^** | **5.6×10^−4^** | **7.1×10^−4^** | **7.2×10^−4^** | **OK (wet Venus)** |
| **65** | **1.0×10^−4^** | **5.0×10^−3^** | **1.0×10^−2^** | **3.0×10^−1^** |  | **5.9×10^−4^** | **6.4×10^−4^** | **8.2×10^−4^** | **7.2×10^−4^** | **OK (wet Venus)** |
| **66** | **1.0×10^−4^** | **5.0×10^−3^** | **2.0×10^−2^** | **5.0×10^−2^** |  | **6.3×10^−4^** | **4.8×10^−4^** | **5.5×10^−4^** | **7.4×10^−4^** | **OK** |
| **67** | **1.0×10^−4^** | **5.0×10^−3^** | **2.0×10^−2^** | **1.0×10^−1^** |  | **6.9×10^−4^** | **5.2×10^−4^** | **6.2×10^−4^** | **7.6×10^−4^** | **OK (wet Venus)** |
| **68** | **1.0×10^−4^** | **5.0×10^−3^** | **2.0×10^−2^** | **2.0×10^−1^** |  | **6.9×10^−4^** | **6.1×10^−4^** | **7.7×10^−4^** | **7.8×10^−4^** | **OK (wet Venus)** |
| **69** | **1.0×10^−4^** | **5.0×10^−3^** | **2.0×10^−2^** | **3.0×10^−1^** |  | **6.9×10^−4^** | **6.9×10^−4^** | **8.8×10^−4^** | **7.8×10^−4^** | **OK (wet Venus)** |
| **70** | **1.0×10^−4^** | **5.0×10^−3^** | **3.0×10^−2^** | **5.0×10^−2^** |  | **6.9×10^−4^** | **5.3×10^−4^** | **6.0×10^−4^** | **7.8×10^−4^** | **OK (wet Venus)** |
| **71** | **1.0×10^−4^** | **5.0×10^−3^** | **3.0×10^−2^** | **1.0×10^−1^** |  | **7.0×10^−4^** | **5.7×10^−4^** | **6.5×10^−4^** | **8.0×10^−4^** | **OK (wet Venus)** |
| **72** | **1.0×10^−4^** | **5.0×10^−3^** | **3.0×10^−2^** | **2.0×10^−1^** |  | **7.0×10^−4^** | **6.7×10^−4^** | **8.1×10^−4^** | **8.5×10^−4^** | **OK (wet Venus)** |
| **73** | **1.0×10^−4^** | **5.0×10^−3^** | **3.0×10^−2^** | **3.0×10^−1^** |  | **7.0×10^−4^** | **7.5×10^−4^** | **9.5×10^−4^** | **8.5×10^−4^** | **OK (wet Venus)** |
| **74** | **1.0×10^−4^** | **5.0×10^−3^** | **3.0×10^−2^** | **4.0×10^−1^** |  | **7.0×10^−4^** | **8.3×10^−4^** | **1.1×10^−3^** | **8.5×10^−4^** | **OK (wet Venus)** |
| **75** | **1.0×10^−4^** | **5.0×10^−3^** | **5.0×10^−2^** | **1.0×10^−1^** |  | **8.1×10^−4^** | **6.6×10^−4^** | **7.5×10^−4^** | **9.6×10^−4^** | **OK (wet Venus)** |
| **76** | **1.0×10^−4^** | **5.0×10^−3^** | **5.0×10^−2^** | **2.0×10^−1^** |  | **8.1×10^−4^** | **7.6×10^−4^** | **8.8×10^−4^** | **1.0×10^−3^** | **OK (wet Venus)** |
| **77** | **1.0×10^−4^** | **5.0×10^−3^** | **5.0×10^−2^** | **3.0×10^−1^** |  | **8.1×10^−4^** | **8.6×10^−4^** | **1.0×10^−3^** | **1.0×10^−3^** | **OK (wet Venus)** |
| **78** | **1.0×10^−4^** | **5.0×10^−3^** | **1.0×10^−1^** | **1.0×10^−1^** |  | **9.4×10^−4^** | **9.1×10^−4^** | **9.5×10^−4^** | **1.3×10^−3^** | **OK (wet Venus)** |
| **79** | **1.0×10^−4^** | **5.0×10^−3^** | **1.0×10^−1^** | **2.0×10^−1^** |  | **9.4×10^−4^** | **9.8×10^−4^** | **1.1×10^−3^** | **1.4×10^−3^** | **OK (wet Venus)** |
| **80** | **1.0×10^−4^** | **5.0×10^−3^** | **1.0×10^−1^** | **3.0×10^−1^** |  | **9.4×10^−4^** | **1.1×10^−3^** | **1.2×10^−3^** | **1.5×10^−3^** | **OK (wet Venus)** |

**Notes.** Successful systems are highlighted in bold. Model 1 was adopted in ref.^4^, the most widely used WMF model in the literature. Model 2 is based on model 1 and was often used in the instability models of ref.^9^. Model 16 was introduced in the review of ref.^3^ and used in the models of ref.^19^. The four columns on the right side show the median WMFs acquired by each of the planet analogues identified in all systems in the standard disk. If the median WMFs of Venus, Earth and Mars simultaneously satisfied the respective observational constraints (0.1–5 × 10^−4^, 5–25 × 10^−4^ and 0.5–20 × 10^−4^, respectively), the WMF model was deemed successful. Here, ‘wet Venus’ refers to the assumption that early Venus was wet by setting its maximum WMF constraint to 5 × 10^−3^. See Methods and Supplementary Information for more details.

**Table S3.** Summary of key variables for the 47 systems simultaneously containing analogues of Mercury, Venus, Earth and Mars

| System # | Disk | AMD | RMC | tLGI (Myr) | Lvf (%) | tMars (Myr) | C1 | C2 | C3 | C4 | C5 | Result |
| --- | --- | --- | --- | --- | --- | --- | --- | --- | --- | --- | --- | --- |
| 1 | D | 0.0094 | 53.4 | 17 | 4.2 | 178.2 | X | O | Δ | Δ | X | 2 |
| 2 | Ib | 0.0008 | 91.9 | 18 | 11.1 | 138.9 | O | O | Δ | X | X | 2 |
| 3 | Ia | 0.0024 | 75.9 | 8 | 21.0 | 373.2 | O | O | X | X | X | 3 |
| **4** | **Ia** | **0.0008** | **75.6** | **146** | **0.7** | **33.9** | **O** | **O** | **O** | **O** | **Δ** | **OK** |
| 5 | Ia | 0.0007 | 80.4 | 3 | 29.5 | 60.4 | O | O | X | X | X | 3 |
| 6 | Ie | 0.0029 | 56.7 | 10 | 12.3 | 22.9 | O | O | X | X | Δ | 2 |
| **7** | **Ib** | **0.0059** | **71.0** | **206** | **0.2** | **9.7** | **Δ** | **O** | **O** | **O** | **O** | **OK** |
| **8** | **Ib** | **0.0033** | **53.3** | **365** | **0.0** | **11.8** | **O** | **O** | **X** | **O** | **O** | **marginally ok** |
| 9 | Id | 0.003 | 52.2 | 2 | 41.2 | 22.6 | O | O | X | X | Δ | 2 |
| **10** | **Ib** | **0.0026** | **53.8** | **53** | **2.5** | **24.0** | **O** | **O** | **O** | **Δ** | **Δ** | **OK** |
| **11** | **Id** | **0.0009** | **49.2** | **36** | **5.7** | **31.0** | **O** | **O** | **O** | **X** | **Δ** | **marginally ok** |
| **12** | **Ic** | **0.0018** | **70.1** | **151** | **0.7** | **282.3** | **O** | **O** | **O** | **O** | **X** | **marginally ok** |
| 13 | A | 0.006 | 68.4 | 12 | 12.6 | 20.2 | Δ | O | X | X | Δ | 2 |
| **14** | **B** | **0.0028** | **102.3** | **18** | **4.0** | **6.7** | **O** | **O** | **Δ** | **Δ** | **O** | **OK** |
| **15** | **C** | **0.0032** | **63.9** | **49** | **0.9** | **7.7** | **O** | **O** | **O** | **O** | **O** | **OK** |
| **16** | **Id** | **0.002** | **50.3** | **166** | **0.4** | **15.0** | **O** | **O** | **O** | **O** | **O** | **OK** |
| **17** | **Id** | **0.0092** | **43.0** | **81** | **0.8** | **27.7** | **X** | **Δ** | **O** | **O** | **Δ** | **marginally ok** |
| **18** | **Ie** | **0.0059** | **48.8** | **22** | **5.2** | **21.7** | **Δ** | **O** | **Δ** | **Δ** | **Δ** | **OK** |
| **19** | **Ib** | **0.0033** | **50.2** | **93** | **0.8** | **25.3** | **O** | **O** | **O** | **O** | **Δ** | **OK** |
| **20** | **Ia** | **0.0054** | **50.4** | **100** | **0.9** | **154.2** | **Δ** | **O** | **O** | **O** | **X** | **marginally ok** |
| **21** | **D** | **0.0021** | **64.7** | **35** | **1.3** | **13.2** | **O** | **O** | **O** | **Δ** | **O** | **OK** |
| **22** | **Ia** | **0.0008** | **77.0** | **45** | **4.4** | **33.9** | **O** | **O** | **O** | **Δ** | **Δ** | **OK** |
| **23** | **B** | **0.0066** | **70.7** | **48** | **1.6** | **201.2** | **Δ** | **O** | **O** | **Δ** | **X** | **marginally ok** |
| 24 | Ic | 0.0016 | 55.9 | 25 | 8.1 | 57.7 | O | O | O | X | X | 2 |
| 25 | Ia | 0.015 | 43.4 | 396 | 0.0 | 46.2 | X | Δ | X | O | X | 3 |
| 26 | Ic | 0.003 | 38.5 | 23 | 10.7 | 33.9 | O | X | Δ | X | Δ | 2 |
| **27** | **Id** | **0.0011** | **40.4** | **54** | **3.7** | **27.2** | **O** | **Δ** | **O** | **Δ** | **Δ** | **OK** |
| 28 | Ia | 0.0009 | 69.5 | 8 | 18.2 | 54.6 | O | O | X | X | X | 3 |
| **29** | **D** | **0.0091** | **52.4** | **51** | **1.0** | **13.5** | **X** | **O** | **O** | **O** | **O** | **marginally ok** |
| **30** | **Ib** | **0.0015** | **52.5** | **138** | **0.2** | **53.2** | **O** | **O** | **O** | **O** | **X** | **marginally ok** |
| **31** | **Ib** | **0.003** | **52.8** | **166** | **0.3** | **12.2** | **O** | **O** | **O** | **O** | **O** | **OK** |
| **32** | **Ib** | **0.005** | **40.0** | **80** | **0.9** | **9.1** | **Δ** | **Δ** | **O** | **O** | **O** | **OK** |
| 33 | C | 0.0011 | 67.0 | 8 | 12.2 | 4.4 | O | O | X | X | O | 2 |
| **34** | **A** | **0.0021** | **61.1** | **43** | **1.3** | **18.4** | **O** | **O** | **O** | **Δ** | **O** | **OK** |
| **35** | **B** | **0.0011** | **56.9** | **30** | **3.2** | **8.1** | **O** | **O** | **O** | **Δ** | **O** | **OK** |
| **36** | **C** | **0.0012** | **67.8** | **56** | **1.0** | **90.9** | **O** | **O** | **O** | **O** | **X** | **marginally ok** |
| **37** | **D** | **0.0014** | **63.5** | **20** | **3.1** | **6.9** | **O** | **O** | **Δ** | **Δ** | **O** | **OK** |
| 38 | Ia | 0.0024 | 63.1 | 10 | 17.6 | 136.1 | O | O | X | X | X | 3 |
| **39** | **Ie** | **0.0024** | **50.7** | **79** | **0.7** | **11.8** | **O** | **O** | **O** | **O** | **O** | **OK** |
| 40 | Id | 0.0012 | 46.4 | 10 | 18.5 | 29.4 | O | O | X | X | Δ | 2 |
| **41** | **C** | **0.0026** | **110.2** | **204** | **0.0** | **161.1** | **O** | **O** | **O** | **O** | **X** | **marginally ok** |
| 42 | Ic | 0.0014 | 51.6 | 13 | 13.5 | 35.3 | O | O | X | X | X | 3 |
| **43** | **Ie** | **0.0031** | **49.2** | **103** | **0.1** | **23.3** | **O** | **O** | **O** | **O** | **Δ** | **OK** |
| 44 | Ib | 0.0008 | 51.0 | 33 | 7.0 | 36.6 | O | O | O | X | X | 2 |
| 45 | C | 0.0032 | 69.1 | 6 | 16.2 | 261.9 | O | O | X | X | X | 3 |
| **46** | **Ie** | **0.0023** | **51.6** | **43** | **2.7** | **19.4** | **O** | **O** | **O** | **Δ** | **Δ** | **OK** |
| **47** | **Ic** | **0.0009** | **55.4** | **64** | **2.3** | **24.3** | **O** | **O** | **O** | **Δ** | **Δ** | **OK** |
|  |  |  |  |  |  |  |  |  |  |  |  |  |
| **Solar system** | **-** | **0.0018** | **89.7** | **25-245** | **<1** | **<15-23** | **-** | **-** | **-** | **-** | **-** |  |

**Notes.** Successful systems are highlighted in bold. AMD, angular momentum deficit; RMC, radial mass concentration; tLGI, the time of the last giant impact of the Earth analogue (defined here by the collision of an object 5% as massive as the target body); Lvf, late veneer mass fraction of the representative Earth analogue; tMars, formation time of Mars analogues. Except for the number of analogue systems, all other quantities are represented by medians in each disk model. ‘O’ and ‘Δ’ indicate that a system fully and marginally satisfied a specific constraint among C1–C5, respectively, while ‘X’ indicates that it did not. The constraints were defined as C1: AMD (0–0.0036), C2: RMC (44.9–179.4), C3: tLGI for the Earth analogue (25–245 Myr), C4: LVf for the Earth analogue (< 1%), C5: Mars analogue formation time (< 15–23 Myr). The water-mass fractions of all Earth analogues satisfied the observed constraints in several reasonable WMF models (Table S2). Most Mars analogues that failed to meet tMars suffered one giant impact by 30–40 Myr, so it was not the result of a continuous accretion history. See Supplementary Information for more details.

**Table S4.** Representative analogues of Mercury, Venus, Earth and Mars formed in all our terrestrial planet analogue systems

|  | N | *a* (au) | *e* | *i* (°) | *m* (M_⊕_) | TF 80% [90%] (Myr) |
| --- | --- | --- | --- | --- | --- | --- |
| *Mercury* |  |  |  |  |  |  |
| A | 7 | 0.510 | 0.103 | 5.616 | 0.144 |  |
| B | 9 | 0.492 | 0.107 | 4.712 | 0.112 |  |
| C | 9 | 0.475 | 0.115 | 3.947 | 0.107 |  |
| D | 9 | 0.492 | 0.133 | 6.947 | 0.188 |  |
| E | 4 | 0.467 | 0.081 | 2.633 | 0.259 |  |
| Ia | 23 | 0.465 | 0.061 | 3.220 | 0.109 |  |
| Ib | 18 | 0.452 | 0.073 | 3.182 | 0.154 |  |
| Ic | 12 | 0.420 | 0.085 | 3.720 | 0.112 |  |
| Id | 15 | 0.448 | 0.072 | 3.715 | 0.203 |  |
| Ie | 16 | 0.400 | 0.106 | 4.356 | 0.139 |  |
| Standard (ABC) | 25 | 0.492 | 0.112 | 4.712 | 0.112 |  |
| A–E | 38 | 0.488 | 0.110 | 4.709 | 0.139 |  |
| Ix | 84 | 0.442 | 0.074 | 3.545 | 0.158 |  |
| 4-P systems | 47 | 0.467 | 0.078 | 3.947 | 0.163 |  |
|  |  |  |  |  |  |  |
| *Venus* |  |  |  |  |  |  |
| A | 21 | 0.649 | 0.040 | 2.222 | 0.925 |  |
| B | 30 | 0.656 | 0.044 | 2.320 | 0.926 |  |
| C | 36 | 0.653 | 0.041 | 2.146 | 0.906 |  |
| D | 13 | 0.697 | 0.048 | 2.165 | 1.157 |  |
| E | 10 | 0.645 | 0.046 | 2.115 | 0.989 |  |
| Ia | 29 | 0.653 | 0.034 | 1.991 | 0.769 |  |
| Ib | 25 | 0.675 | 0.039 | 2.156 | 0.819 |  |
| Ic | 18 | 0.690 | 0.026 | 1.915 | 1.000 |  |
| Id | 21 | 0.667 | 0.035 | 2.114 | 0.822 |  |
| Ie | 18 | 0.663 | 0.040 | 2.347 | 0.993 |  |
| Standard (ABC) | 87 | 0.654 | 0.042 | 2.222 | 0.925 |  |
| A–E | 110 | 0.657 | 0.043 | 2.207 | 0.952 |  |
| Ix | 111 | 0.669 | 0.035 | 2.097 | 0.822 |  |
| 4-P systems | 47 | 0.680 | 0.031 | 2.154 | 0.998 |  |
|  |  |  |  |  |  |  |
| *Earth* |  |  |  |  |  |  |
| A | 21 | 1.067 | 0.050 | 2.027 | 1.196 | 17.7 [24.5] |
| B | 30 | 1.064 | 0.035 | 2.088 | 1.035 | 15.7 [22.4] |
| C | 36 | 1.045 | 0.043 | 2.124 | 0.873 | 18.6 [32.7] |
| D | 13 | 1.137 | 0.052 | 2.568 | 1.030 | 20.2 [28.0] |
| E | 10 | 1.046 | 0.050 | 1.931 | 0.890 | 83.0 [91.2] |
| Ia | 29 | 0.948 | 0.025 | 2.033 | 0.927 | 32.0 [52.4] |
| Ib | 25 | 0.987 | 0.029 | 2.037 | 0.892 | 18.4 [30.5] |
| Ic | 18 | 1.078 | 0.028 | 2.063 | 0.777 | 22.0[33.4] |
| Id | 21 | 0.996 | 0.032 | 2.121 | 0.892 | 21.5 [35.6] |
| Ie | 18 | 1.010 | 0.035 | 2.334 | 0.867 | 19.3 [29.3] |
| Standard (ABC) | 87 | 1.063 | 0.043 | 2.109 | 1.033 | 16.5 [28.1] |
| A–E | 110 | 1.065 | 0.044 | 2.130 | 0.999 | 18.1 [29.8] |
| Ix | 111 | 0.996 | 0.030 | 2.094 | 0.858 | 20.0 [35.6] |
| 4-P systems | 47 | 1.048 | 0.027 | 2.044 | 0.898 | 17.7 [33.2] |
|  |  |  |  |  |  |  |
| *Mars* |  |  |  |  |  |  |
| A | 16 | 1.497 | 0.095 | 6.032 | 0.147 | 14.2 [20.1] |
| B | 24 | 1.560 | 0.099 | 7.491 | 0.130 | 16.9 [20.0] |
| C | 32 | 1.591 | 0.073 | 5.632 | 0.126 | 17.3 [19.2] |
| D | 8 | 1.683 | 0.099 | 5.789 | 0.174 | 10.0 [13.4] |
| E | 6 | 1.622 | 0.097 | 4.635 | 0.119 | 1.0 [9.1] |
| Ia | 14 | 1.479 | 0.057 | 3.367 | 0.212 | 36.1 [41.8] |
| Ib | 16 | 1.596 | 0.073 | 5.989 | 0.228 | 24.0 [24.7] |
| Ic | 11 | 1.691 | 0.038 | 4.546 | 0.128 | 18.1 [33.6] |
| Id | 12 | 1.535 | 0.057 | 3.275 | 0.220 | 13.2 [27.5] |
| Ie | 7 | 1.637 | 0.099 | 5.146 | 0.106 | 6.2 [21.4] |
| Standard (ABC) | 72 | 1.545 | 0.080 | 6.201 | 0.133 | 15.5 [19.7] |
| A–E | 86 | 1.578 | 0.089 | 6.143 | 0.136 | 14.9 [17.9] |
| Ix | 60 | 1.565 | 0.066 | 4.319 | 0.203 | 18.3 [27.5] |
| 4-P systems | 47 | 1.556 | 0.058 | 4.534 | 0.150 | 18.4 [27.2] |
|  |  |  |  |  |  |  |
| **Mercury** | **…** | **0.387** | **0.215** | **6.784** | **0.055** |  |
| **Venus** | **…** | **0.723** | **0.032** | **2.159** | **0.815** |  |
| **Earth** | **…** | **1.000** | **0.028** | **1.988** | **1.000** |  |
| **Mars** | **…** | **1.524** | **0.067** | **4.042** | **0.107** |  |

**Notes.** N, number of identified representative planet analogues; *a*, the semimajor axis; *e*, eccentricity; *i*, inclination; *m*, planet mass; TF, the time the planet acquired 80% or 90% of its final mass. Standard disk (ABC), A–E and Ix show the results for disks combined (e.g., disk Ix refers to the results of disks Ia, Ib, Ic, Id and Ie combined). A–E and Ix represent disks with small and extended inner regions, respectively.

The four representative terrestrial-planet analogues are present in four-planet systems. Four-planet systems are taken altogether when determining the various quantities, regardless of the disk model from which these systems originated. All quantities (except N) represent median values. We averaged the orbital elements of the real planets over the last 100 Myr after integrating their orbits. See Methods and Supplementary Information for more details.

**Table S5.** Summary of key variables obtained for all of our obtained terrestrial-planet analogue systems

| Disk | N0 | n4 | AMD | RMC | tLGI (Myr) | Lvf (%) | tMars (Myr) | C1 (%) | C2 (%) | C3 (%) | C4 (%) | C5 (%) |
| --- | --- | --- | --- | --- | --- | --- | --- | --- | --- | --- | --- | --- |
| A | 100 | 2 | 0.0031 | 61.5 | 26 | 3.3 | 20 | 62 | 95 | 52 | 10 | 33 |
| B | 100 | 3 | 0.0030 | 67.8 | 20 | 4.0 | 20 | 60 | 100 | 40 | 27 | 33 |
| C | 100 | 5 | 0.0030 | 63.4 | 45 | 2.1 | 19 | 61 | 94 | 67 | 44 | 28 |
| D | 50 | 4 | 0.0029 | 56.9 | 33 | 2.1 | 13 | 54 | 100 | 77 | 23 | 38 |
| E | 50 | 0 | 0.0033 | 44.7 | 91 | 0.5 | 9 | 50 | 60 | 70 | 70 | 60 |
| Ia | 50 | 8 | 0.0018 | 55.7 | 67 | 2.1 | 42 | 76 | 93 | 62 | 48 | 0 |
| Ib | 50 | 9 | 0.0023 | 53.3 | 47 | 3.0 | 25 | 76 | 88 | 52 | 44 | 20 |
| Ic | 50 | 5 | 0.0016 | 53.5 | 30 | 6.5 | 34 | 83 | 94 | 72 | 17 | 11 |
| Id | 50 | 6 | 0.0020 | 45.7 | 36 | 5.7 | 27 | 67 | 52 | 52 | 33 | 10 |
| Ie | 50 | 5 | 0.0025 | 50.5 | 32 | 5.1 | 21 | 83 | 89 | 56 | 39 | 17 |
| Standard (ABC) | 300 | 10 | 0.0030 | 64.3 | 30 | 2.9 | 20 | 61 | 97 | 54 | 30 | 31 |
| A–E | 400 | 14 | 0.0030 | 61.8 | 37 | 2.7 | 18 | 59 | 94 | 58 | 33 | 35 |
| Ix | 250 | 33 | 0.0020 | 52.2 | 44 | 4.0 | 27 | 77 | 84 | 59 | 38 | 11 |
| 4-P systems | 650 | 47 | 0.0024 | 55.4 | 43 | 2.7 | 27 | 79 | 89 | 60 | 38 | 30 |
|  |  |  |  |  |  |  |  |  |  |  |  |  |
| **Solar system** | **-** | **-** | **0.0018** | **89.7** | **25-245** | **<1** | **15-23** | **-** | **-** | **-** | **-** | **-** |

**Notes.** N0, the number of simulation runs performed for a given disk model; n4, the number of analogue systems containing Mercury–Venus–Earth–Mars representative analogues. Disk models and other variables are described in the captions of Tables S3 and S4. Except for the number of analogue systems, all other quantities are represented by medians found in each disk model. The last five columns represent the successful fractions of analogue systems that satisfied a particular constraint. See Methods and Supplementary Information for more details.
